# A pan-tissue DNA methylation atlas enables in silico decomposition of human tissue methylomes at cell-type resolution

**DOI:** 10.1038/s41592-022-01412-7

**Published:** 2022-03-11

**Authors:** Tianyu Zhu, Jacklyn Liu, Stephan Beck, Sun Pan, David Capper, Matt Lechner, Chrissie Thirlwell, Charles E. Breeze, Andrew E. Teschendorff

**Affiliations:** 1 CAS Key Laboratory of Computational Biology, Shanghai Institute of Nutrition and Health, University of Chinese Academy of Sciences, Chinese Academy of Sciences, Shanghai, China; 2 University College London, London, UK; 3 UCL Cancer Institute, Paul O’Gorman Building, University College London, London, UK; 4 Department of Cardiac Surgery, Zhongshan Hospital, Fudan University, Shanghai, China; 5 Institut für Neuropathologie, Charité Universitätsmedizin, Berlin, Germany; 6 Charité ‑ Universitätsmedizin Berlin, Corporate Member of Freie Universitat Berlin and Humboldt‑Universitat zu Berlin, Department of Neuropathology, Berlin, Germany; 7 German Cancer Consortium (DKTK), Partner Site Berlin, German Cancer Research Center (DKFZ), Heidelberg, Germany; 8 Department of ENT, Barts Health NHS Trust, London, UK; 9 University of Exeter Medical School, University of Exeter, Exeter, UK; 10 Altius Institute for Biomedical Sciences, Seattle, WA USA

**Keywords:** Computational biology and bioinformatics, Epigenomics

## Abstract

Bulk-tissue DNA methylomes represent an average over many different cell types, hampering our understanding of cell-type-specific contributions to disease development. As single-cell methylomics is not scalable to large cohorts of individuals, cost-effective computational solutions are needed, yet current methods are limited to tissues such as blood. Here we leverage the high-resolution nature of tissue-specific single-cell RNA-sequencing datasets to construct a DNA methylation atlas defined for 13 solid tissue types and 40 cell types. We comprehensively validate this atlas in independent bulk and single-nucleus DNA methylation datasets. We demonstrate that it correctly predicts the cell of origin of diverse cancer types and discovers new prognostic associations in olfactory neuroblastoma and stage 2 melanoma. In brain, the atlas predicts a neuronal origin for schizophrenia, with neuron-specific differential DNA methylation enriched for corresponding genome-wide association study risk loci. In summary, the DNA methylation atlas enables the decomposition of 13 different human tissue types at a high cellular resolution, paving the way for an improved interpretation of epigenetic data.

## Abstract (web summary)

This resource presents an in silico generated DNA methylation atlas that can be used for cell-type deconvolution of human tissues.

## Main

Most epigenome data are generated at the bulk-tissue level, which can confound molecular classifications of disease^1,2^ or prevent the identification of cell-type-specific epigenetic alterations^3,4^. To address these challenges, a number of reference-based and reference-free cell-type deconvolution algorithms have been proposed^5–14^, with reference-based methods offering the greatest potential to identify cell-type-specific DNA methylation (DNAm) changes^2,15,16^. However, a major limitation remains in that these algorithms require DNAm reference profiles representing the main cell types in a given tissue^15,16^. Such DNAm references only exist for tissues such as blood or tissues like saliva or buccal swabs that only contain a few additional cell types^17^. For most human tissues and organs, generating DNAm reference profiles for all underlying cell types is very challenging owing to incomplete knowledge of tissue composition and cell-type-specific markers and because of the high cost and sparsity of single-cell methylomics data^18–21^. To address this problem, we recently showed in a proof-of-principle study focusing on lung and breast tissue, that it is possible to leverage the high-resolution nature of a single-cell RNA-seq atlas to impute a corresponding tissue-specific DNAm reference profile matrix^22^. It is unclear, however, whether this imputation strategy is broadly applicable to other tissue types.

Here we demonstrate that our imputation strategy generalizes, presenting a DNA methylation atlas for 40 cell types that can be used to computationally decompose bulk-tissue DNA methylomes from as many as 13 different tissue types. We comprehensively validate the DNAm-atlas in data from The Cancer Genome Atlas (TCGA)^23^ and other public databases, while demonstrating agreement with competing lower-resolution methods. Notably, the high cellular resolution of our atlas allows new biological inferences and clinical insights to be made across a broad range of complex diseases. For instance, we use the atlas to improve pancreatic cancer diagnosis, to identify the cell of origin of neuroendocrine tumors, to infer cellular compositional changes in aortic dissection and to infer cell-type-specific differential DNAm changes in schizophrenia (SZ). This DNAm-atlas thus constitutes a powerful resource for re-analyzing the large swathes of existing bulk-tissue DNA methylomes in the public domain or for analyzing upcoming DNAm datasets.

## Results

### Construction of the DNAm-atlas

**Fig. 1:**
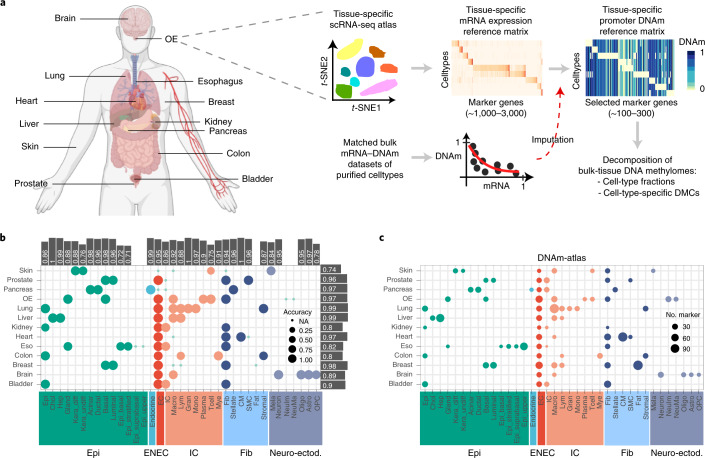
Construction of the DNAm-atlas. **a**, Diagram illustrating the 13 tissue types chosen to build the DNAm-atlas resource, a database of corresponding tissue-specific DNAm reference matrices. Flowchart depicts the construction of a tissue-specific DNAm reference matrix. **b**, Validation of the mRNA expression reference matrices for each tissue and cell type in independent scRNA-seq datasets. Bars indicate the averaged validation accuracy for each cell type and tissue, respectively (top and right of balloon plot). OE, olfactory epithelium; Eso, esophagus. **c**, Balloon plot representing the DNAm-atlas resource, indicating which cell types are represented in each tissue and how many marker genes for each cell type. Source data

We set out to build an atlas of tissue-specific DNAm reference matrices for as many organs and tissues as possible (Fig. 1). Underlying the construction of this atlas is our EpiSCORE algorithm, which performs imputation of DNAm at the promoters of a subset of cell-type-specific marker genes for which DNAm and messenger RNA expression are strongly anticorrelated^22^ (Methods and Fig. 1a). For inclusion in the DNAm-atlas, organs and tissues had to meet the following criteria (1) existence of at least two high-quality single-cell RNA-sequencing (scRNA-seq) atlases, to allow construction and independent validation of a corresponding scRNA-seq reference matrix encompassing at least four cell types; (2) the imputed DNAm reference matrix contains marker genes for each cell type; and (3) existence of independent DNAm datasets (bulk or single cell) to ascertain the validity of the tissue-specific DNAm reference matrix (Methods provides justification of inclusion criteria and parameter choices). In all, we identified 13 tissue types that met all criteria at the time of writing (Fig. 1a). In all cases, the tissue-specific mRNA expression references matrices were validated in independent scRNA-seq datasets (Supplementary Table 1), with reasonably high accuracy and across all underlying cell types (Fig. 1b, Supplementary Table 2 and Supplementary Figs. 1–13). For instance, for 8 out of 13 tissue types, validation accuracy was over 90% (Fig. 1b and Supplementary Figs. 1–13). We then imputed corresponding tissue-specific DNAm reference matrices, with DNAm defined at the promoters of marker genes and for the same cell types as given in the mRNA expression references (Fig. 1c, Supplementary Figs. 1–13 and Supplementary Table 3).

### Systematic validation of the DNAm-atlas

**Fig. 2:**
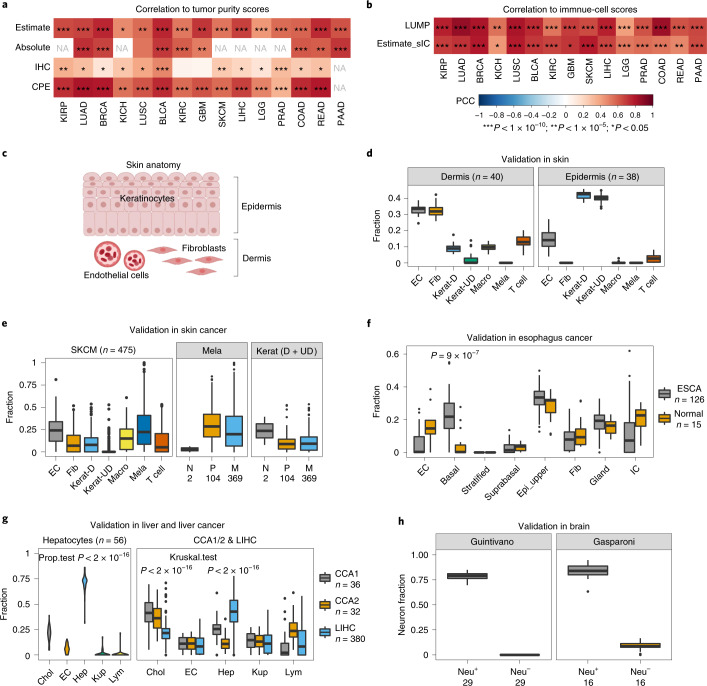
Validation of the DNAm-atlas. **a**,**b**, Systematic validation of the DNAm-atlas tissue-specific DNAm reference matrices in the corresponding Illumina 450k DNAm datasets from TCGA. Heat map depicts the Pearson correlation coefficients (PCCs) between the tumor purity, as estimated using our DNAm-atlas and the tumor purity estimated with different methods, including ESTIMATE, ABSOLUTE (CNV-based), IHC and CPE (**a**). Data as for **a**, but for PCCs between total immune cell fraction, as estimated using DNAm-atlas, and corresponding total immune cell fraction obtained by other methods (ESTIMATE and LUMP) (**b**). * indicates statistical significance level (*P* value thresholds) as shown in **b**. *P* values were derived from a one-tailed correlation test. **c**, Depiction of skin’s anatomy. **d**, Validation of the DNAm reference matrix for skin in healthy bulk skin tissue (Illumina 450k DNAm data) from dermis and epidermis. Box plots display the estimated cell-type fractions for each cell type in the DNAm reference matrix: EC, endothelial cell; Fib, fibroblast; Kerat-D, differentiated keratinocytes; Kerat-UD, undifferentiated keratinocytes; Macro, macrophages; Mela, melanocytes. **e**, Validation of the DNAm reference matrix for skin in the skin cutaneous melanoma TCGA 450k DNAm dataset. Box plot displays the estimated cell-type fractions for each cell type across all primary and metastatic melanomas (left). Box plots display the estimated cell-type fractions for Mela and total keratinocytes (differentiated and undifferentiated) stratified by disease status: N, normal; P, primary melanoma; M, metastatic melanoma (right). **f**, Validation of the esophagus DNAm reference matrix in esophageal cancer (TCGA-ESCA). Box plots display estimated cell-type fractions in healthy and cancer (ESCA) samples. *P* value is from a one-tailed Wilcoxon test comparing the basal fraction between ESCA and healthy samples. **g**, Validation of the DNAm reference matrix for liver in hepatocytes and liver cancer. Box plot displays the estimated cell-type fractions in an Illumina EPIC DNAm dataset of primary hepatocytes for each cell type in the liver DNAm reference matrix: Chol, cholangiocyte; Hep, hepatocyte; Kup, Kupffer cell; Lym, lymphocyte (left). *P* value is from a one-tailed test for proportions. Box plot displays the estimated cell-type fractions in two independent Illumina DNAm datasets profiling cholangiocarcinoma (CCA1 and CCA2) and hepatocellular carcinoma (LIHC) (right). *P* values derive from one-tailed Wilcoxon rank-sum tests, comparing the estimated cholangiocyte fraction in CCA samples to LIHC and vice versa comparing the estimated hepatocyte fraction in LIHC to CCA samples. **h**, Validation of the DNAm reference matrix in purified neuronal (Neu^+^) and non-neuronal (Neu^−^) fractions. Box plots display the estimated neuron fractions in two independent DNAm datasets, profiling FACS-sorted neuronal (Neu^+^) and non-neuronal (Neu^−^) cell populations. In all box plots, the central bar denotes the median, the box width defines the interquartile range (IQR) and whiskers extend to 1.5 × IQR in either direction. Source data

We first aimed to ascertain the overall validity of our DNAm reference matrices in a systematic way by benchmarking cell-type fraction estimates obtained from this atlas against alternative existing tools. We performed this validation in the context of bulk DNAm data from TCGA, by comparing the derived estimates of tumor purity against those obtained using independent methods, which included the gene-expression-based ESTIMATE algorithm^24^, CNV-based ABSOLUTE^25^, immunohistochemistry (IHC) and a method combining all three (consensus purity estimation; CPE)^26^. Tumor purity scores derived from our DNAm-atlas displayed excellent agreement with these benchmarks, especially for the molecular-based ones (Fig. 2a). We also considered a separate total immune cell score, which revealed excellent correlations with gene expression (ESTIMATE)^24^ and DNAm-based (LUMP)^26^ immune cell scores (Fig. 2b). Overall, this demonstrates that our tissue-specific DNAm references can be used to estimate tumor purity or immune cell infiltration, with results that are consistent with current state-of-the-art tools.

The above analysis only validates our DNAm references at a coarse cellular resolution, whereas our DNAm-atlas allows inference of cell-type fractions for all major cell types in the tissue. Thus, to validate the DNAm-atlas at a higher cellular resolution we turned to specific tissue types where suitable independent DNAm data were available for objective testing. For instance, in the context of skin, our DNAm reference matrix was defined over the promoters of 145 marker genes and seven cell types, including endothelial cells, fibroblasts, differentiated and undifferentiated keratinocytes, macrophages, melanocytes and T cells (Supplementary Fig. 5), allowing validation in bulk 450k DNA methylome data from dermis and epidermis^27^; it is well known that the epidermis is composed mainly of keratinocytes, whereas the dermis is predominantly made up of fibroblasts and endothelial cells, with the melanocyte fraction in both layers being very low (Fig. 2c)^28^. In line with this, estimated keratinocyte fractions were high in epidermis and low in dermis, whereas the endothelial and fibroblast fractions exhibited the opposite pattern (Fig. 2d). In addition, the predicted melanocyte fraction in healthy dermis/epidermis was very low (Fig. 2d), whereas in skin cutaneous melanoma (SKCM)^29^ it was much higher (Fig. 2e), as required. The predicted melanocyte fraction was also much higher in the primary and metastatic melanomas compared to healthy skin tissue or when compared to the keratinocyte fractions in the melanomas themselves (Fig. 2e). We also validated the skin DNAm reference matrix in an EPIC DNAm dataset profiling eight skin fibroblast samples^30^ (Supplementary Fig. 14a).

The DNAm-atlas reference matrix for esophagus was defined over various epithelial subtypes, including undifferentiated basal and differentiated upper epithelium in addition to immune and stromal cells (Fig. 1c and Supplementary Fig. 13). To validate the DNAm reference we estimated fractions in the TCGA esophageal cancer dataset^31^, which revealed the expected increase of the undifferentiated fraction in cancer samples (Fig. 2f). The DNAm-atlas reference matrix for liver was defined over hepatocytes, cholangiocytes, endothelial cells, Kupffer cells and lymphocytes (Fig. 1c and Supplementary Fig. 3) and several independent DNAm datasets were available for validation: the liver hepatocellular carcinoma (LIHC, *n* = 380) and cholangiocarcinoma (CCA1, *n* = 36) TCGA datasets^32,33^, another dataset profiling 32 cholangiocarcinomas (CCA2)^34^ and a dataset profiling primary hepatocytes^35^. As required, the primary hepatocyte samples were predicted to be composed mainly of hepatocytes (Fig. 2g). Estimated cell-type fractions across the three independent liver cancer DNAm datasets correctly predicted their cell of origin with higher hepatocyte and cholangiocyte fractions in LIHC and CCA, respectively (Fig. 2g).

The DNAm reference matrix for brain was defined over microglia, endothelial cells, astrocytes, neurons, oligodendrocytes and oligodendrocyte precursor cells (OPCs) (Fig. 1c and Supplementary Fig. 12). To ascertain the validity of this reference we obtained estimates for these cell-type fractions in two independent 450k DNAm datasets that had profiled FACS-sorted neuronal (Neu^+^) and non-neuronal (Neu^−^) populations^36,37^, as well as in an EPIC DNAm dataset profiling 100 Neu^+^ samples^38^. As required, derived neuronal fractions scored consistently high in the neuronal samples (Fig. 2h and Supplementary Fig. 14b). All these results demonstrate that the DNAm reference matrices making up our DNAm-atlas lead to consistent cell-type fraction estimates in bulk DNA methylomes across a wide range of different tissue types.

### DNAm-atlas outperforms one derived from a single-source

We wondered whether our strategy to use high-quality tissue-specific scRNA-seq datasets derived from multiple studies would outperform the alternative of using tissue-specific scRNA-seq datasets from one single study. To this end we focused on the Human Cell Landscape (HCL) scRNA-seq datasets, which were all profiled as part of the same study using the same underlying technology^39^. However, we observed that for skin no scRNA-seq dataset was generated as part of the HCL; for liver the scRNA-seq dataset failed to capture cholangiocytes, a key component of the liver epithelium; for pancreas, the scRNA-seq dataset failed to capture γ and δ endocrine cells, two of the four endocrine cell subtypes and for brain, relatively few neurons were profiled. Nevertheless, we built scRNA-seq and DNAm reference matrices for brain and heart, two tissues for which objective independent validation of the DNAm reference matrices was possible. Using the same validation DNAm datasets for brain considered earlier, we observed that our original DNAm reference matrix validated better than the corresponding one derived from the HCL (Supplementary Fig. 15a). For instance, the purity of the independent Neu^+^ samples was less obvious using the HCL-derived DNAm reference matrices, likely due to the small number of neuronal markers that could be derived from the HCL brain dataset. In the case of heart, we built a five-cell-type mRNA expression reference from a heart-specific Smart-Seq2 scRNA-seq dataset^40^, which we then validated in the 10X scRNA-seq data from the Tabula Muris^41^ (Supplementary Table 1 and Supplementary Fig. 11). Analogously, we built a DNAm reference matrix for the same five cell types by starting out from the HCL heart scRNA-seq dataset. To compare performance of the two DNAm reference matrices, we estimated cell-type fractions in an Illumina 450k DNAm dataset profiling 6 healthy aorta and 12 aortic dissection (AD) samples^42^ and asked whether these fractions predict the well-known increased macrophage and reduced fibroblast proportions in AD^43–46^. Using our DNAm-atlas we were able to correctly predict this increased macrophage to fibroblast ratio, whereas with the HCL-derived matrix we could not (Supplementary Fig. 15b).

### Validation of DNAm brain reference matrix in snmC-seq2 data

**Fig. 3:**
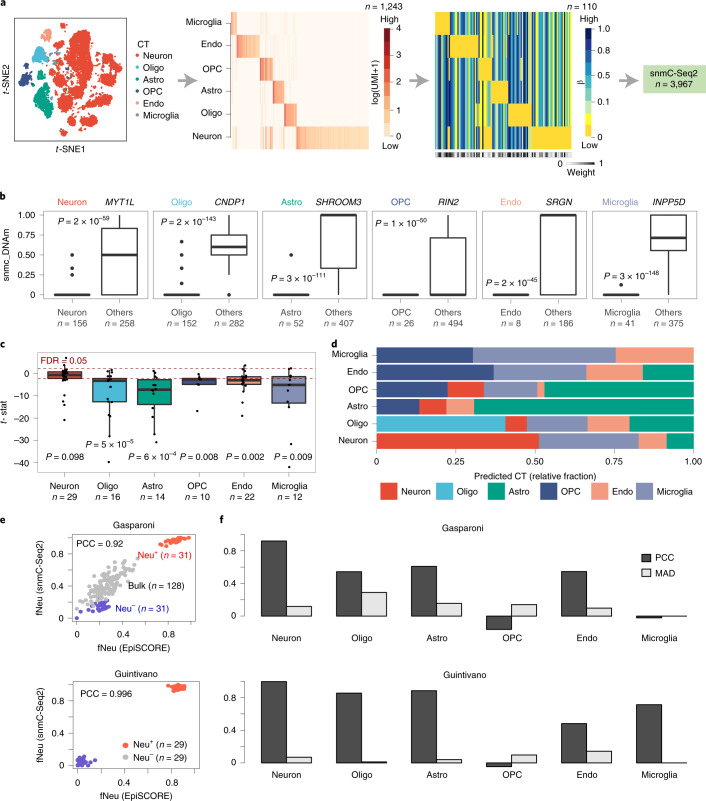
Validation of the brain DNAm-atlas in single-nucleus prefrontal cortex data. **a**, *t*-distributed stochastic neighbor embedding (*t*-SNE) diagram of the scRNA-seq brain atlas data displaying the main cell types. From this atlas we built an mRNA expression reference matrix defined for 1,243 marker genes and six cell types, from which we imputed a corresponding DNAm reference matrix over 110 marker genes (gene-promoter DNAm) and the same six cell types. The weight bar at the bottom indicates which marker genes are expected to be reliable. This DNAm reference matrix was then evaluated in single-nucleus DNAm (snmC-Seq2) data from the prefrontal cortex. **b**, Box plots displaying snmC-Seq2 DNAm data mapping to the TSS200 promoter region of specific brain cell subtype marker genes, comparing the DNAm values (*y* axis) between cells annotated to the corresponding cell type and cells annotated to other cell types (‘others’). We display box plots for six marker genes, including the neuronal marker *MYT1L*, the oligodendrocyte marker *ONDP1*, the astrocyte marker *SHR*, the OPC marker *RIN2*, the endothelial marker *SRGN* and the microglia marker *INPP5D*. The *P* values are derived from a one-tailed *t*-test. **c**, Box plot displaying *t*-statistics for each marker gene, derived by comparing scnmC-Seq2 values in the cell type the gene is a marker of, against all other cell types. Box plots are shown for each cell type, including only those marker genes for that given cell type. Red dashed line indicates the false discovery rate (FDR) = 0.05 significance level for individual *t*-statistics. *P* value derives from a one-tailed Wilcoxon rank-sum test, with the alternative hypothesis being that the *t*-statistics are significantly <0 (significant hypomethylation at the promoters of the marker genes in the corresponding cell types they define). **d**, Bar plots displaying estimated cell-type fractions for each cell type, obtained by applying our weighted robust partial correlations (wRPC) framework to the mean DNAm profile of each cell type, obtained by averaging over all single cells annotated to that cell type. **e**, Scatter-plots of the estimated neuron fractions of both reference matrices (*x* axis labels the ones derived from our DNAm-atlas using EpiSCORE, *y* axis labels the ones derived from the pseudo-bulk snmC-Seq2 data in FACS-sorted neuronal (Neu^+^) and non-neuronal (Neu^−^) populations (Gasparoni and Guintivano), as well as in bulk frontal cortex tissue (only Gasparoni). In Gasparoni we display both Alzheimer and control samples. **f**, We display the PCC and MAD between the snmC-Seq2 and EpiSCORE derived cell-type fractions for each brain cell type. In all box plots, the central bar denotes the median, the box width defines the IQR and whiskers extend to 1.5 × IQR in either direction. Source data

We next sought a more stringent validation at single-cell resolution. We collated a single-nucleus DNAm (snmC-seq2) dataset from the human prefrontal cortex^47^, encompassing 1,577 neurons, 1,157 oligodendrocytes, 435 astrocytes, 197 OPCs, 201 endothelial cells and 400 microglia, as annotated by the authors (Methods) and asked whether our DNAm reference matrix for brain (Fig. 3a) would be able to predict these cell types. From the snmC-seq2 data, we extracted the Bernoulli DNAm values for CpGs mapping to within 200 bp of the transcription start site (TSS200) of marker genes present in our DNAm reference matrix (Methods). The snmC-Seq2 data displayed very high sparsity, i.e. the resulting DNAm matrix defined over the TSS200 regions of 110 marker genes and 3,967 cells displayed over 90% missing values, not allowing us to directly apply our multivariate framework for estimating cell-type fractions to single nuclei. Instead, we adopted a univariate approach, performing a *t*-test for each of the 110 marker genes, comparing promoter DNAm for cells that ought to express the marker gene (as determined by the original scRNA-seq atlas for brain) against the cell types where the gene is not expressed. For 57 of the 110 markers, we observed a significant hypomethylation (FDR < 0.05) pattern in the cells where the marker gene is expressed compared to the cell types where it is not (Fig. 3b and Supplementary Fig. 16). For all cell types except neurons, the corresponding marker genes exhibited a clear trend toward promoter hypomethylation in that cell type (Fig. 3c). Notably, this trend was stronger for those marker genes for which we had assigned a higher EpiSCORE confidence score (Methods and Supplementary Fig. 17). As a second validation strategy, we estimated cell-type fractions in the pseudo-bulk profiles obtained by averaging the snmC-Seq2 DNAm profile of cells annotated to the same type (Methods). Annotated neurons, oligodendrocytes, astrocytes and microglia were correctly predicted to be these cell types (Fig. 3d). Direct comparison of our DNAm reference matrix to one defined by these pseudo-bulk profiles revealed a significantly low median absolute deviation (MAD = 0.11, Monte-Carlo randomization *P* < 0.0001) and a significantly high Pearson correlation (*P* = 0.56, *P* < 10^−15^; Supplementary Fig. 18). As a final validation, we used the snmC-Seq2 data to derive a new DNAm reference matrix (Methods), which we then applied to the same 450k DNAm Neu^+^ and Neu^−^ datasets considered earlier, to cross-compare obtained cell-type fractions with those derived with our DNAm reference matrix. Overall, we observed excellent agreement between the cell-type fractions obtained from the two separate DNAm reference matrices (Fig. 3e). Of note, comparison of the neuron reference profile in our DNAm reference matrix to the DNAm profile as given by the FACS-sorted Neu^+^ samples considered earlier, revealed an overall stronger correlation for the EpiSCORE DNAm reference profile than for the snmC-Seq2 derived one (Supplementary Fig. 19).

## Neuron-specific differential DNAm is enriched for SZ-risk loci

**Fig. 4:**
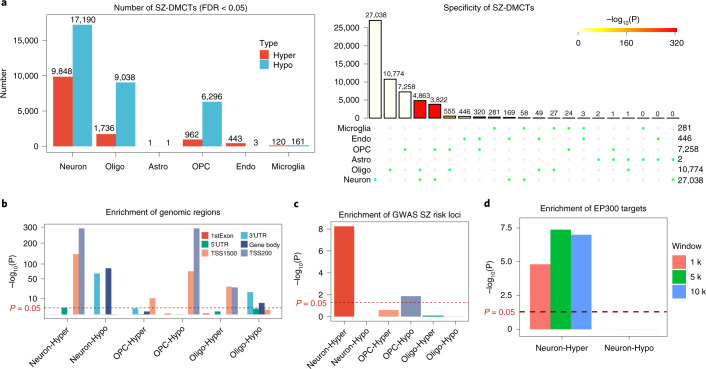
Application of the brain DNAm-atlas to an EWAS of schizophrenia. **a**, Bar plots display the number of cell-type-specific SZ-associated DMCTs, as inferred using CellDMC in an EWAS of SZ performed in the prefrontal cortex. Upset plot (right) displays the overlap of DMCTs between cell types. *P* value was computed from a one-tailed Fisher’s exact test of multiple-set intersections. **b**, Enrichment of regulatory regions among DMCTs displaying only those cell types (neurons, oligodendrocytes and OPCs) with sufficient numbers of DMCTs. *P* value was computed from a one-tailed Fisher’s exact test. **c**, Enrichment of SZ-associated GWAS loci among the DMCTs displayed in **f**. *P* values were estimated using a one-tailed Fisher’s test. **d**, Enrichment of EP300 ChIP-seq targets among neuron DMCTs and for three different choices of window size (1 kb, 5 kb, 10 kb). *P* values were estimated using a one-tailed Wilcoxon rank-sum test comparing binding intensity of neuron-DMCT genes against the binding intensity of genes not linked to DMCTs. Source data

To show how the DNAm-atlas can lead to new insight, we applied the brain DNAm reference matrix to an epigenome-wide association study (EWAS) conducted in the prefrontal cortex of 191 people with SZ and 240 controls^48^ (Methods and Supplementary Fig. 20a). Using the estimated cell-type fractions, we applied CellDMC^6^, an algorithm designed to detect cell-type-specific differential DNAm (DMCTs). Most SZ-associated DMCTs occurred in neurons, with lower but still significant numbers in oligodendrocytes and OPCs (Fig. 4a). Most of the neuron DMCTs were also specific to neurons and did not overlap with DMCTs in other cell types (Fig. 4a). We observed strong enrichment of promoter regions among hypermethylated neuron DMCTs and hypomethylated OPC DMCTs (Fig. 4b). Notably, only hypermethylated neuron DMCTs were strongly enriched for genome-wide association study (GWAS) SZ-risk loci (Methods and Fig. 4c), indicating a neuronal origin for SZ. Transcription factor (TF)-binding motif analysis revealed enrichment of IRF3 and EP300, two TFs that have been implicated in SZ risk^49–51^ (Supplementary Fig. 20b). We were able to validate these findings using chromatin immunoprecipitation (ChIP)-seq data for EP300 (Fig. 4d and Supplementary Fig. 20c). Given that EP300 expression is highly specific to neurons (Supplementary Fig. 20d,e), the observed enrichment of EP300 binding sites in hypermethylated neuron DMCTs, suggests reduced EP300 binding activity in neurons of individuals with SZ. Thus, these data illustrate how the DNAm-atlas can be combined with algorithms such as CellDMC to identify cell-type-specific differential DNAm.

## DNAm-atlas identifies cell of origin in pancreatic cancer

**Fig. 5:**
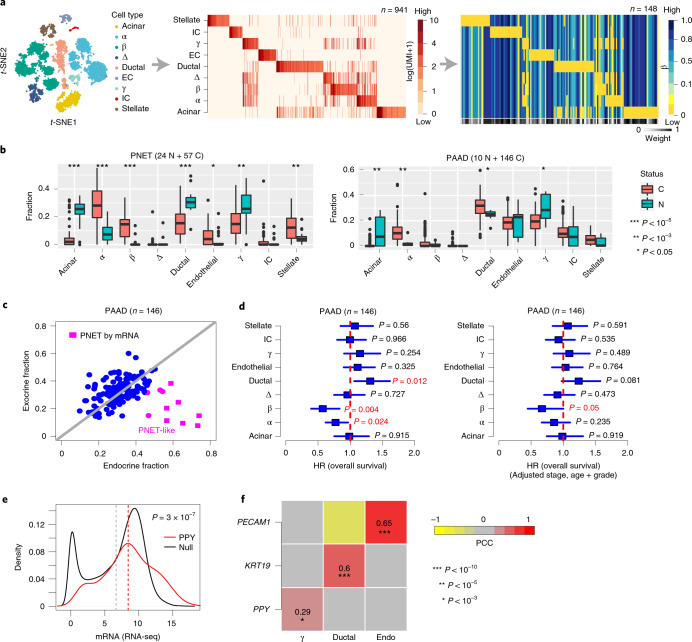
DNAm-atlas reveals cell of origin of pancreatic cancers and misdiagnosed cases. **a**, *t*-SNE diagram of pancreas scRNA-seq atlas, from which the mRNA expression reference and the imputed DNAm reference matrices are derived. The weight bar indicates the confidence level of the markers in the DNAm reference matrix. References are defined over nine cell types, as shown. **b**, Estimated cell-type fraction using the DNAm reference matrix in **a** in an Illumina 450k DNAm dataset of PNETs (C) and normals (N) and in the TCGA PAAD cohort. *P* values derive from a two-tailed Wilcoxon rank-sum test. **c**, Scatter-plot of the total exocrine (ductal and acinar) fraction (y axis) versus endocrine (α, β, γ and δ) fraction in the PAADs of TCGA. In pink we highlight samples that our DNAm-atlas predicts to be neuroendocrine in origin. Samples labeled as pink squares have been independently confirmed by mRNA expression to be PNETs. **d**, Hazard ratio (HR) forest plots derived from Cox regressions of overall survival versus estimated cell-type fractions in PAADs from TCGA. HRs, 95% CIs and *P* values are derived from univariate two-tailed Cox regressions (left) or multivariate Cox regressions (right), which included stage, age and grade as covariates. **e**, Density curves comparing mRNA expression level of *PPY*, a highly specific marker for pancreatic γ cells, across 146 PAAD tumors from TCGA, against the distribution of average gene expression across the same samples (‘null distribution’). The red dashed line indicates the mean expression for PPY, the gray dashed value is the mean of the null. The *P* value is derived from a one-tailed Wilcoxon rank-sum test, confirming that PPY displays higher than average gene expression. **f**, PCC heat map between the estimated cell-type fractions from the DNAm-atlas for the three most abundant cell types (ductal, endothelial and γ cells) and the corresponding mRNA expression levels of ductal (KRT19), endothelial (PECAM1) and γ-specific (PPY) markers. PCCs and one-tailed linear correlation test *P* values were derived from 144 PAAD samples with matched mRNA and DNAm data. In all box plots, the central bar denotes the median, the box width defines the IQR and whiskers extend to 1.5 × IQR in either direction. Source data

Next, we applied the DNAm-atlas to pancreatic cancer, which is often misdiagnosed^52^, to see whether it could identify the cell of origin and improve correct diagnosis. We derived and validated mRNA expression and DNAm reference matrices at the resolution of six cell types (Supplementary Figs. 6 and 21a)^53–55^. When applied to the pancreatic ductal adenocarcinomas (PAADs) from TCGA^56^ and a series of pancreatic neuroendocrine tumors (PNETs)^57^, we could correctly predict their respective ductal and endocrine origin (Supplementary Fig. 21b). These results were robust when we increased the cellular resolution of the DNAm reference matrix to nine cell types, now including four endocrine cell subtypes (α, β, γ and δ; Fig. 5a). Only α and β cells displayed an increase in PNETs compared to healthy samples, with γ cells displaying a corresponding decrease (Fig. 5b), thus indicating that PNETs arise from α or β cells, a result that is consistent with independent lines of evidence^58–62^. A scatter-plot of total exocrine versus endocrine fractions indicated a small number of PNET-like PAAD TCGA tumors (Fig. 5c), suggesting that these PAAD tumors have been misdiagnosed. Consistent with this, a recent study concluded that at least eight of the PAAD TCGA samples are PAAD-misdiagnosed PNET cases^52^, with seven of these profiled at the DNAm level and with all seven correctly predicted by our DNAm-atlas to be endocrine in origin (Fig. 5c). In addition, misdiagnosed PAAD cases displayed significantly better clinical outcome (Fig. 5d), consistent with PNET’s less-aggressive nature^52^. A surprising finding was the relatively high fraction of γ cells in PAAD tumors (Fig. 5b). However, consistent with this, the γ cell-specific marker PPY^63^ displayed significantly high expression in the TCGA PAAD samples (Fig. 5e), as well as in one PAAD scRNA-seq profile (Supplementary Fig. 22). For the three predicted most abundant cell types in PAAD samples (ductal, endothelial and γ cells), we observed good agreement between our DNAm-atlas derived cell-type fractions and the mRNA expression levels of KRT19 (a ductal marker), PECAM1 (an endothelial marker) and PPY (Fig. 5f).

## DNAm-atlas predicts new prognostic associations

**Fig. 6:**
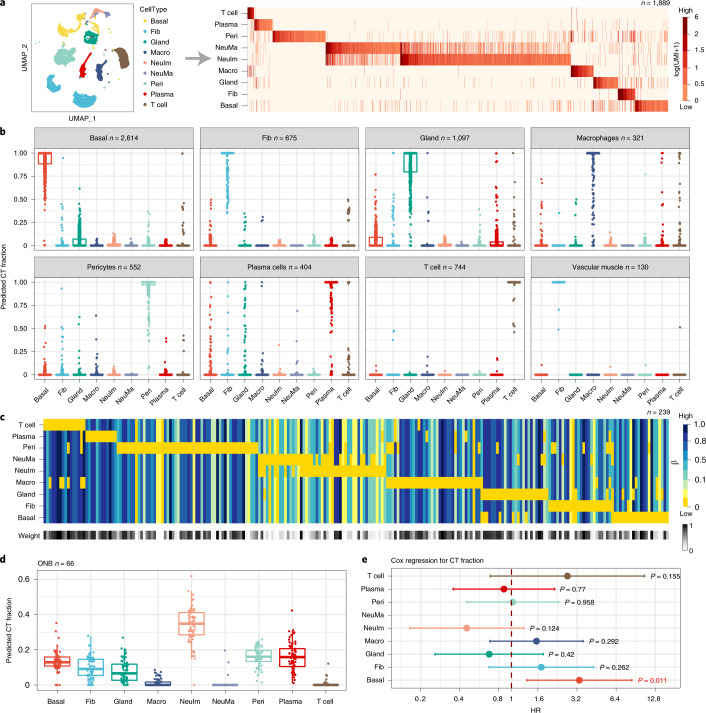
Validation and application of the olfactory epithelium DNAm-atlas to ONBs. **a**, *t*-SNE diagram displaying the main cell types in the OE and the associated mRNA expression reference matrix, all derived from the Durante et al. scRNA-seq dataset of two OE samples. The reference matrix is defined over 1,889 marker genes and nine cell types, including pericytes, endothelial cells, T cells, plasma cells, immature and mature neurons, fibroblasts, glandular and basal cells. **b**, Validation of the mRNA expression reference matrix in independent scRNA-seq data from two respiratory epithelium (RE) samples. Box plots display the estimated cell-type fractions (*y* axis) for all RE samples annotated to a given cell type. In all box plots, the central bar denotes the median, the box width defines the IQR and whiskers extend to 1.5 × IQR in either direction. **c**, The imputed DNAm reference matrix for OE, as derived using the EpiSCORE algorithm, defined over 239 marker genes and the same nine cell types. Weight bar indicates the reliability of the marker genes for inferring cell-type fractions. **d**, Application of the DNAm reference matrix in **c** to Illumina 450k DNAm data from 66 ONBs, with box plot displaying the estimated cell-type fractions in the ONBs. In all box plots, the central bar denotes the median, the box width defines the IQR and whiskers extend to 1.5 × IQR in either direction. **e**, HR forest plot derived by Cox regression of overall survival against the estimated cell-type fractions. 95% CIs and chi-squared test two-tailed *P* values are given (*n* = 66). Source data

We next applied our atlas to olfactory neuroblastomas (ONBs). A prevailing view is that ONBs derive from immature neurons in the olfactory epithelium (OE)^64^, yet this remains controversial with some studies suggesting distinct basal and neuronal subtypes^65^. We processed a scRNA-seq atlas of the OE^66^, to build an expression reference matrix encompassing 1,889 marker genes and nine cell types (mature and immature neurons, pericytes, macrophages, lymphocytes, plasma cells, fibroblasts, glandular and basal cells; Figs. 1c and 6a). We validated the mRNA expression reference matrix in independent scRNA-seq data from the respiratory epithelium (Fig. 6b) and imputed a corresponding DNAm reference matrix over 239 marker genes and the same nine cell types (Fig. 6c). Application to a bulk-tissue DNAm dataset of 66 ONBs^67^ (Methods), revealed a substantially higher fraction for the immature neuronal phenotype (Fig. 6d). However, ONBs also displayed variable basal and immune cell fractions, with the basal fraction correlating with poor clinical outcome (Fig. 6e). CpGs hypermethylated in samples with higher basal content were strongly enriched for a stemness signature defined at polycomb-repressive-complex-2 (PRC2) markers (Supplementary Tables 4 and 5). Thus, these findings confirm reports by Classe et al.^65^ of a poor outcome basal stem-like ONB subtype, but in contrast to Classe et al. and more consistent with existing literature^68–71^, we did not observe a positive correlation between T-cell infiltration and basal fraction (Supplementary Fig. 23)^68–71^.

In the context of the TCGA skin cutaneous melanoma DNAm dataset, estimated cell-type fractions correlated with overall survival; in primary melanoma, the fibroblast fraction correlated with poor clinical outcome, whereas in metastatic melanoma, the melanocyte and T-cell fractions were prognostic, with a high melanocyte low T-cell composition associating with poor outcome (Supplementary Fig. 24a), consistent with previous reports^72^. However, here we observed an association of low tumor-infiltrating lymphocyte numbers with poor outcome within stage 2 melanoma (Supplementary Fig. 24b), thus demonstrating how the atlas could be used to stratify patients in this challenging clinical subgroup.

## DNAm-atlas yields new insights in AD and BAV

Finally, we revisited the application to the human aorta, to see whether our atlas could provide insights into AD and bicuspid aortic valve (BAV), two cardiovascular diseases with dismal outcomes^73^. Applying the heart DNAm reference matrix to the same 450k DNAm dataset considered earlier, now including an additional six BAV cases^42^, revealed an increased macrophage, as well as lower fibroblast and endothelial fractions in BAV cases compared to age-matched healthy controls (Supplementary Fig. 24c)^43–46^. This may explain why BAV, a congenital condition, displays an association with aortic aneurysm and dissection^74^. We also predicted an increased fraction of smooth muscle cells (SMCs) in AD. Notably, AD has also been associated with a phenotypic SMC switch from a differentiated contractile phenotype into a highly proliferative de-differentiated one^75^. The observed increase in total SMC fraction could reflect this switch and therefore be associated with a loss of differentiated SMCs. Using independent DNAm markers for differentiated SMCs we were able to confirm a decrease in the differentiated SMC phenotype (Methods and Supplementary Fig. 24d).

## Discussion

As shown here across 13 tissue types, imputation of a tissue-specific DNAm reference matrix is possible from a corresponding tissue-specific scRNA-seq atlas. There are two main reasons why the imputation strategy works. First, a reasonable fraction (about 10–30%) of cell-type-specific marker genes exhibit a strong anti-correlative pattern between promoter DNAm and mRNA expression, thus allowing imputation of promoter DNAm levels from the observed gene expression level. Second, while this imputation procedure is imperfect for certain marker genes and cell types, the inference of cell-type fractions is very robust and can tolerate up to a 30–40% error rate in the DNAm reference matrix^6,17^. This robustness stems directly from the multivariate inference framework, which is tantamount to applying a voting algorithm for continuously valued variables, that is, as long as the majority of the imputed DNAm values in the reference matrix are approximately correct, the resulting inference of cell-type fractions should converge to a reasonably accurate solution.

As demonstrated here, the DNAm-atlas is a valuable resource that can provide biological insights of clinical importance, as well as helping to confirm previous but still controversial findings. For instance, the DNAm-atlas confirms the prevailing view that most ONBs derive from an immature neuronal phenotype, but also revealed a poor outcome subset characterized by a high basal fraction and stemness. This supports the view that there are different cells of origin for ONB. In the context of pancreatic cancer, our DNAm-atlas helps to establish α and β endocrine cells as the cells of origin of PNETs, while also identifying misdiagnosed PAAD cases. This highlights another potential use of our DNAm-atlas, to ascertain the diagnosis of specific cancer types. Highlighting broad applicability, the atlas also revealed insights in cardiovascular disease, demonstrating that both AD and BAV are characterized by an inflammation-induced degradation of the extracellular matrix and vasculature, while also highlighting a more-pronounced decrease of the endothelial fraction in the case of BAV. In combination with a cell-type-specific differential DNAm calling algorithm, we were able to confirm a neuronal origin for schizophrenia. The specific enrichment of SZ-risk GWAS loci among SZ-hypermethylated neuron DMCTs suggests that DNAm may play an important role in mediating the genetic risk of SZ. The enrichment for EP300 binding sites among hypermethylated neuron DMCTs is also noteworthy given that differential DNAm of EP300 has been related to prenatal stress exposure, in support of a neurodevelopmental origin for SZ^51^.

It is worth contrasting our strategy to build a DNAm-atlas with alternative ways to build tissue-specific DNAm references. For instance, one can in principle collate DNAm profiles for purified samples representing cell types within a given tissue, or generate such profiles using FACS sorting or laser capture microdissection techniques. However, for most tissue types these strategies are cumbersome, technically challenging and generally fail to achieve high-purity samples, which is critical for subsequent inference. DNAm profiles for specific cell types may also not be available, as for instance is the case for γ cells in pancreas^62^. By starting out from a scRNA-seq atlas and then imputing DNAm for all the cell types measured in the scRNA-seq assay, we can circumvent these major challenges. However, our imputation strategy also presents a number of limitations. One limitation relates to the ability of the scRNA-seq assay to measure all relevant cell types at sufficient read depth and in sufficient numbers to facilitate identifying as many cell-type-specific expression markers as possible. As we have seen, this limitation is particularly evident in the case of a multi-tissue atlas such as HCL^39^. The second limitation is that the imputation is only possible for a relatively small fraction (10–30%) of marker genes. This can lead to low numbers of marker genes and difficulties to distinguish closely related cell types, such as endocrine or epithelial subtypes in pancreas or kidney, respectively. Indeed, the relatively high γ-cell fraction in PAADs may indicate residual confusion with the more common endocrine fractions. To address this challenge will require future improvements that depart from the promoter-centric imputation approach implemented in EpiSCORE to incorporate CpGs that map to other regulatory elements such as enhancers. Given recent improvements in mapping cell-type-specific enhancer–promoter interactions^76,77^, this is likely to be a promising strategy. For the current version of the DNAm-atlas and to help users assess the reliability of each DNAm reference matrix, we provide a summary table ranking tissues by the quality and extent of validation (Supplementary Table 6). Finally, it is worth stressing that, as with cell-type deconvolution of bulk RNA-seq^78^, estimated cell-type fractions from our DNAm-atlas should be interpreted more as relative fractions, that is for a given cell-type, fractions are comparable across samples, which is the main requirement to justify their subsequent use in linear regression models.

In summary, the DNAm-atlas is a unique resource enabled by open-access data and scalable to all human tissues and organs, which will be of great value for a wide range of problems including cancer diagnosis, identification of cell-type-specific biomarkers and more generally to significantly improve the biological and clinical interpretation of large-scale bulk-tissue DNA methylome studies.

## Methods

All scRNA-seq datasets used for construction and validation of tissue-specific mRNA expression references are listed in Supplementary Table 1. The detailed descriptions of these scRNA-seq datasets and how they were processed are provided in Supplementary Information.

### Imputation of DNAm with EpiSCORE

EpiSCORE is described in detail elsewhere^22^. Briefly, EpiSCORE first builds an expression reference matrix from an scRNA-seq dataset where cells have been clustered and annotated to specific cell types. In building this expression reference matrix, a key factor to consider is cell-type resolution. For example, there are many different types of lymphocytes, but one may wish to treat them all as one generic lymphocyte. Having decided on the main cell types of interest (assume this number is *K*), we next perform Wilcoxon rank-sum tests to identify marker genes for each cell type. Ideal marker genes are those for which the median expression in the other *K* − 1 cell types is zero. These marker genes attain a maximum marker specificity score (MSS) of *K* − 1. However, if the number of resulting marker genes is too low (we recommend at least 100 marker genes), then the MSS threshold can be relaxed. The expression reference matrix is obtained by taking the median over all cells of a given cell type. Subsequent imputation of DNAm levels is only performed for the subset of marker genes for which promoter DNAm and gene expression are anticorrelated, as determined from two independent datasets with matched DNAm and mRNA expression data (see subsection below). As high expression is generally associated with low or near-zero promoter DNAm levels^79^, for these entries we imputed a promoter DNAm value of zero. For genes that are not expressed, silencing could be associated with other factors such as repressive histone marks, hence for these zero entries we imputed promoter DNAm values using a two-state γ mixture model^80^, as implemented with gammamixEM from the mixtools R package^81^. Marker genes are then weighted according to the imputed DNAm value in the cell types where that gene is not expressed. For informative marker genes, this weight is closer to 1. The imputed DNAm reference matrix obtained in previous step is then used to estimate corresponding cell-type fractions in a bulk-tissue DNAm profile using a wRPC procedure, with weights as defined above. For the bulk-tissue DNAm samples, promoter DNAm levels are assigned by taking the average DNAm of CpGs within 200 bp upstream of the TSS, or if not available, by taking the average DNAm over first Exon CpGs, following our FEM algorithm^82^. The multivariate model is then run using Huber’s robust M-estimator^83^. As cell-type fractions need to be non-negative and add to 1, we set any estimated negative regression coefficients to zero and scale the rest so that their sum equals unity^84,85^.

### Matched DNAm mRNA expression datasets

To identify ‘imputable’ genes we made use of two separate databases of matched DNAm and mRNA expression data: the Stem-Cell-Matrix Compendium-2 (SCM2)^86–88^, available from the Gene Expression Omnibus (GEO) under accession code GSE30654, and a sequencing-based database derived from the Epigenomics Roadmap (RMAP)^79,89^.

### Validation of the mRNA expression reference matrices

The specific scRNA-seq expression datasets used for validation for each tissue-type are described in Supplementary Information. Here we briefly describe the overall strategy. For a given mRNA expression reference matrix defined over a given number of marker genes and cell types, we used robust partial correlations^85^ to estimate corresponding cell-type fractions in each single cell from the validation scRNA-seq dataset.

### Validation of the DNA methylation reference matrices

#### DNAm-atlas-derived tumor purity and total immune cell scores

One way to systematically validate the tissue-specific DNAm reference matrices is by application to Illumina 450k DNAm dataset from corresponding cancer types from TCGA to obtain tumor purity and total immune cell scores. For a given tissue-specific DNAm reference matrix we identified the cell types that define the tumor stroma (typically, this includes all immune cells, endothelial cells and fibroblasts) and for these cell types the estimated cell-type fractions were added and subtracted from 1 to define the DNAm-atlas tumor purity index. In the case of the total immune cell score, we added the estimated fractions of immune cells. The DNAm-atlas-based tumor purity estimate was then benchmarked against variety of different methods, including the gene expression-based ESTIMATE algorithm^24^, CNV-based ABSOLUTE^25^, IHC and a method combining all three (CPE)^26^. In the case of the total immune cell score, we benchmarked this against the gene expression-based (ESTIMATE)^24^ and DNAm-based (LUMP)^26^ immune cell scores.

#### Knowledge-based validation

The systematic validation in TCGA only validates the tissue-specific DNAm reference matrices at a coarse cellular resolution. To validate the DNAm reference matrices at a higher cellular resolution is difficult in the absence of high-quality single-cell methylomics data. However, for certain tissue types, validations in bulk tissue are possible using known biology. For instance, in the case of skin, the epidermis is known to be dominated by keratinocytes, whereas the dermis is rich in fibroblasts and contains few keratinocytes. In the context of cancer, it is possible to validate the DNAm reference in terms of their predictions as to their cell of origin (such as melanocytes for melanoma, cholangiocytes for cholangiocarcinoma and hepatocytes for hepatocellular carcinoma).

### Validation DNAm datasets

#### TCGA

We downloaded and processed level-3 Illumina 450k DNAm data from the TCGA data portal for the following cancer types LUAD, LUSC, BLCA, LIHC, CHOL, SKCM, PAAD, KICH, KIRC, KIRP, PRAD, BRCA, COAD, READ, GBM, LGG and ESCA datasets, as described by us previously^90^. Briefly, we removed probes with >30% missing values. Remaining missing values were imputed using the impute.knn (*k* = 5) function from impute R package^91^. Type-2 probe bias was adjusted using BMIQ^92^. For liver, GSE123995 is an EPIC dataset with 56 hepatocyte samples. Raw idat files were processed by minfi. Probes with *P* value >0.01 were assigned a missing value and any probe with >25% missing values were removed. The rest of missing values were imputed with knn (*k* = 5). Type-2 probe bias was adjusted with BMIQ. GSE49656 is a 450k dataset with 32 cholangiocarcinoma and four healthy liver samples. The β matrix was downloaded from GEO. Probes with >25% missing values were removed and the rest of missing values imputed with impute.knn (*k* = 5). Then the β matrix was normalized with BMIQ.

#### Pancreas

GSE143209 is a 450k dataset of 64 human Langerhans islet samples (bulk tissue). The raw idat files were loaded with minfi. Values with *P* value of detection >0.01 compared to negative control probes were consider low quality. Probes with >25% low-quality values across samples were deleted. The remaining low-quality values were imputed with impute.knn (*k* = 5). Finally, we applied BMIQ. GSE124809 is another human bulk islet 450k dataset (only three samples). The β and *P* value matrices are provided on GEO. We removed probes with any missing values, followed by BMIQ normalization. GSE122126 is a DNAm-atlas containing purified pancreatic cell samples (three acinar cell samples, three β cell samples and four ductal cell samples), generated with both 450k and EPIC data. Methylation array idat files were processed with the minfi R package. Values with detection *P* values >0.01 were assigned as NA. We then removed probes with missing values in >25% samples and imputed the rest with impute.knn (*k* = 5). Finally, we applied BMIQ.

#### Brain snmC-seq2

This is a single-nucleus methylcytosine sequencing-2 (snmC-seq2) dataset from human prefrontal cortex, consisting of 4,238 nuclei (3,967 after quality control)^47^. Processed data were downloaded from GEO (GSE130711). The cell-type annotation was provided by the authors, which included 1,577 neurons, 1,157 oligo, 435 astro, 197 OPC, 201 endo and 400 microglia. We only kept CpGs with total read of 1 in each nucleus to exclude mitochondrial cytosines. Then we mapped CpGs to within TSS200 of 110 marker genes in the DNAm reference matrix, keeping only those CpGs with reads in at least five nuclei. This resulted in a total of 1,119 CpGs mapping to 103 marker genes. For each CpG, we thus obtained a Bernoulli DNAm value (which is exclusively 0 or 1). Finally, we averaged the Bernoulli DNAm values for CpGs mapping to the TSS200 of each marker gene.

#### Brain Illumina DNAm datasets

The Guintivano et al. dataset^36^ is an Illumina Human 450k Methylation dataset of 58 flow-sorted dorsolateral prefrontal cortex samples (29 purified neurons and 29 purified glia) from non-psychiatric controls, with raw data available from FlowSorted.DLPFC.450k Bioconductor package. Gasparoni et al.^37^ (GSE66351) is an Illumina 450k DNAm dataset of both bulk and cell-sorted postmortem frontal cortex samples from a study of Alzheimer’s disease. In total, there were 31 sorted neuronal (16 controls and 15 disease), 31 non-neuronal (16 controls and 15 disease) and 128 bulk samples (52 controls and 76 disease). For both datasets, raw data was processed with minfi. Probes with >25% NAs (defined by *P* > 0.01) were discarded. The remaining NAs were imputed with impute.knn (*k* = 5), followed by BMIQ normalization. Pai et al.^38^ is an EPIC (850k) DNAm dataset of 100 sorted neuronal samples derived from postmortem frontal cortex of people with SZ (*n* = 29), bipolar disorder (*n* = 28) and controls (*n* = 26). Raw idat files were downloaded from GEO (GSE112179) and processed with minfi. We only kept probes with non-missing data and subsequently data were normalized with BMIQ.

#### Skin datasets

The human dermis (*n* = 40) and epidermis (*n* = 38) 450k dataset^27^ is available from GEO (GSE51954). We processed the idat files with minfi^93^, impute and BMIQ using a similar procedure described for the other datasets. In addition, we downloaded the EPIC 850k dataset from Sarkar et al.^30^ from GEO (GSE142439), which contains eight skin fibroblast samples. Data were normalized with minfi and BMIQ, as described for the other datasets.

#### Human aorta

This 450k DNAm dataset is available from GEO (GSE84274) and contains 12 AD, 6 BAV and 6 age-matched healthy controls. We processed the idat files with minfi^93^, impute^91^ and BMIQ^92^ using a similar procedure as described for the other datasets.

#### Pancreatic neuroendocrine tumors

This Illumina 450k dataset derives from Pipinikas et al.^57^ and consists of 24 healthy (exocrine and endocrine) samples, 4 healthy livers, 45 primary PNETs and 12 liver metastases (after quality control). Processing of the idat files and quality control was performed with minfi, impute and BMIQ as described for the other datasets.

#### Olfactory neuroblastomas

This is an Illumina 450k dataset from Capper et al.^67^ that contains 66 ONB samples. Raw idat files were processed with minfi. Probes with >25% NAs (defined by *P* > 0.01) were discarded. The remaining NAs were imputed with impute.knn (*k* = 5), followed by BMIQ normalization.

### Derivation of DNAm reference matrix for brain from snmC-seq2 data

We used the same processed snmC-Seq2 data from Lee et al.^47^ (as described earlier) to build a new DNAm reference matrix. This time we derived a DNAm data matrix defined over the promoters of 23,056 genes by averaging the Bernoulli DNAm values of CpGs mapping to within TSS200. This resulted in a DNAm data matrix over 23,056 gene-promoters and 3,967 nuclei (1,577 neurons, 1,157 oligo, 435 astro, 197 OPC, 201 endo and 400 microglia). We then selected marker genes for a given cell type using a Wilcoxon rank-sum test (FDR < 0.05) comparing the DNAm values in that given cell type (we required at least ten non-missing values in the given cell type) to all other cell types. For a given cell type, the selected marker genes were ranked by the area under the curve (AUC), where an AUC value close to 1 means significantly lower DNAm in that cell type compared to all others. In effect, this ranking procedure selects marker genes of a cell type as those with unmethylated promoters, which is permissive of the marker genes being highly expressed in that cell type. For each cell type we selected the top-20-ranked marker genes to ensure at least 100 marker genes in total and because for the top-20 the minimum AUC value was always 0.8 or higher (for most cell types, the minimum AUC value of the 20th ranked genes was >0.9). The total number of unique marker genes across all six cell types was 119. The final DNAm reference matrix of 119 marker genes and six cell types was obtained by averaging the DNAm levels of each gene over all cells within a cell type.

### Identification of cell-type-specific schizophrenia-associated differential DNAm

#### Schizophrenia EWAS dataset

We analyzed an Illumina 450k DNAm dataset of prefrontal cortex from 335 non-psychiatric controls and 191 patients with SZ published in Jaffe et al.^48^. Raw idat files were downloaded from GEO (GSE74193). Illumina definition of β-value was used. Probes with >25% failed samples defined by *P* > 0.01 comparing to negative controls were discarded. The remaining NAs were imputed with impute R package using impute.knn (*k* = 5). CpGs on chromosomes X and Y were also removed, resulting in 473,536 probes. Type-2 probe bias was corrected with BMIQ. We only kept samples with BestQC = true and DropSample = false as recommended in Jaffe et al. Following Jaffe et al., we also restricted to samples with age >16, which resulted in 191 SZ and 240 control samples.

#### Identification of DMCTs with CellDMC

We first performed Singular Value Decomposition (SVD) on the normalized DNAm data matrix to assess the major sources of variation. The strongest sources of variation were cell-type fraction, followed by slide and age. CellDMC was run to identify cell-type-specific differentially methylated cells between controls (*y* = 0) and SZ (*y* = 1), using the following linear model with interaction terms

$$\overrightarrow {\beta _c} = \mathop {\sum}\limits_{k = 1}^6 {\mu _{ck}} \overrightarrow {\widehat {f_k}} + \mathop {\sum }\limits_{k = 1}^6 \beta _{ck}^{(I)}\overrightarrow {\widehat {f_k}} \ast \mathop{y}\limits^{\rightharpoonup} + \gamma \overrightarrow {Age} + \rho \overrightarrow {Slide} + \vec \varepsilon$$

where $\overrightarrow {\beta _c}$ is the DNAm β value vector for cytosine *c*, $\overrightarrow {\widehat {f_k}}$ are the estimated brain cell-type fractions using our DNAm brain atlas and where $\vec \varepsilon$ is an independent and identically distributed Gaussian error term. The significance threshold for calling DMCTs was FDR < 0.05.

#### Enrichment of GWAS SZ-risk loci

We obtained a list of 145 SZ GWAS loci from Pardinas et al.^94^. DMCTs were categorized depending on cell type and directionality of DNAm change and for each category we counted the number of DMCTs falling within a SZ GWAS locus. Statistical significance was assessed using a one-tailed Fisher’s exact test to test for overenrichment.

#### Enrichment of TF-binding motifs

For each category of DMCT, we selected the 250 most-significant DMCTs mapping to the TSS200 region of genes. This gene list was then used as input to the cisTarget function of the RcisTarget R package^95^. We ran this function with the database hg19-500bp-upstream-7species.mc9nr.feather, which contains the motif rankings for regions 500 bp upstream of the TSS of 22,284 genes across seven species and is available online (https://resources.aertslab.org/cistarget/). The motif annotation database used is motifAnnotations_hgnc, which is available in the package. We extracted the TFs with high confidence in the resulted table including direct annotation and homologous genes and found the overlap with SZ GWAS loci genes.

#### Enrichment of ChIP-seq targets

We downloaded the EP300 binding targets and binding intensity values from the ChIP-seq atlas^96^ http://chip-atlas.org/ for all three choices of window size ±1 kb, 5 kb and 10 kb centered on the TSS of a gene. We then compared the binding intensity values for genes associated with neuron hypermethylated DMCTs to those of genes not associated with any DMCT, using a one-tailed Wilcoxon rank-sum test to assess statistical significance. This analysis was performed in two ways: by focusing only on the binding intensity values within fetal brain and by averaging the binding intensity values across all available samples with EP300 ChIP-seq data.

### Dissection of smooth muscle cell phenotypes in human aorta

We devised an independent algorithm, based on ideas from HEpiDISH^17^, to obtain relative SMC-D (SMC differentiated) and SMC-P (SMC proliferative) fractions. First, we used a Wilcoxon rank-sum test to identify differentially expressed genes between SMC-D and SMC-P cells in the heart scRNA-seq dataset. Genes with FDR < 0.05 and with median expression level above 0 in one cell type and median expression level of 0 in the other were chosen. This resulted in 350 SMC-D and 3 SMC-P marker genes. Next, we selected marker genes displaying low expression and consistent promoter hypermethylation (*β* > 0.6) in at least three of the other four heart cell types, which includes cardiomyocytes, endothelial cells, fibroblasts and immune cells. This requirement helps to ensure that the SMC-D and SMC-P fractions to be estimated are not confounded by the presence of the other cell types. To assess promoter DNAm levels, we used ENCODE cell lines HCM (cardiac myocytes), HUVEC (umbilical vein endothelial cells) and HCF (cardiac fibroblasts) and the immune cell samples from Reinius et al. For the 60 immune cell samples from Reinius et al., we used the median DNAm level across all samples. In total, we identified five marker genes for SMC-D cells (*RERGL*, *CASQ2*, *UTRN*, *SORBS2* and *PTP4A3*), with their average promoter DNAm level (TSS200 region) representing a proxy for the relative SMC-P fraction.

### DNAm-atlas resource

The DNAm-atlas, including all the mRNA and DNAm reference matrices for the 13 tissue types, is published as a resource on figshare (https://figshare.com/projects/EpiSCORE-atlas_version-1_/111473).

### Reporting Summary

Further information on research design is available in the Nature Research Reporting Summary linked to this article.

## Online content

Any methods, additional references, Nature Research reporting summaries, source data, extended data, supplementary information, acknowledgements, peer review information; details of author contributions and competing interests; and statements of data and code availability are available at 10.1038/s41592-022-01412-7.

## Supplementary information

Supplementary Information Supplementary Figs. 1–24 and Supplementary Methods.

Reporting Summary

Peer Review Information

Supplementary Tables Supplementary Tables 1–6.

Supplementary Data Readme file and example R-script+ data to run example.

## Data Availability

The DNAm datasets analyzed in this manuscript are all publicly available from the respective publications or from GEO (www.ncbi.nlm.nih.gov/geo/) under the following accession nos.: GSE123995 (hepatocytes), GSE49656 (cholangiocarcinoma), GSE122126, GSE143209 and GSE124809 (pancreatic cell types), GSE84274 (human aorta samples), GSE130711 (human prefrontal cortex snmC-seq2), GSE66351, GSE112179 (human frontal cortex 450k and EPIC) and GSE51954 (human dermis and epidermis). The human purified dorsolateral prefrontal cortex 450k dataset from Guintivano et al. is available from FlowSorted.DLPFC.450k Bioconductor package. TCGA Illumina 450k datasets for LUAD, LUSC, BLCA, LIHC, CHOL, SKCM, PAAD, KICH, KIRC, KIRP, PRAD, BRCA, COAD, READ, GBM, LGG and ESCA datasets are available from the GDC data portal https://portal.gdc.cancer.gov/. The data file hg19-500bp-upstream-7species.mc9nr.feather, which contains the motif rankings for regions 500 bp upstream of the TSS of 22,284 genes across seven species is available online from https://resources.aertslab.org/cistarget/. Source data for each main figure have been provided in Excel spreadsheets labeled with their respective figure number. All source data Excel files are in the zip file labeled ‘SourceData.zip’. Source data are provided with this paper.

## Source data

Source Data Fig. 1 Summary statistics source data.
Source Data Fig. 2 Summary statistics source data.
Source Data Fig. 3 Summary statistics source data.
Source Data Fig. 4 Summary statistics source data.
Source Data Fig. 5 Summary statistics source data.
Source Data Fig. 6 Summary statistics source data.

## Code availability

EpiSCORE^97,98^ is freely available as an R package from figshare (https://figshare.com/articles/software/EpiSCORE_R_package/14401340) and from GitHub (https://github.com/aet21/EpiSCORE) under a GPL-2 license. The R package comes with a vignette and tutorial, sample datasets and a reference manual. A capsule demonstrating a simple use of the DNAm-atlas reproducing some of the results for liver tissue is available on OceanCode (http://codeocean.com) via https://codeocean.com/capsule/2549317/tree/v3 or 10.24433/CO.8741744.v3. We also provide brief pseudocode and source data for running an application of the DNAm-atlas in skin, which can be found in Supplementary Data File 1.
