# Supplementary material for: A pan-tissue DNA methylation atlas enables in silico decomposition of human tissue methylomes at cell-type resolution
Source: Nat Methods. 2022 Mar 11;19(3):296–306. doi: 10.1038/s41592-022-01412-7 (PMC8916958; doi:10.1038/s41592-022-01412-7)
Supplement: Supplementary file 1 — Supplementary Figs. 1–24 and Supplementary Methods. [file 41592_2022_1412_MOESM1_ESM.pdf]

nature portfolio

https://doi.org/10.1038/s41592-022-01412-7

Supplementary information

A pan-tissue DNA methylation atlas enables in silico decomposition of human tissue methylomes at cell-type resolution

In the format provided by the authors and unedited

Supplementary information

Supplementary Figures

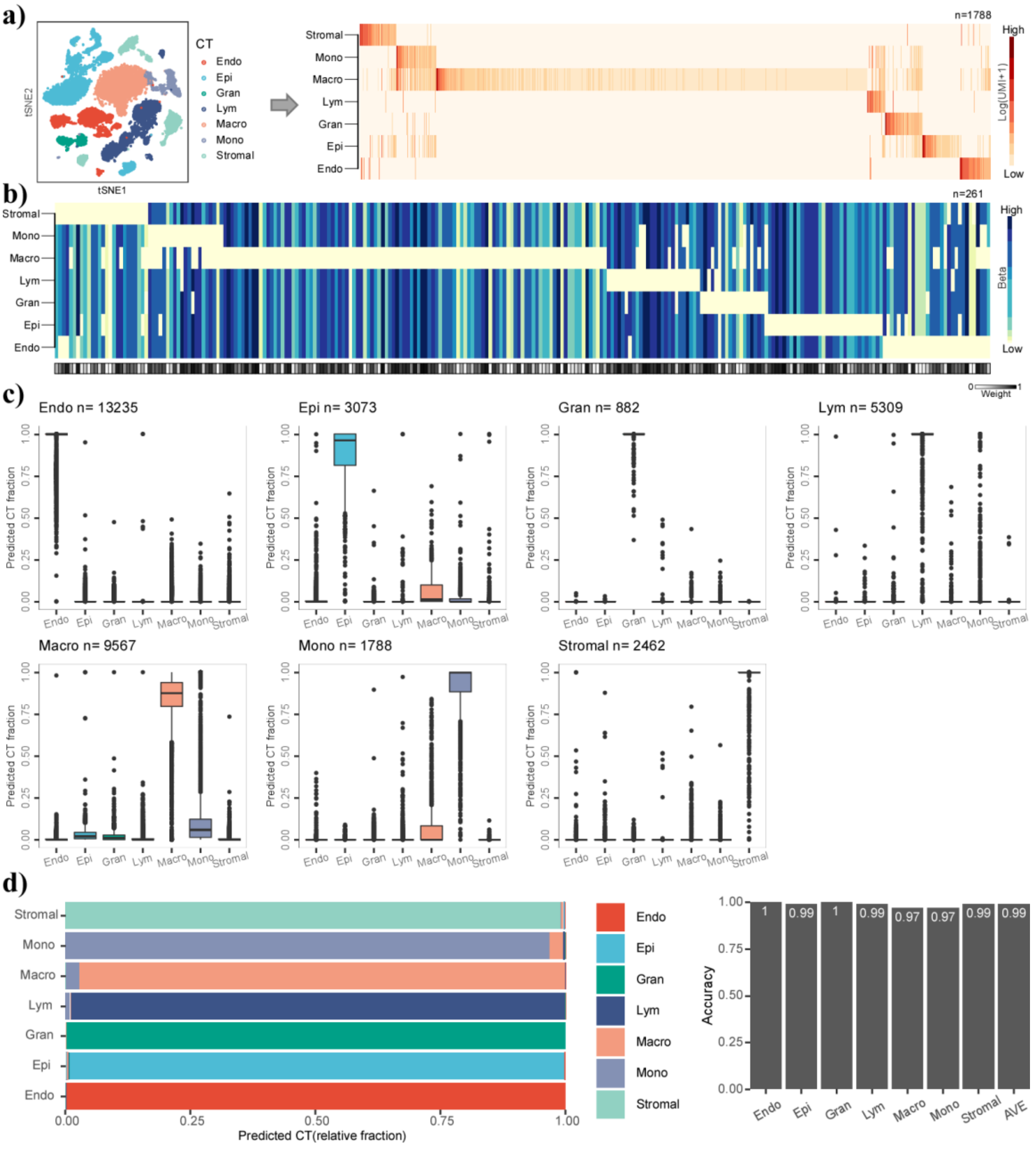

SI fig.S1: Construction and validation of the lung mRNA expression reference matrix. a) tSNE diagram of lung scRNA-Seq atlas and the scRNA-seq expression reference matrix over 7 main lung cell subtypes and 1788 marker genes, constructed from Travaglini et al's 10x scRNA-Seq data (patient 3 sample). **b)** Heatmap displaying the imputed DNAm reference matrix for 261 marker genes. **c)** Boxplots of estimated cell-type fractions for each single cell from Travaglini et al's scRNA-seq data (patient 1&2 samples). The number of single cells of each lung cell type is given. Cell-type fractions were estimated using RPC with the scRNA-Seq expression reference as given in a). **d)** Left: Barplot displaying the relative fractions (x-axis) of cells of a given cell-type (labeled on y axis) that were predicted to be endothelial cells (EC), epithelial cells (Epi), granulocytes (Gran), lymphocytes (Lym), macrophages (Macro),

monocytes (Mono) and stromal cells (Stromal), based on the cell-type fraction estimates obtained in c). Right: Barplot displaying the overall accuracy of the classification. In all boxplots, the central bar denotes the median, the box-width defines the IQR and whiskers extend to 1.5 IQR in either direction.

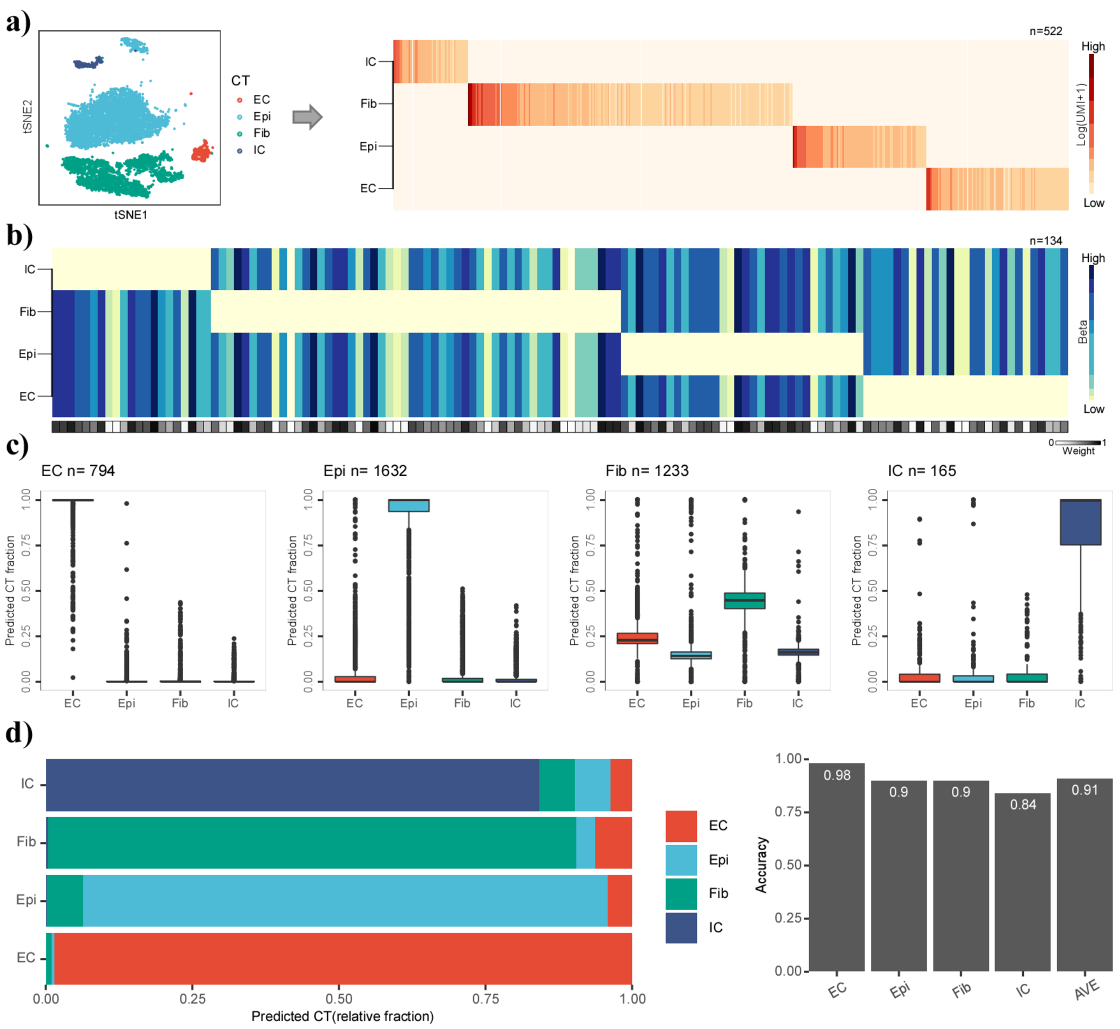

SI fig.S2: Construction and validation of the bladder mRNA expression reference matrix. **a)** tSNE diagram and the scRNA-seq expression reference matrix over 4 main bladder cell subtypes and 522 marker genes, constructed from Yu et al's 10x scRNA-Seq data. **b)** Heatmap displaying the imputed DNAm reference matrix for 134 marker genes. **c)** Boxplots of estimated cell-type fractions for each single cell from HCL Microwell-Seq bladder dataset. The number of single cells of each bladder cell type is given. Cell-type fractions were estimated using RPC with the scRNA-Seq expression reference as given in a). **d)** Left: Barplot displaying the relative fractions (x-axis) of cells of a given cell-type (labeled on y axis) that were predicted to be endothelial cells (EC), epithelial cells (Epi), fibroblasts (Fib) and immune cells (IC), based on the cell-type fraction estimates obtained in c). Right: Barplot displaying the overall accuracy of the classification. In all boxplots, the central bar denotes the median, the box-width defines the IQR and whiskers extend to 1.5 IQR in either direction.

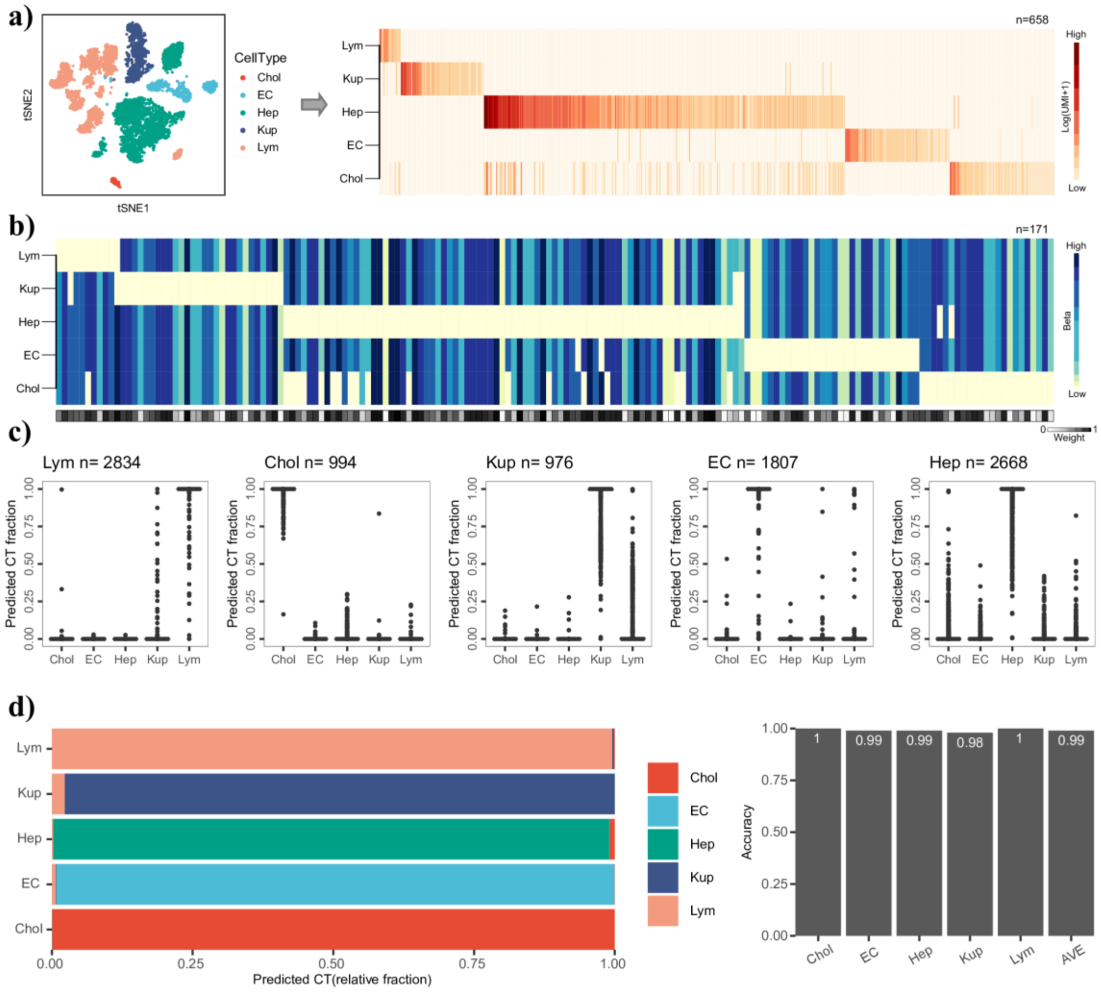

SI fig.S3: Construction and validation of the liver mRNA expression reference matrix. a) tSNE diagram and the scRNA-seq expression reference matrix over 5 main liver cell subtypes and 658 marker genes, constructed from MacParland et al's 10x scRNA-Seq data. **b)** Heatmap displaying the imputed DNAm reference matrix for 171 marker genes. **c)** Boxplots of estimated cell-type fractions for each single cell from HCA mCEL-Seq2 liver dataset. The number of single cells of each liver cell type is given. Cell-type fractions were estimated using RPC with the scRNA-Seq expression reference as given in a). **d)** Left: Barplot displaying the relative fractions (x-axis) of cells of a given cell-type (labeled on y axis) that were predicted to be cholangiocytes (Chol), endothelial cells (EC), hepatocytes (Hep), immune cells (IC) and kupffer cells (Kup), based on the cell-type fraction estimates obtained in c). Right: Barplot displaying the overall accuracy of the classification. In all boxplots, the central bar denotes the median, the box-width defines the IQR and whiskers extend to 1.5 IQR in either direction.

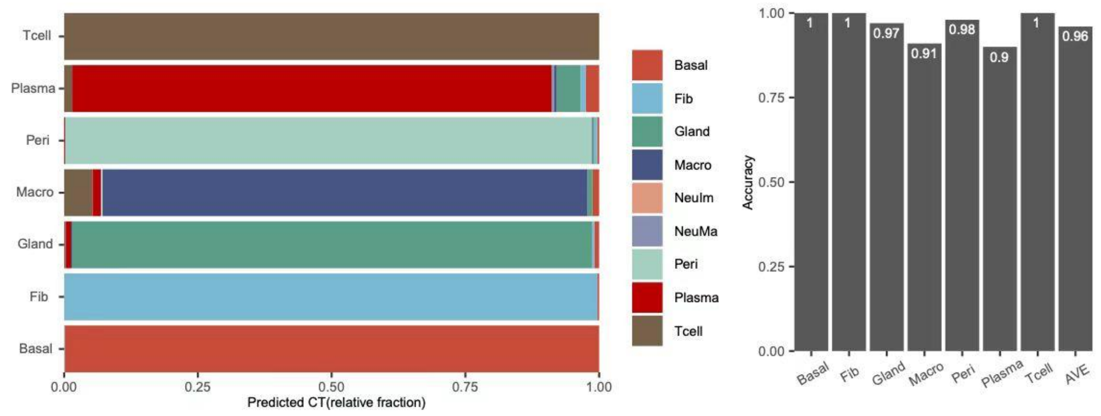

SI fig.S4: Prediction accuracy of olfactory epithelium scRNA-seq reference matrix. Left: Barplot displaying the relative fractions (x-axis) of cells of a given cell-type (labeled on y axis) that were predicted to be basal cells (Basal), fibroblasts (Fib), gland cells (Gland), macrophages (Macro), immature neurons (NeuIm), mature neurons (NeuMa), pericytes (Peri), plasma cells (Plasma) and T cells (Tcell), based on the cell-type fraction estimates obtained in Figure 4b. Right: Barplot displaying the overall accuracy of the classification.

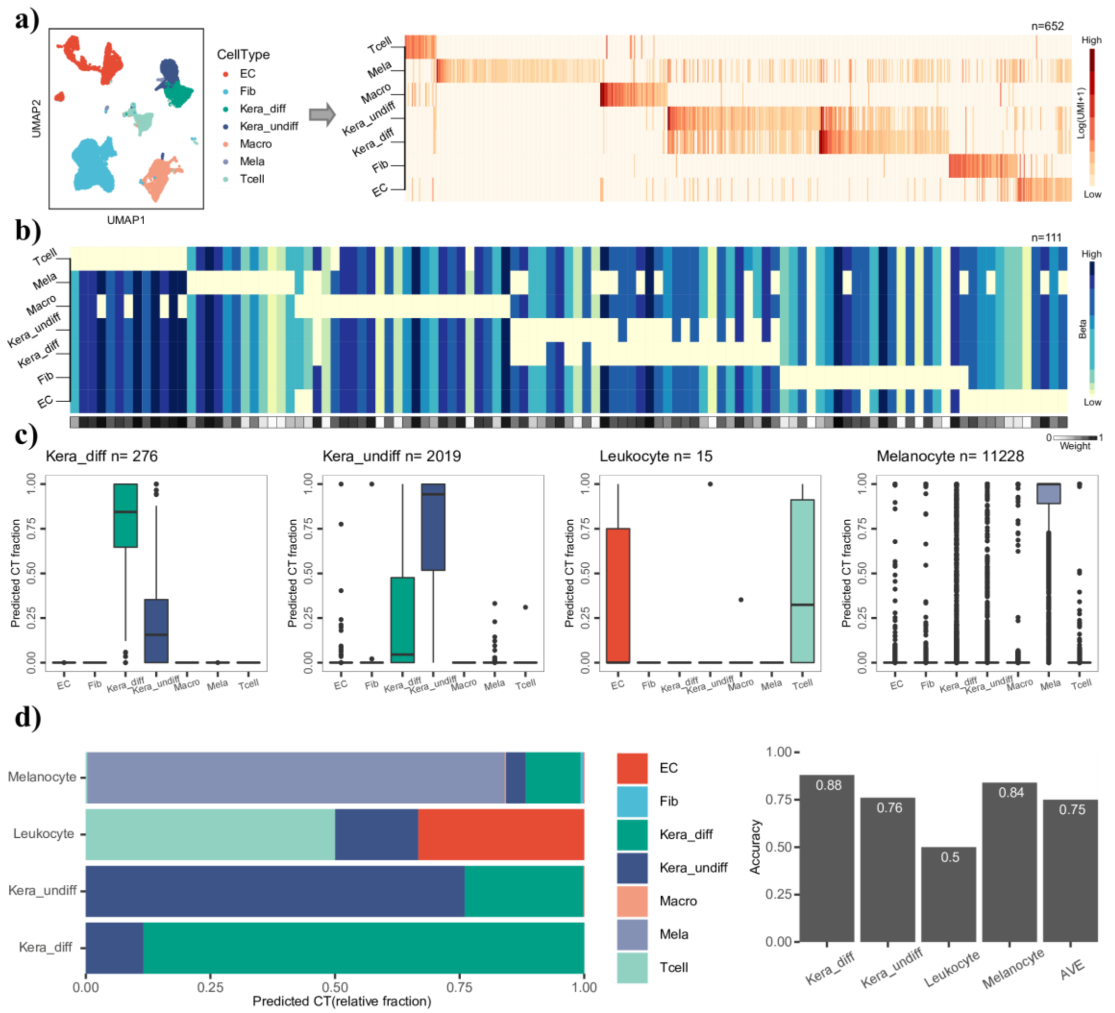

SI fig.S5: Construction and validation of the skin mRNA expression reference matrix. a) tSNE diagram and the scRNA-seq expression reference matrix over 7 main skin cell subtypes and 652 marker genes, constructed from Solé-Boldo et al's 10x scRNA-Seq data. **b)** Heatmap displaying the imputed DNAm reference matrix for 111 marker genes. **c)** Boxplots of estimated cell-type fractions for each single cell from MCA1 SS2 skin dataset. The number of single cells of each skin cell type is given. Cell-type fractions were estimated using RPC with the scRNA-Seq expression reference as given in a). **d)** Left: Barplot displaying the relative fractions (x-axis) of cells of a given cell-type (labeled on y axis) that were predicted to be endothelial cells (EC), fibroblasts (Fib), differentiated keratinocytes (Kera_diff), undifferentiated keratinocytes (Kera_undiff), macrophages (Macro), melanocytes (Mela) and T cells (Tcell), based on the cell-type fraction estimates obtained in c). Right: Barplot displaying the overall accuracy of the classification. In all boxplots, the central bar denotes the median, the box-width defines the IQR and whiskers extend to 1.5 IQR in either direction.

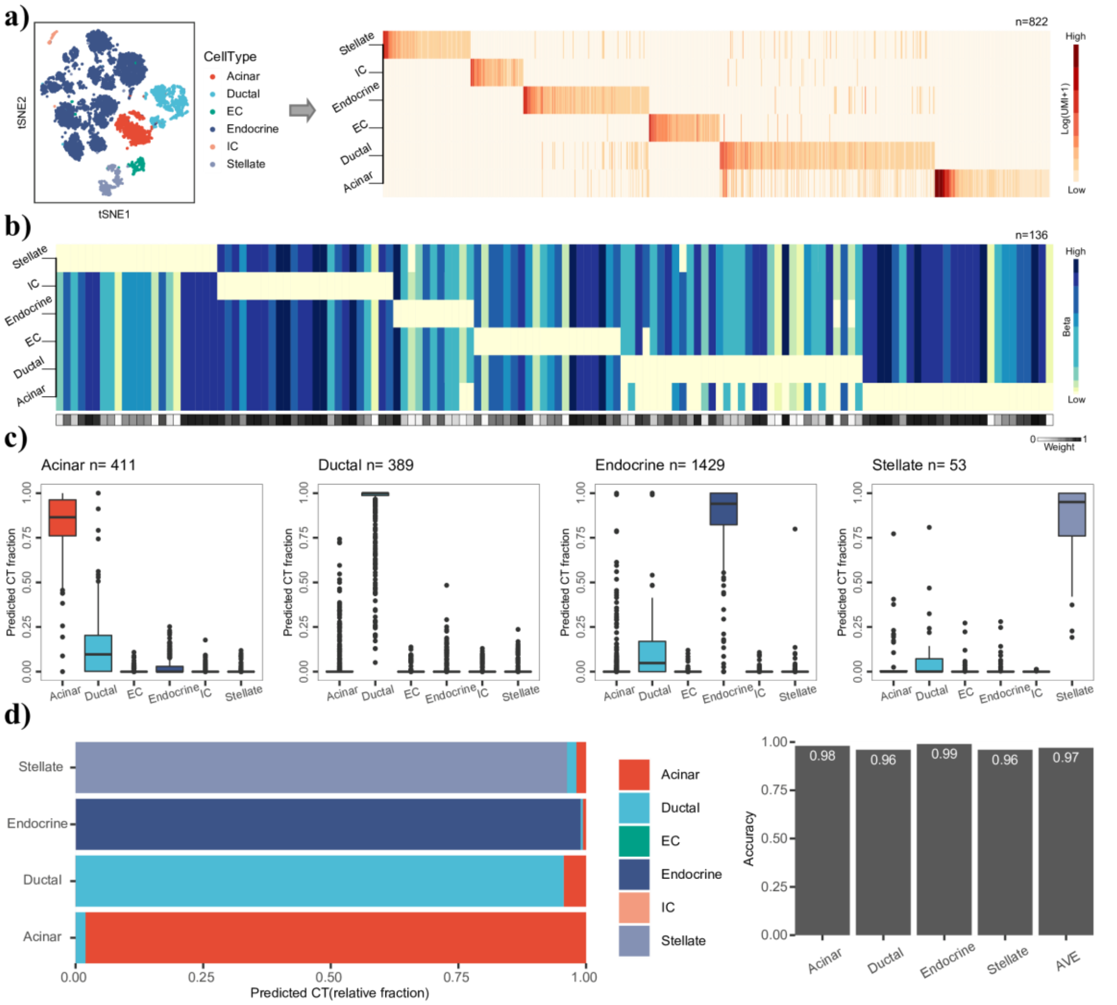

SI fig.S6: Construction and validation of the pancreas mRNA expression reference matrix. **a)** tSNE diagram and scRNA-seq expression reference matrix over 6 main pancreas cell subtypes and 822 marker genes, constructed from Baron et al's inDrop scRNA-Seq data. **b)** Heatmap displaying the imputed DNAm reference matrix for 136 marker genes. **c)** Boxplots of estimated cell-type fractions for each single cell from Enge et al's pancreas Smart-Seq2 dataset. The number of single cells of each pancreatic cell type is given. Cell-type fractions were estimated using RPC with the scRNA-Seq expression reference as given in a). **d)** Left: Barplot displaying the relative fractions (x-axis) of cells of a given cell-type (labeled on y axis) that were predicted to be acinar cells, ductal cells (Duct), endothelial cells (EC), endocrine cells (endocrine), immune cells (IC) and stellate cells (stellate), based on the cell-type fraction estimates obtained in c). Right: Barplot displaying the overall accuracy of the classification. In all boxplots, the central bar denotes the median, the box-width defines the IQR and whiskers extend to 1.5 IQR in either direction.

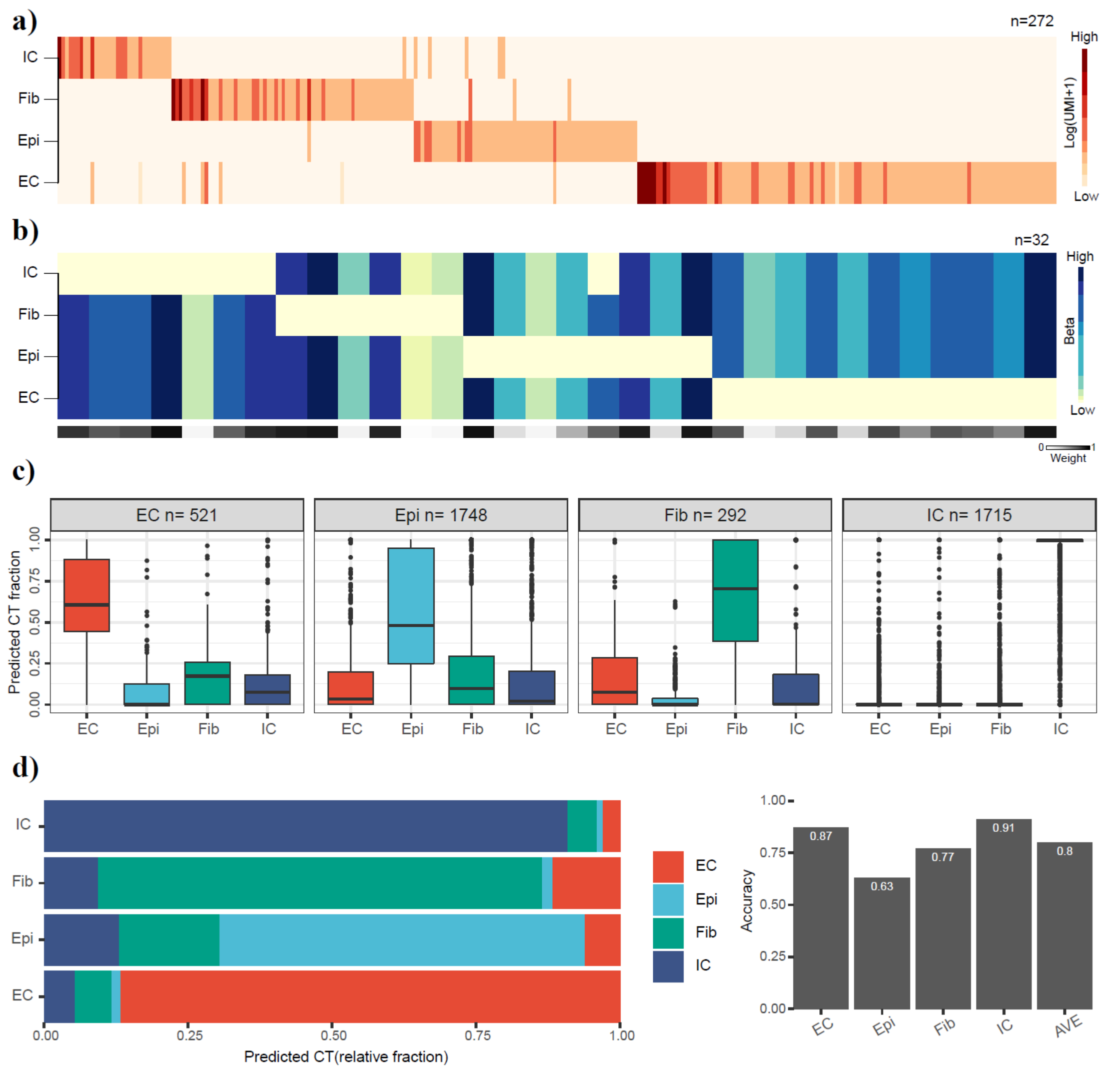

SI fig.S7: Construction and validation of the kidney mRNA expression reference matrix. **a)** The scRNA-seq expression reference matrix over 4 main kidney cell subtypes and 272 marker genes, constructed with a 10x kidney scRNA-seq dataset from Muto et al. **b)** Heatmap displaying the imputed DNAm reference matrix for 32 marker genes. **c)** Boxplots of estimated cell-type fractions for each single cell from Wu et al's 10x dataset. The number of single cells of each kidney cell type is given. Cell-type fractions were estimated using RPC with the scRNA-Seq expression reference as given in a). **d)** Left: Barplot displaying the relative fractions (x-axis) of cells of a given cell-type (labeled on y axis) that were predicted to be endothelial cells (EC), epithelial cells (Epi), fibroblasts (Fib) and immune cells (IC), based on the cell-type fraction estimates obtained in c). Right: Barplot displaying the overall accuracy of the classification. In all boxplots, the central bar denotes the median, the box-width defines the IQR and whiskers extend to 1.5 IQR in either direction.

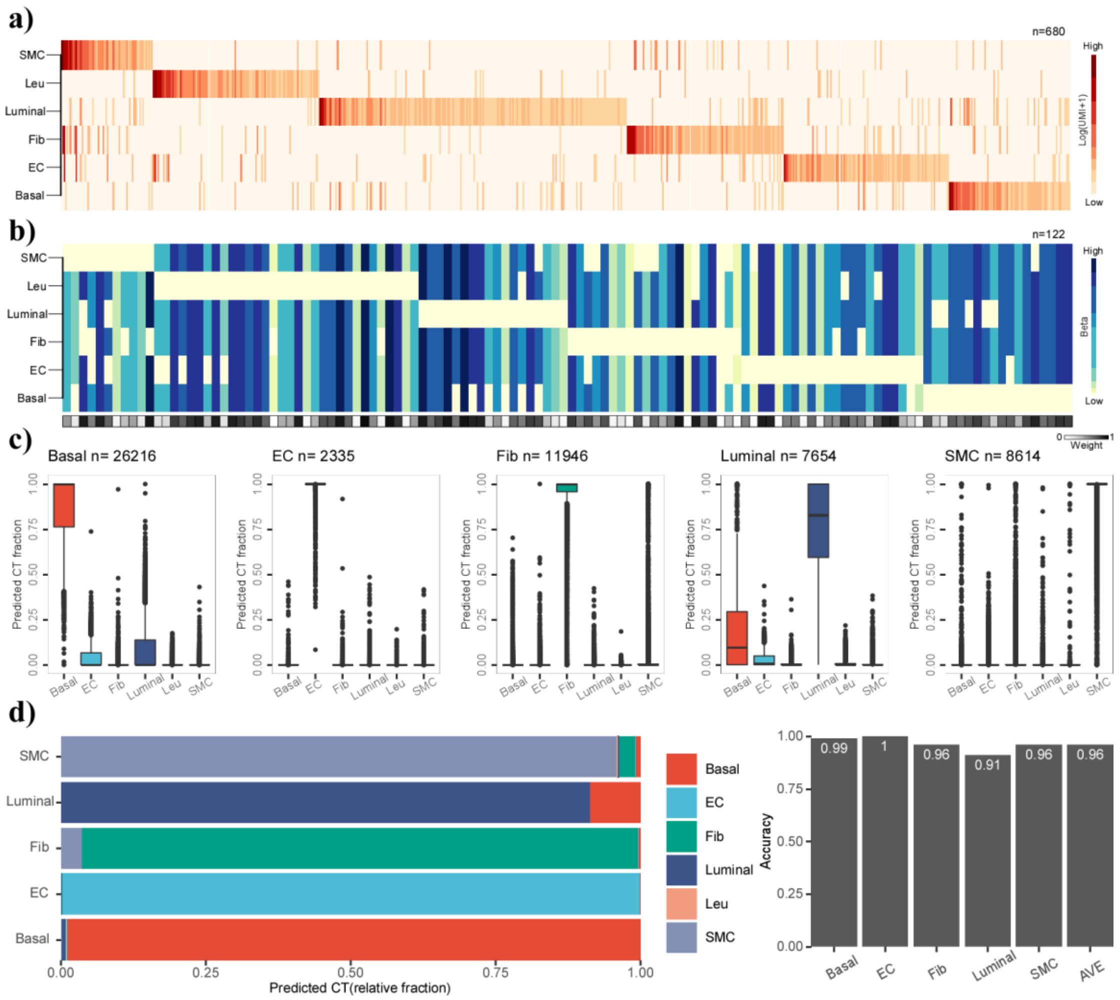

SI fig.S8: Construction and validation of the prostate mRNA expression reference matrix. a) The scRNA-seq expression reference matrix over 6 main prostate cell subtypes and 680 marker genes, constructed from Henry et al's 10x dataset (Sample 3). **b)** Heatmap displaying the imputed DNAm reference matrix for 122 marker genes. **c)** Boxplots of estimated cell-type fractions for each single cell from Henry et al's 10x dataset (Sample 1&2). The number of single cells of each prostate cell type is given. Cell-type fractions were estimated using RPC with the scRNA-Seq expression reference as given in a). **d)** Left: Barplot displaying the relative fractions (x-axis) of cells of a given cell-type (labeled on y axis) that were predicted to be basal cells (Basal), endothelial cells (EC), fibroblasts (Fib), luminal cells (Luminal), immune cells (IC) and smooth muscle cells (SMC), based on the cell-type fraction estimates obtained in c). Right: Barplot displaying the overall accuracy of the classification. In all boxplots, the central bar denotes the median, the box-width defines the IQR and whiskers extend to 1.5 IQR in either direction.

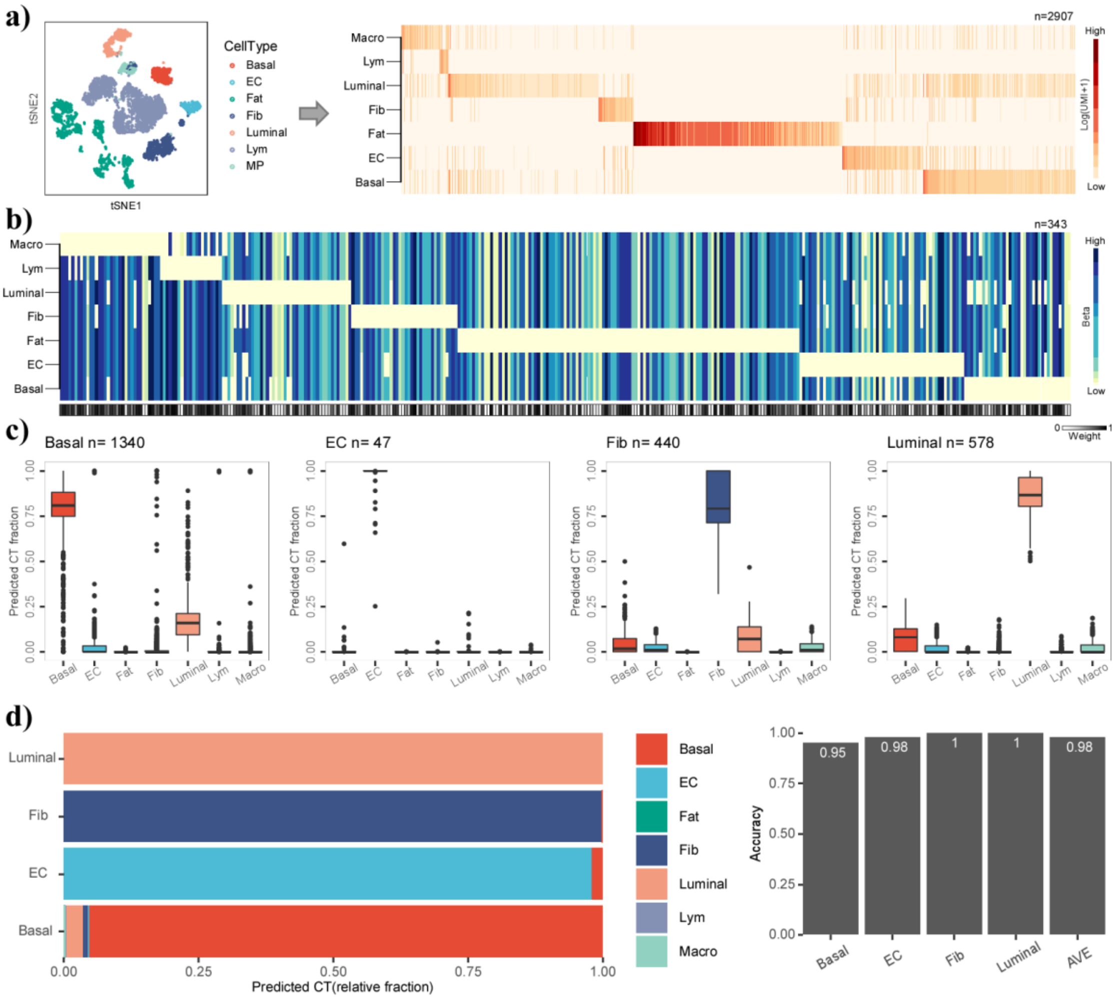

SI fig.S9: Construction and validation of the breast mRNA expression reference matrix. a) tSNE diagram and the scRNA-seq expression reference matrix over 7 main breast cell subtypes and 2907 marker genes, constructed from MCA 10x mammary gland and SmartSeq2 subcutaneous fat dataset. **b)** Heatmap displaying the imputed DNAm reference matrix for 343 marker genes. **c)** Boxplots of estimated cell-type fractions for each single cell from MCA SS2 mammary gland dataset. The number of single cells of each breast cell type is given. Cell-type fractions were estimated using RPC with the scRNA-Seq expression reference as given in a). **d)** Left: Barplot displaying the relative fractions (x-axis) of cells of a given cell-type (labeled on y axis) that were predicted to be basal cells (Basal), endothelial cells (EC), fat cells (Fat), fibroblast (Fib), luminal cells (Luminal), lymphocytes (Lym) and macrophages (Macro), based on the cell-type fraction estimates obtained in c). Right: Barplot displaying the overall accuracy of the classification. In all boxplots, the central bar denotes the median, the box-width defines the IQR and whiskers extend to 1.5 IQR in either direction.

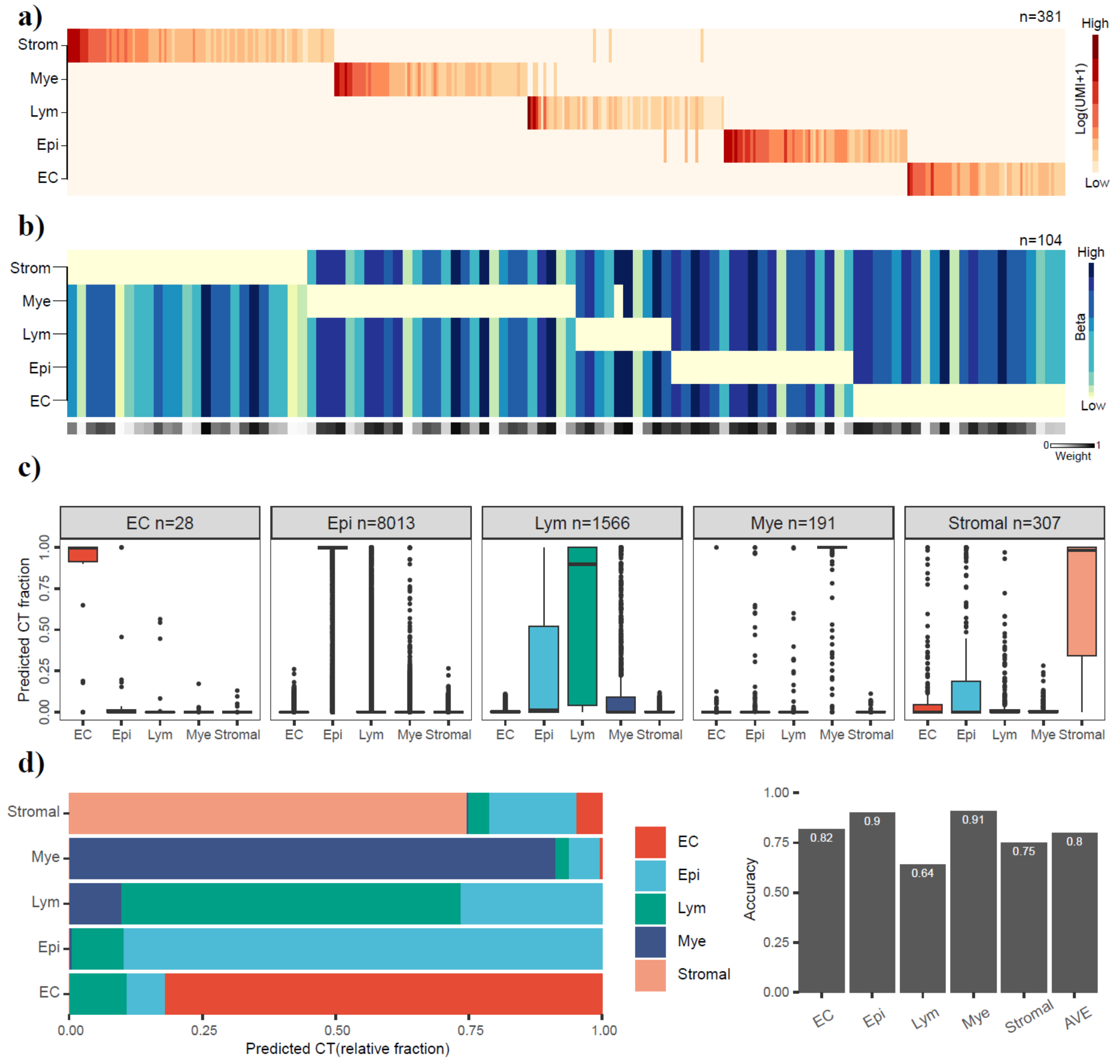

SI fig.S10: Construction and validation of the colon mRNA expression reference matrix. a) The scRNA-seq expression reference matrix over 5 main colon cell subtypes and 381 marker genes, constructed with a 10X human colon dataset from Elmentaite et al. **b)** Heatmap displaying the imputed DNAm reference matrix for 104 marker genes. **c)** Boxplots of estimated cell-type fractions for each single cell from HCL colon and rectum data. The number of single cells of each colon cell type is given. Cell-type fractions were estimated using RPC with the scRNA-Seq expression reference as given in a). **d)** Left: Barplot displaying the relative fractions (x-axis) of cells of a given cell-type (labeled on y axis) that were predicted to be endothelial cells (EC), epithelial cells (Epi), lymphocytes (Lym), myeloids (Mye), stromal cells (Stromal), based on the cell-type fraction estimates obtained in c). Right: Barplot displaying the overall accuracy of the classification. In all boxplots, the central bar denotes the median, the box-width defines the IQR and whiskers extend to 1.5 IQR in either direction.

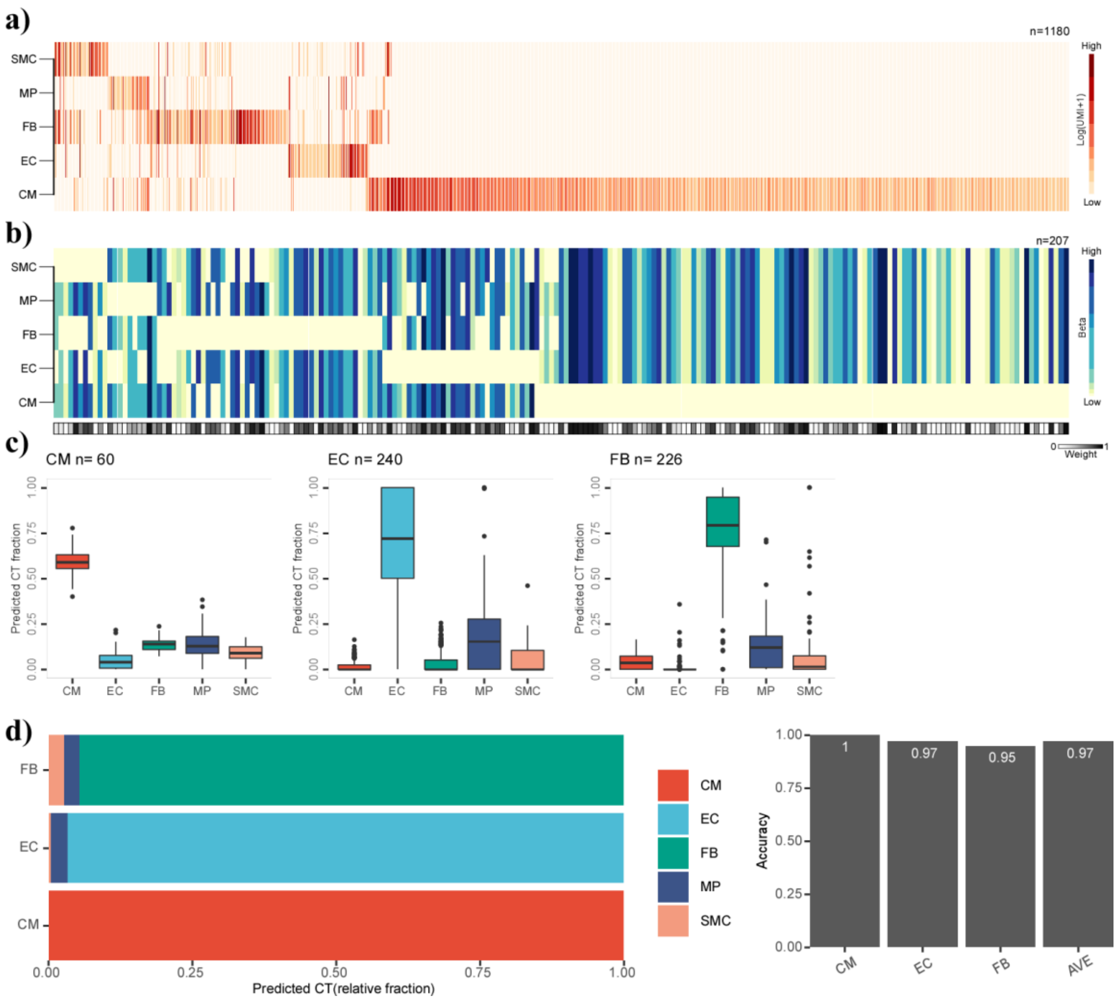

SI fig.S11: Construction and validation of the heart mRNA expression reference matrix. **a)** The scRNA-seq expression reference matrix over 5 main heart cell subtypes and 1180 marker genes, constructed from Wang et al's 10x data. **b)** Heatmap displaying the imputed DNAm reference matrix for 207 marker genes. **c)** Boxplots of estimated cell-type fractions for each single cell from MCA 10x heart and aorta data. The number of single cells of each heart cell type is given. Cell-type fractions were estimated using RPC with the scRNA-Seq expression reference as given in a). **d)** Left: Barplot displaying the relative fractions (x-axis) of cells of a given cell-type (labeled on y axis) that were predicted to be cardiac muscle cells (CM), endothelial cells (EC), fibroblast (Fib), macrophages (Macro), smooth muscle cells (SMC), based on the cell-type fraction estimates obtained in c). Right: Barplot displaying the overall accuracy of the classification. In all boxplots, the central bar denotes the median, the box-width defines the IQR and whiskers extend to 1.5 IQR in either direction.

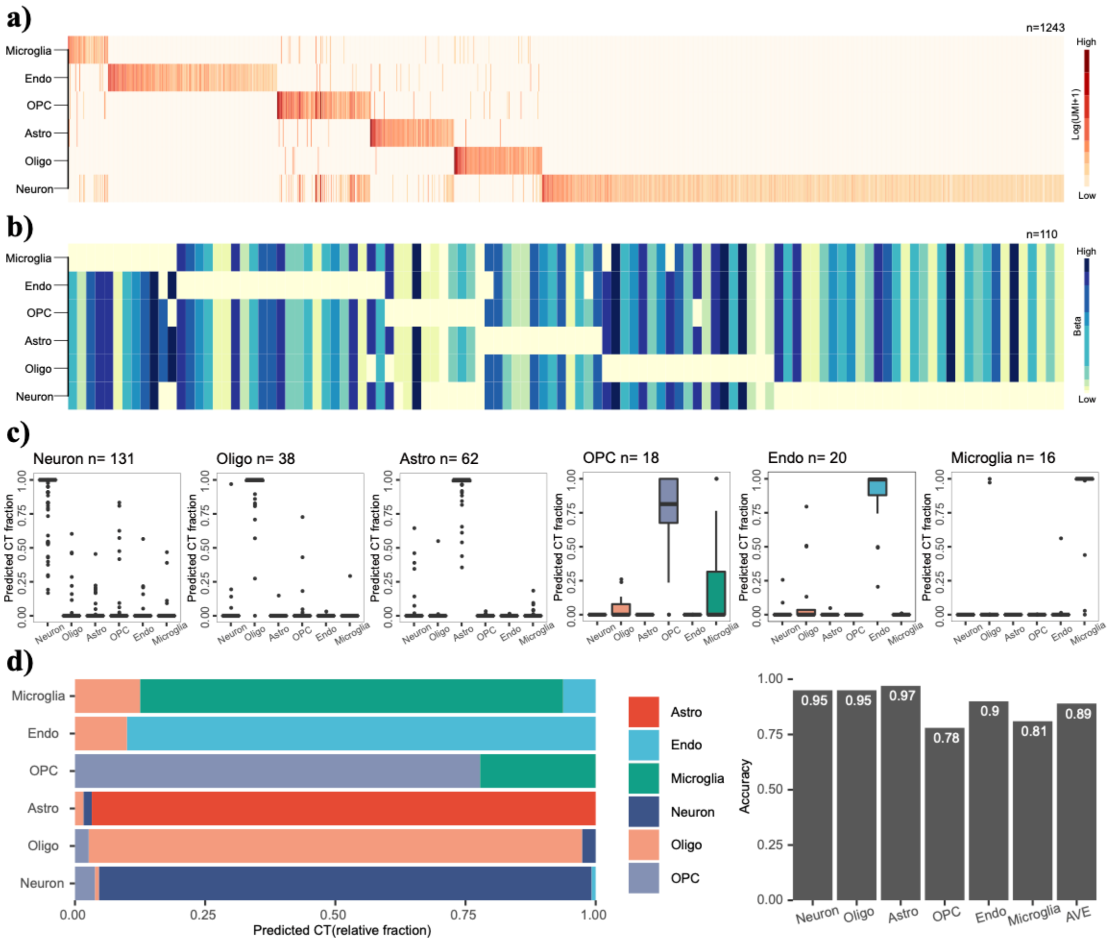

SI fig.S12: Construction and validation of brain mRNA expression reference matrix. a) The scRNA-seq expression reference matrix over 6 main brain cell subtypes and 1243 marker genes, constructed from Li et al's 10x data. **b)** Heatmap displaying the imputed DNAm reference matrix for 110 marker genes. **c)** Boxplots of estimated cell-type fractions for each single cell from Darmanis et al's adult brain Smart-Seq data. The number of single cells of each brain cell type is given. Cell-type fractions were estimated using RPC with the scRNA-Seq expression reference as given in a). **d)** Left: Barplot displaying the relative fractions (x-axis) of cells of a given cell-type (labeled on y axis) that were predicted to be neurons, astrocytes (Astro), oligodendrocytes (Oligo), endothelial cells (Endo), microglia, and oligodendrocyte progenitor cells (OPC), smooth muscle cells (SMC), based on the cell-type fraction estimates obtained in c). Right: Barplot displaying the overall accuracy of the classification. In all boxplots, the central bar denotes the median, the box-width defines the IQR and whiskers extend to 1.5 IQR in either direction.

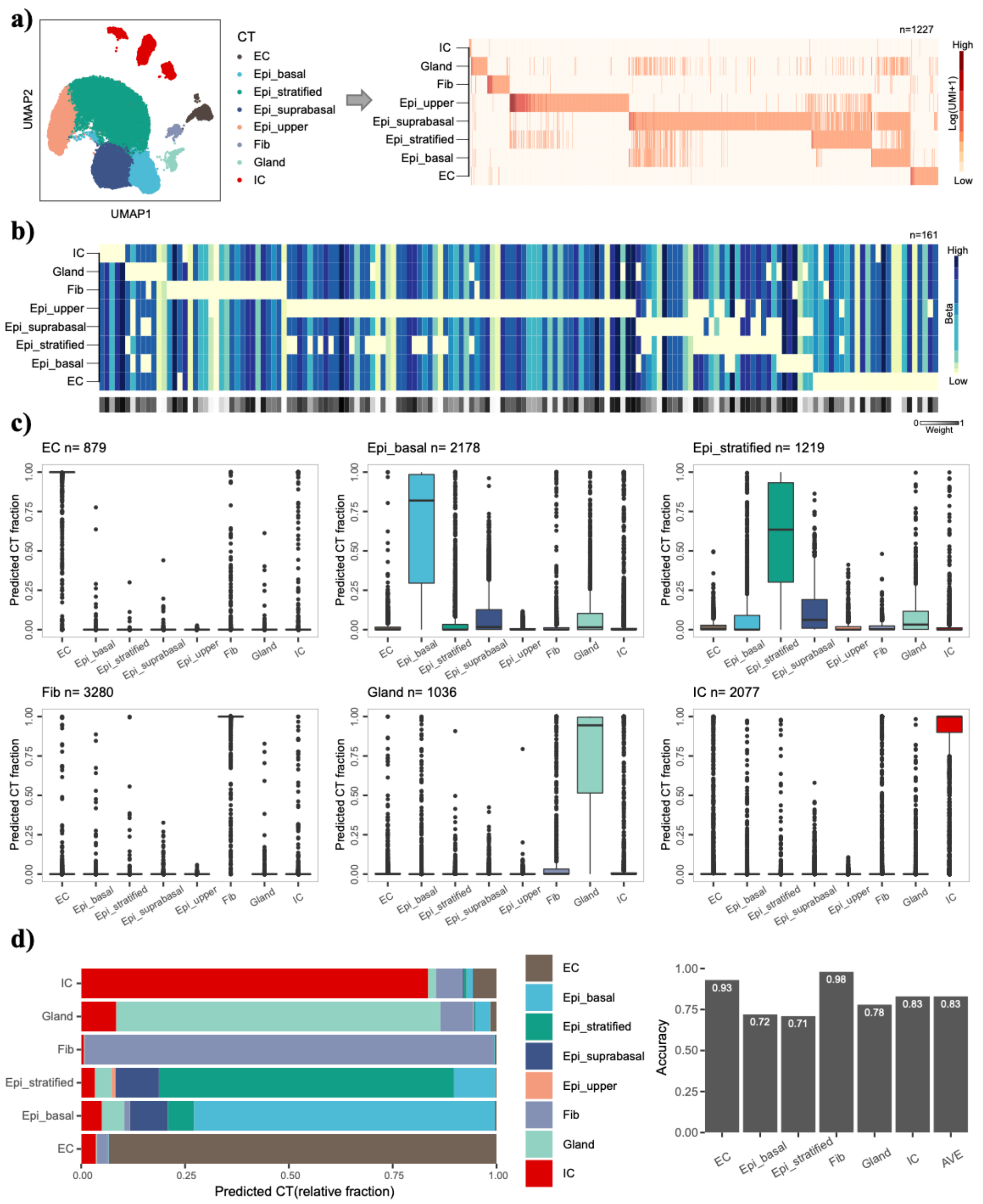

SI fig.S13: Construction and validation of esophagus mRNA expression reference matrix. a) The scRNA-seq expression reference matrix over 8 main esophagus cell subtypes and 1227 marker genes, constructed from Madissoon et al's 10x data. **b)** Heatmap displaying the imputed DNAm reference matrix for 161 marker genes. **c)** Boxplots of estimated cell-type fractions for each single cell from HCL adult esophagus Microwell-Seq data. The number of single cells of each esophagus cell type is given. Cell-type fractions were estimated using RPC with the scRNA-Seq expression reference as given in a). **d)** Left: Barplot displaying the relative fractions (x-axis) of cells of a given cell-type (labeled on y axis) that were predicted to be endothelial cells (EC), basal cells (Epi_basal), stratified cells (Epi_stratified), suprabasal (Epi_suprabasal), upper epithelial cells (Epi_upper), fibroblasts (Fib), gland cells (Gland) and immune cells (IC), based on the cell-type fraction estimates obtained in c). Right: Barplot

displaying the overall accuracy of the classification. In all boxplots, the central bar denotes the median, the box-width defines the IQR and whiskers extend to 1.5 IQR in either direction.

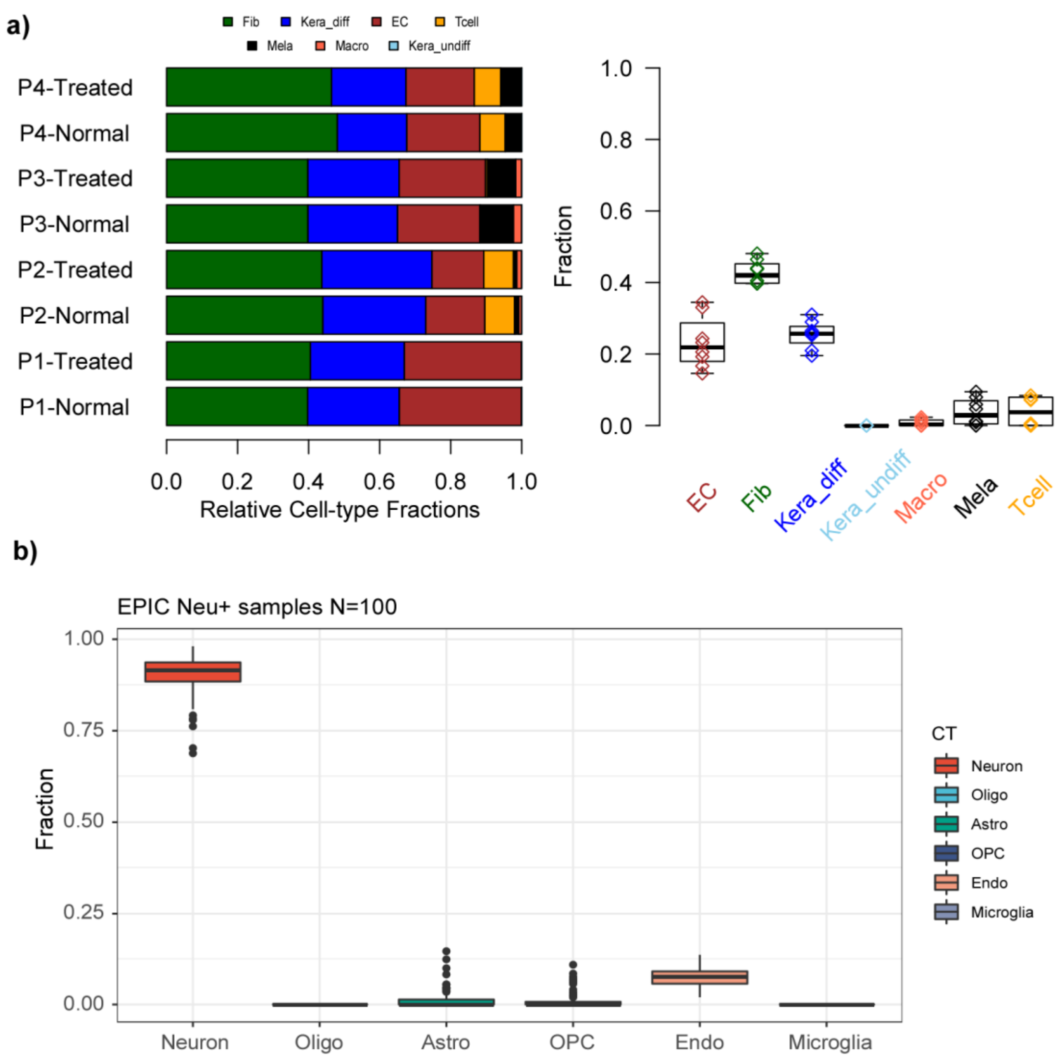

SI fig.S14: Validation of skin and brain DNAm reference matrices in EPIC DNAm datasets. a) Left barplots display the estimated cell-type fractions using our skin DNAm reference matrix in each of 8 skin fibroblast samples (EPIC data from Sarkar et al). Boxplots to the right help the visual comparison between cell-types, confirming that all 8 samples would be classified as fibroblasts. **b)** Boxplots display the estimated cell-type fractions using our brain DNAm reference matrix in 100 neuronal (Neu+) samples (EPIC data from Pai et al). In all boxplots, the central bar denotes the median, the box-width defines the IQR and whiskers extend to 1.5 IQR in either direction.

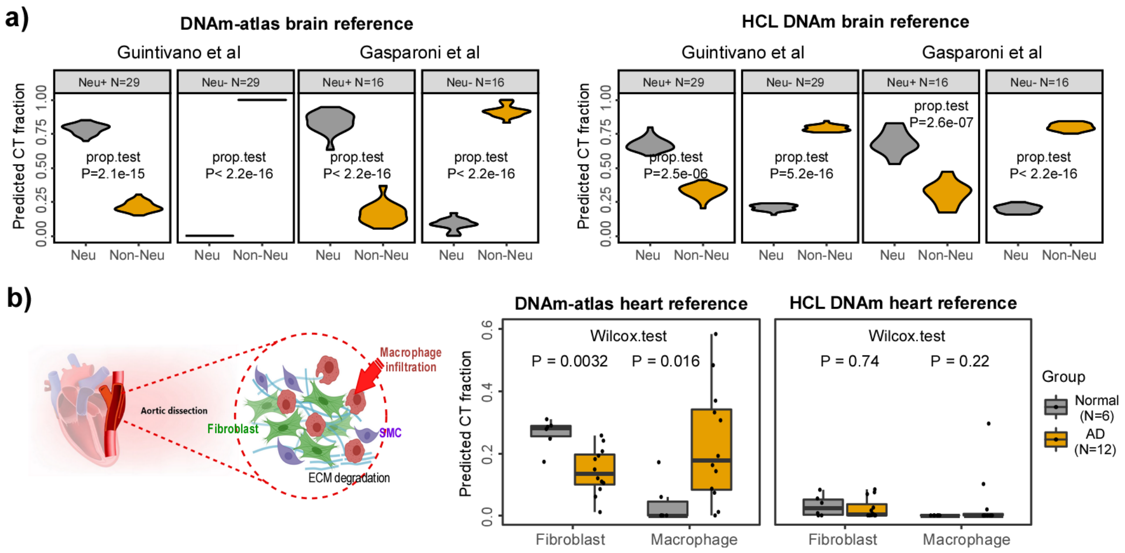

SI fig.S15: Comparison between DNAm-atlas and HCL-derived DNAm reference matrices. a) Violin plots depicting predicted cell-type (CT) fractions (y-axis) of neuronal (Neu+) and non-neuronal (Neu-) samples from Guintivano and Gasparoni et al DNAm datasets (in Gasparoni only control samples were included), using the brain DNAm reference matrix from our DNAm-atlas (left) and an analogous one built by starting out from the HCL scRNA-Seq brain dataset (right). Predicted CT fractions are shown for neurons (Neu) and for all other brain cell subtypes combined (Non-Neu) (x-axis). In each case, we assess separability of the Neu and Non-Neu fractions using a two-tailed test of proportions with the associated P-value shown in plots. **b)** Aortic dissection is characterized by an increased macrophage infiltration and a concomitant degradation of the extracellular matrix (ECM) (i.e. a reduction in fibroblasts). Boxplots display the predicted CT fractions (y-axis) in a 450k DNAm dataset profiling 6 normal and 12 aortic dissection (AD) samples, obtained using either the heart DNAm reference matrix from our DNAm-atlas (left) or an analogous one built from the HCL heart scRNA-Seq dataset (right). Here we compare fractions between normal and AD samples using a one-tailed Wilcoxon rank sum test. In all boxplots, the central bar denotes the median, the box-width defines the IQR and whiskers extend to 1.5 IQR in either direction.

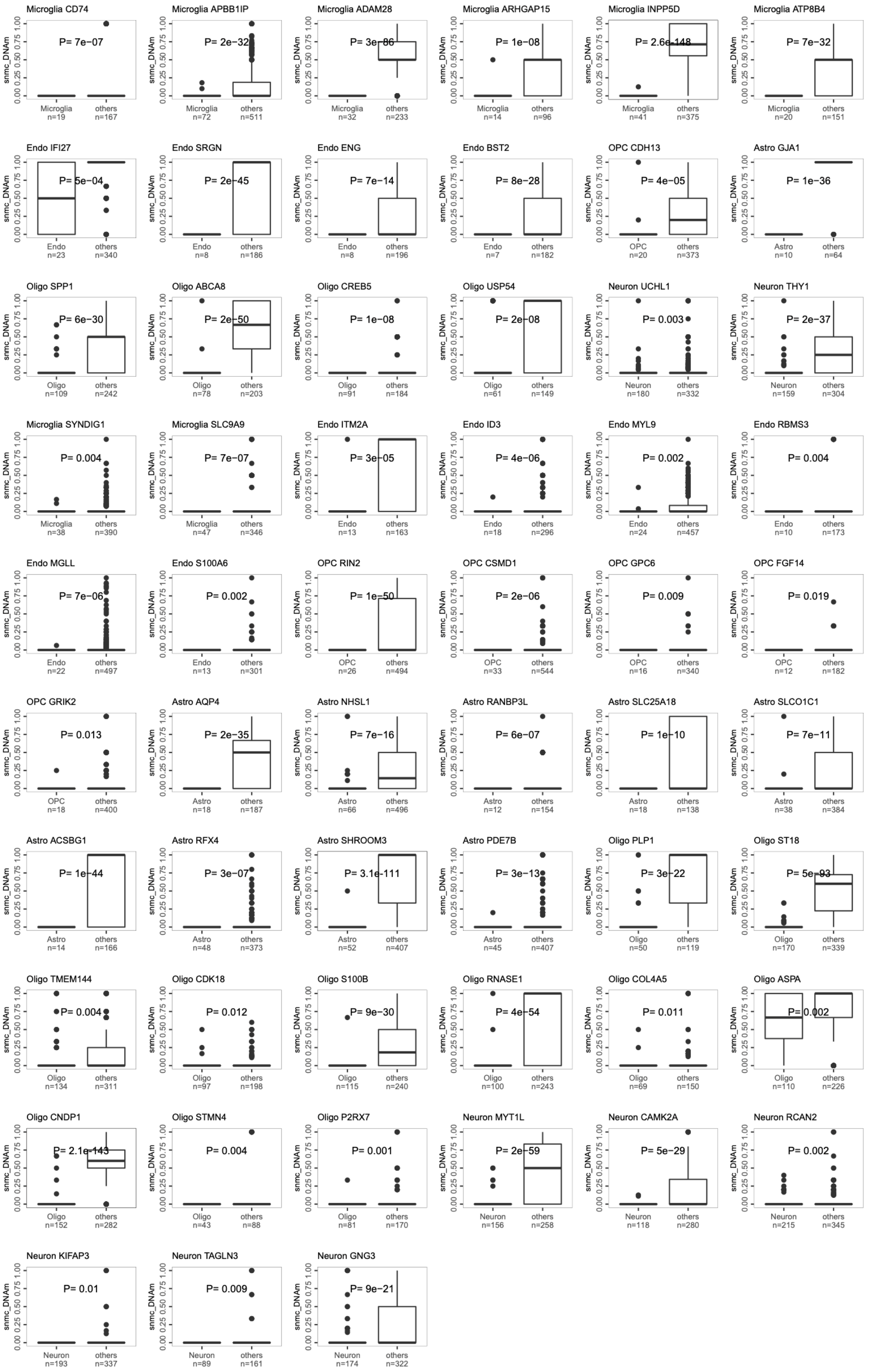

SI fig.S16: Validation of differential methylation markers in snmC-Seq data from brain.

Boxplots of Bernoulli DNAm level of 57 markers showing significant hypomethylation (FDR<0.05) pattern in the cells where the marker gene is expressed compared to the other cell-types where it is not. P-value from a two-tailed Wilcoxon rank sum test is given. In all boxplots, the central bar denotes the median, the box-width defines the IQR and whiskers extend to 1.5 IQR in either direction.

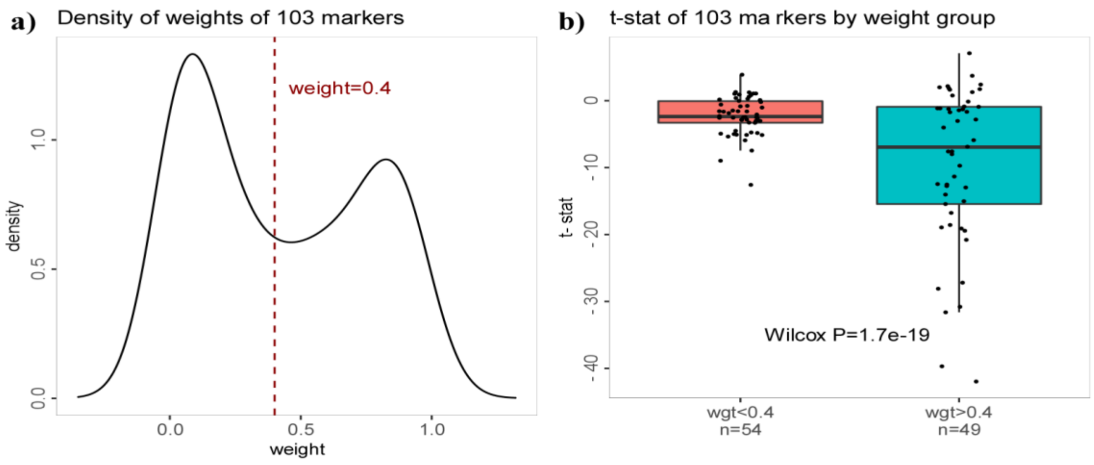

SI fig.S17: EpiSCORE confidence weights improves validation accuracy. a) Density distribution of the confidence weights of all 103 markers in the brain DNAm reference matrix. **b)** Boxplots of t-statistics comparing the promoter DNAm level in cells expressing the markers against the cells not expressing the markers, as computed in the snmC-seq2 dataset. The 103 marker genes have been grouped according to a weight<0.4 and weight>0.4. The threshold 0.4 is derived from the density distribution of weights, which is bi-modal with 0.4 the boundary between modes, as shown in panel-a. P-value derives from a one-tailed Wilcoxon rank sum test comparing the weight<0.4 group against weight>0.4 group. The number of marker-genes in each group is given. In all boxplots, the central bar denotes the median, the box-width defines the IQR and whiskers extend to 1.5 IQR in either direction.

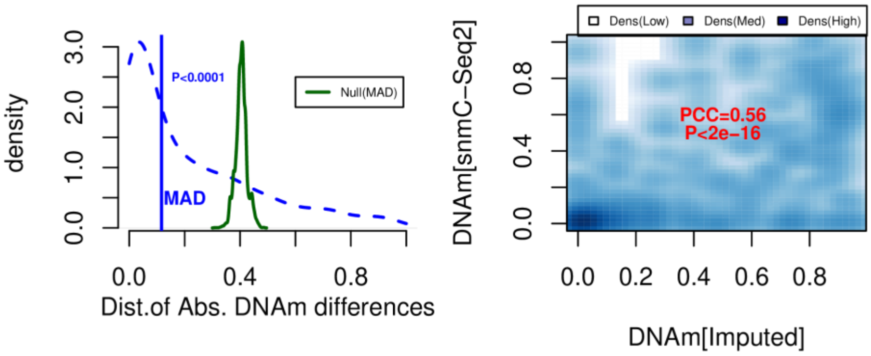

SI fig.S18: Direct comparison of brain DNAm reference matrix to pseudo-bulk reference derived from snmC-Seq2 data. Left panel displays the distribution of absolute differences between the imputed and the pseudo-bulk snmC-Seq2 DNAm data matrices (dashed-curve), with the median absolute deviation (MAD) indicated by a vertical solid blue line. Green curve denotes the MAD values from 10,000 Monte-Carlo randomizations (null distribution). P-value is obtained by comparing the observed MAD value to the null distribution. Right panel is a smoothed scatterplot of the imputed DNAm values and the pseudo-bulk snmC-Seq2 ones. PCC denotes the Pearson Correlation Coefficient and Correlation test P-value is given.

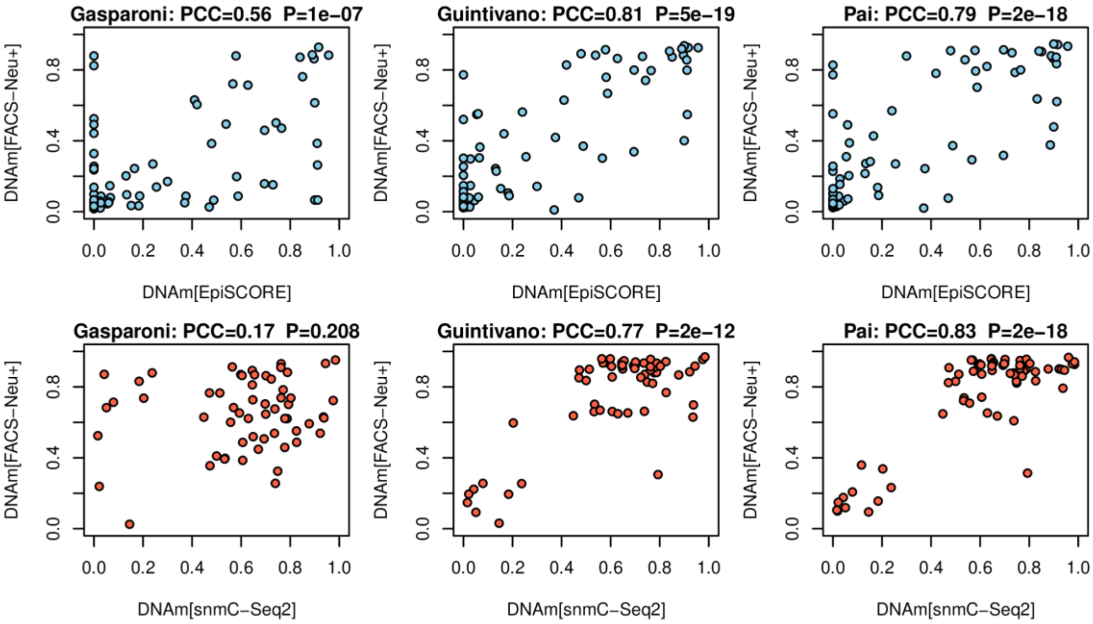

SI fig.S19: Direct comparison of the neuron DNAm reference profiles with DNAm profile of FACS-sorted neuron samples. Top row compares the DNAm profile for neurons as given by the EpiSCORE derived DNAm reference matrix (x-axis) against the DNAm profile obtained by averaging FACS-sorted Neu+ samples (y-axis) from 3 different Illumina DNAm studies (Gasparoni et al, Guintivano et al and Pai et al). Pearson Correlation Coefficient (PCC) and P-value are given. Bottom row is similar to top-row but now using the neuron DNAm profile from the snmC-Seq2 derived DNAm reference matrix.

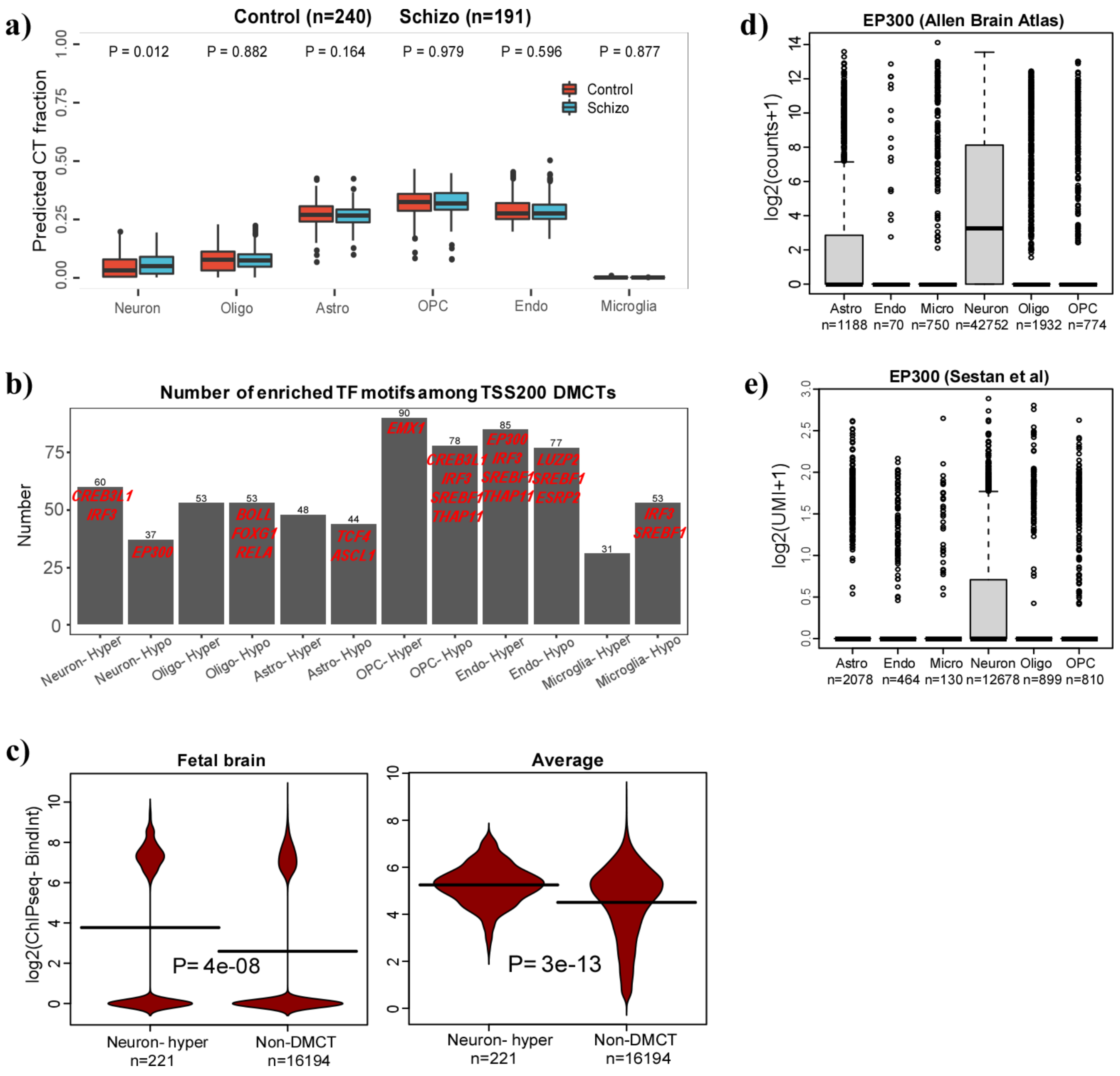

SI fig.S20: a) Predicted cell-type fractions (y-axis) from our brain DNAm reference matrix for the 6 brain cell-types, as applied to prefrontal cortex samples from an EWAS in schizophrenia (SZ), comparing cases and controls. P-values derive from a two-tailed Wilcoxon rank sum test. **b)** Number of enriched TF-binding motifs among the TSS200 regions of DMCT genes, stratified by category of DMCT (x-axis). In red we highlight some of the top-ranked TFs. **c)** Violin plots comparing the ChIP-Seq binding intensity of EP300, between genes linked to neuron SZ-hypermethylated DMCTs ("Neuron-hyper") vs. genes not linked to any DMCT ("non-DMCT") for the case where the binding intensity was obtained from fetal brain samples (left panel) and when averaged over all available samples (right panel). P-value is from a one-tailed Wilcoxon rank sum-test. **d-e)** Expression of EP300 vs brain cell type for the Allen Brain Atlas (top) and the scRNA-Seq atlas from Seston et al (bottom). In all boxplots, the central bar denotes the median, the box-width defines the IQR and whiskers extend to 1.5 IQR in either direction.

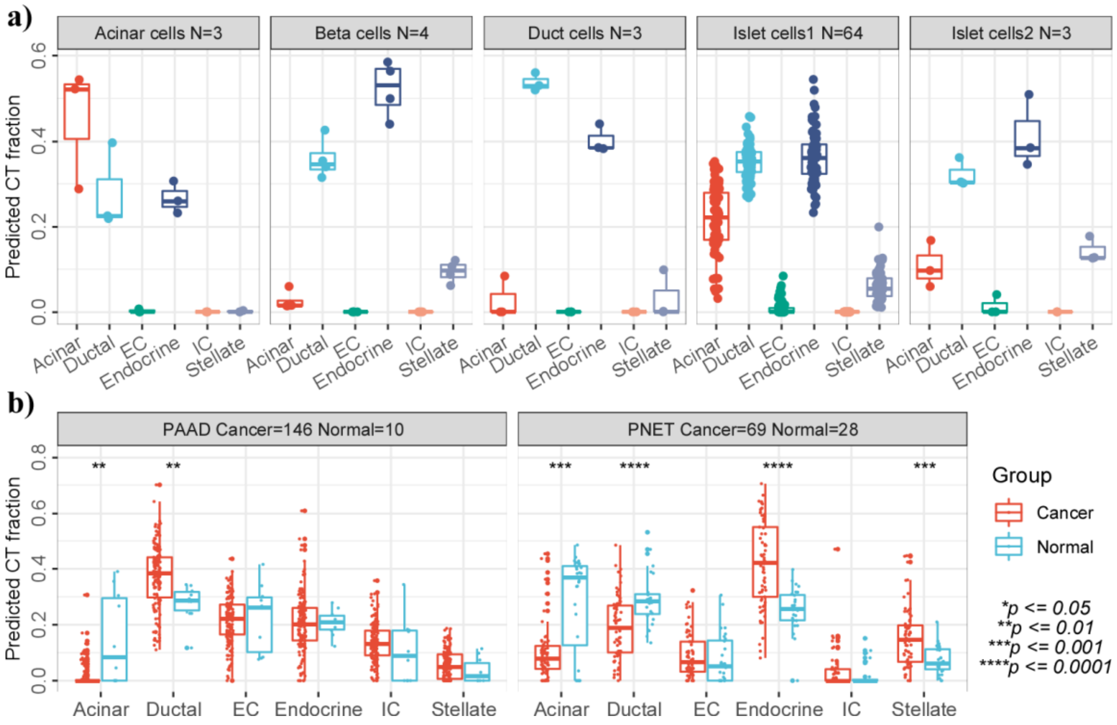

SI fig.S21: Validation of 6 cell-type pancreatic DNAm reference. a) Estimated cell-type fractions in DNAm datasets of acinar, beta and ductal samples (Moss et al) and islet samples: Islet1 (Syed et al), Islet2 (Stefan-Lifshitz et al). **b)** Estimated cell-type fractions in the pancreatic ductal adenocarcinoma (PAAD/PDAC) dataset from the TCGA and in a series of pancreatic neuroendocrine tumors (PNET) from Pipinikas et al. P-values derive from a two-tailed Wilcoxon rank sum test. In all boxplots, the central bar denotes the median, the box-width defines the IQR and whiskers extend to 1.5 IQR in either direction.

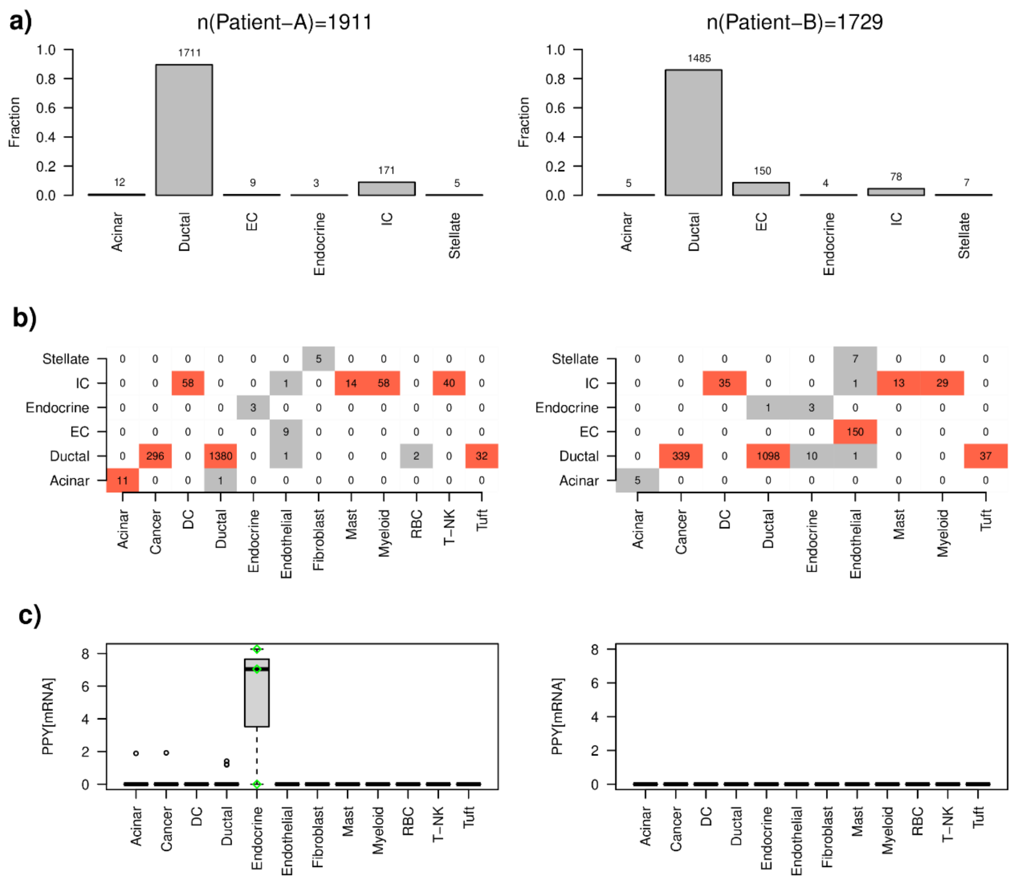

SI fig.S22: Validation of 6 cell-type pancreas mRNA reference and PPY expression. a) Estimated cell-type fractions in PDAC patients A and B from Moncada R et al Nat Biotechnology 2020, as obtained by applying our pancreas mRNA expression reference matrix to their scRNA-Seq data, annotating cells to cell-types using a maximum-weight criterion. The numbers of cells are given above bars. **b)** Confusion matrix between our predicted cell-type annotation (y-axis) and the annotation provided by Moncada (x-axis). IC=Immune Cell, EC=Endothelial cell. ICs include myeloid, Dendritic cells (DCs), T-NK cells and Mast cells. **c)** Boxplots of PPY expression (a gamma-cell marker) against cell-type in the same Moncada scRNA-Seq data.

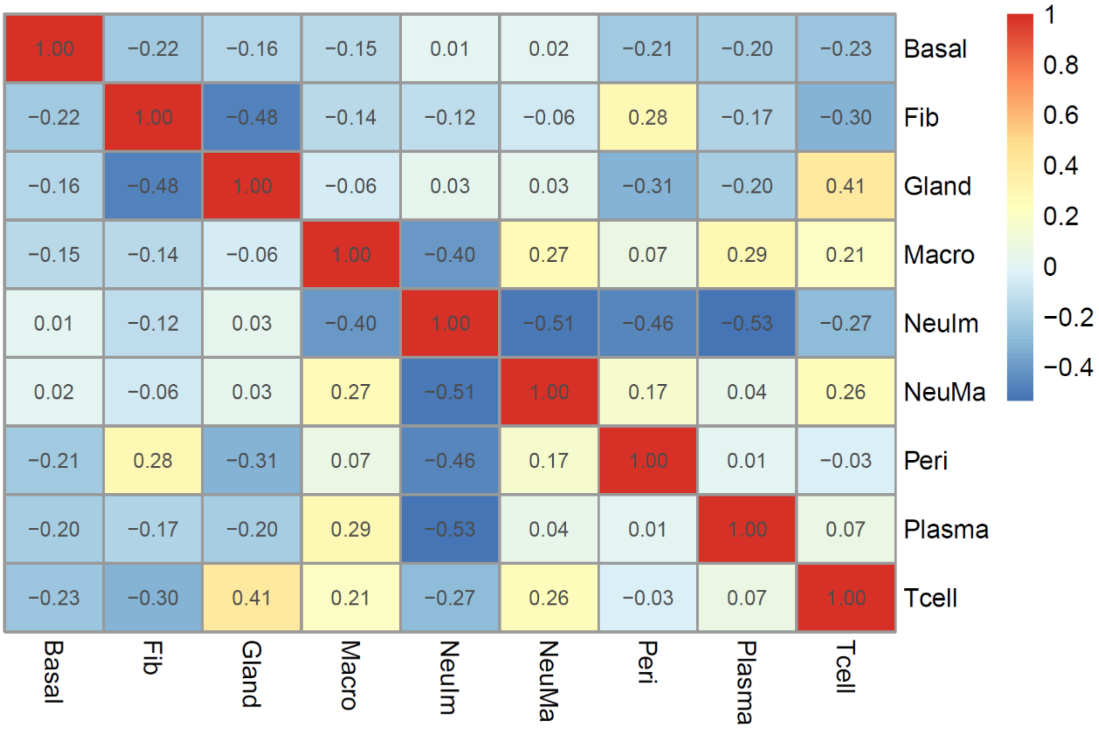

SI fig.S23: Pearson correlations between estimated cell type fractions in olfactory neuroblastoma (ONB). Heatmap displays the Pearson Correlation Coefficients between the estimated cell-type fractions, as computed over the 66 ONB samples from Capper et al.

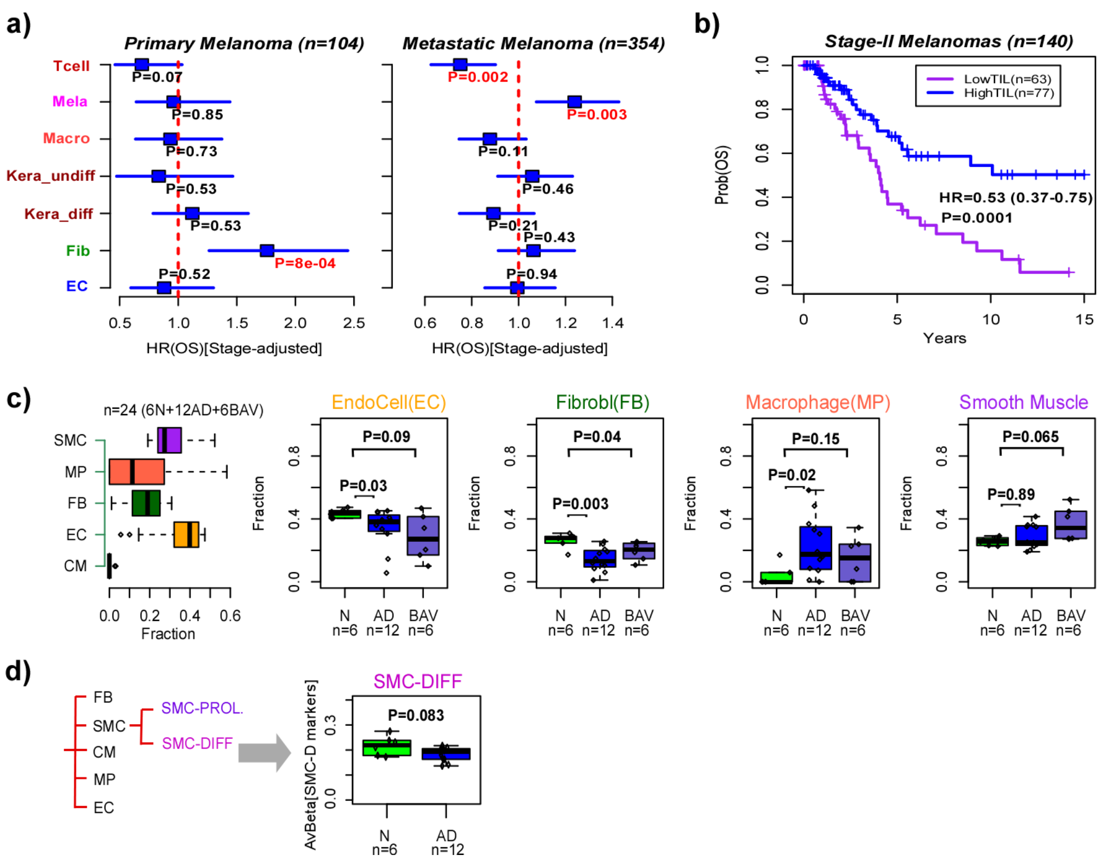

SI fig.S24: EpiSCORE-atlas reveals novel insights in melanoma and aortic dissection. a) Hazard Ratio (HR) Forest plots derived from Cox-regressions of overall survival vs. estimated cell-type fractions in primary and metastatic melanomas from the TCGA. HRs, 95% confidence intervals and P-values are derived from a multivariate Cox-regression which includes stage (the only other prognostic predictor) as a covariate. **b)** Kaplan Meier-curves for overall survival for patients with stage-2 melanoma, stratified according to the level of tumor infiltrating lymphocytes (TIL). Samples with less than 1% TIL fraction were placed in the low-TIL category, the rest in high-TIL. All events are censored at 15 years. The HR, 95% confidence interval and Chi-Square test P-value in KM-plot derive from a Cox-regression of overall survival against TIL fraction using TIL as a continuous value, not the binarized version. **c)** Left boxplot displays the estimated cell-type fractions for smooth muscle cells (SMC), macrophages (MP), fibroblasts (FB), endothelial cells (EC) and cardiomyocytes (CM) for all 24 aorta samples, of which 6 are normal (N), 12 from aortic dissection (AD) cases, and 6 from bicuspic aortic valve (BAV) cases. Boxplots to the right display the estimated EC, FB, MP and SMC fractions vs. disease status. P-values all derive from a Wilcoxon rank sum test. **d)** SMCs can be divided into contractile differentiated subtype (DIFF) and a dedifferentiated proliferative subtype (PROL). Boxplot displays the estimated SMD-DIFF fraction in normal and AD samples. P-value is from a Wilcoxon-rank sum test. In all boxplots, the central bar denotes the median, the box-width defines the IQR and whiskers extend to 1.5 IQR in either direction.

Supplementary methods

Construction and validation of scRNA-seq reference matrices

Lung: The lung expression reference matrix was constructed with scRNA-seq data from Travaglini et al [1]. This is a human lung 10X scRNA-seq dataset from 3 patients. The processed Seurat R object can be downloaded from https://www.synapse.org/#!Synapse:syn21041850/wiki/600865. The data is in log scale. We used patient 3 sample for reference construction and the other samples for validation, because P3 sample has a larger number of cells as well as a more balanced number of different cell types. We grouped the cells from P3 sample into 3396 endothelial cells (EC), 6294 epithelial cells (Epi), 1019 granulocytes (Gran), 4697 lymphocytes (Lym), 5393 macrophages (Macro), 1285 monocytes (Mono) and 2571 stromal cells (Stromal). The expression reference matrix was constructed with EpiSCORE using marker specificity scores MSS= (4,6,3,3,6,3,4), which resulted in 1788 markers. The expression reference matrix was then validated in Travaglini P1 and P2 samples.

Bladder: The bladder expression reference matrix was constructed with a human bladder 10X dataset from Yu et al [2]. The data was downloaded from https://www.ncbi.nlm.nih.gov/geo/query/acc.cgi?acc=GSE129845 and we processed and annotated it with Seurat3.0 pipeline[3] using the same parameter choice from paper. Cells with >200 and <4000 genes expressed and with mitochondrial contents<10% were retained. *NormalizeData* function was used to log-normalize the data with scaling factor 10000. We identified highly variable genes as mean expression between 0.0125 and 3, and dispersion>0.5. We then used PCA to select 15 significant principal components, with variable genes as input. Cells were clustered by *FindNeighbors* and *FindClusters* function and classified into 18 clusters, with resolution=0.6. We assigned a cell type to each cluster based on canonical marker gene expression from the same paper[2]. After annotation, we have 361 endothelial cells (EC), 4950 epithelial cells (Epi), 3350 fibroblasts (Fib) and 276 immune cells (IC). The expression reference matrix was constructed running EpiSCORE with MSS= (3,3,3,3), which resulted in 522 markers. The expression reference matrix was validated in HCL Microwell-Seq bladder dataset [4] (https://figshare.com/articles/dataset/HCL_DGE_Data/7235471). The cell types were annotated by author. The counts were divided by the total counts within the cell and then multiplied by the maximum total count across all cells. Then they were log2-transformed with pseudocount +1.

Liver: The liver expression reference matrix was constructed from a human liver 10X single cell RNA-seq data from MacParland et al [5]. The Seurat R object can be downloaded from https://github.com/BaderLab/HumanLiver. The data is in log scale. We grouped the cells into 119 cholangiocytes (Chol), 844 endothelial cells (EC), 3501 hepatocytes (Hep), 2658 immune cells (IC) and 1192 kupffer cells (Kup). The expression reference matrix was constructed with MSS= (4,4,4,4,3), which resulted in 658 markers. The expression reference matrix was validated in HCA liver mCEL-Seq2 data [6], which can be downloaded from

https://www.ncbi.nlm.nih.gov/geo/query/acc.cgi?acc=GSE124395 and annotated by the author. The counts were divided by the total counts in each cell and multiplied by the maximum total counts across cells. Then they were log2-transformed with pseudocount +1.

OE: The olfactory epithelium (OE) is constructed from a 10X single cell RNA-seq data from Durante et al [7]. The data can be downloaded from https://www.ncbi.nlm.nih.gov/geo/query/acc.cgi?acc=GSE139522 and we processed it with Seurat3.0 pipeline[3] using the same parameter choice from paper. We retained the cells with total UMI>400, with >100 and <8000 genes expressed and with mitochondrial contents<10%. *NormalizeData* function was used to log-normalize the data with scaling factor 10000. Variable genes were identified with *FindVariableFeatures* function with 5000 genes; the selection method was set to 'vst'. The data contains 2 OE samples, which were used to construct the OE reference, and 2 respiratory epithelium (RE) samples, which were used for validation. Using Seurat's *IntegrateData* function, we combined the 2 OE samples as 1 object and 2 RE samples as another one. We used the first 30 principal components from PCA to do UMAP dimensionality reduction. *FindNeighbors* and *FindClusters* functions were used to identify 32 clusters with resolution=0.8. We classified the cells into 9 groups, which are 2251 basal cells (Basal), 3841 fibroblasts (Fib), 933 gland cells (Gland), 944 macrophages (Macro), 394 immature neurons (NeuIm), 348 mature neurons (NeuMa), 2555 pericytes (Peri), 1041 plasma cells (Plasma) and 2508 T cells (Tcell), based on canonical marker gene expression from the same paper[7]. A relaxed specificity score MSS= (6,4,6,6,6,6,8,4,4) was used to build expression reference matrix, which resulted in 1889 markers.

Skin: The skin expression reference matrix was constructed from a 10X single cell RNA-seq data from Solé-Boldo et al[8] (https://www.ncbi.nlm.nih.gov/geo/query/acc.cgi?acc=GSE130973), which profiled 15147 cells, grouped into 2981 endothelial cells (EC), 5948 fibroblasts (Fib), 1399 differentiated keratinocytes (Kera_diff), 1187 undifferentiated keratinocytes (Kera_undiff), 2228 macrophages (Macro), 123 melanocytes (Mela) and 1281 T cells (Tcell). The Seurat R object and cell type annotation are provided by the author. A relaxed specificity score MSS= (3,3,3,3,3,6,3) was used to build expression reference matrix, which resulted in 652 markers. We validated the reference matrix in the MCA1 Smart-Seq2 single cell skin dataset [9] and another human melanocytes Smart-Seq2 dataset from Le et al (https://www.ncbi.nlm.nih.gov/geo/query/acc.cgi?acc=GSE151091). For MCA1 data, the processing steps are the same as described previously. For melanocytes data, we retained the cells with >500 and <10000 genes expressed and with mitochondrial contents<5%. Then we normalized the counts by library size and log2-transformed with pseudocount +1.

Pancreas: The pancreas expression reference matrix was constructed with scRNA-seq data from Baron et al[10]. This is a human pancreas inDrop scRNA-seq dataset. The processed and annotated data can be downloaded from https://www.ncbi.nlm.nih.gov/geo/query/acc.cgi?acc=GSE84133. The data is in log2 scale. We grouped the cells into 958 acinar cells, 1077 ductal cells (Duct), 252 endothelial cells (EC), 5755 endocrine cells (endocrine), 87 immune cells (IC) and 457 stellate cells (stellate). The

expression reference was constructed with MSS= (5,3,5,3,5,5), which resulted in 822 markers. The expression reference matrix was validated in a human pancreas Smart-Seq2 dataset from Enge et al[11], which can be downloaded from https://www.ncbi.nlm.nih.gov/geo/query/acc.cgi?acc=GSE81547. The cells were annotated by the authors and we normalized the TPM values by library size and log2-transformed with pseudocount +1.

Kidney: The kidney expression reference matrix was constructed with a human kidney scRNA-seq data from Muto et al[12]. This is a human kidney 10X dataset. The processed and annotated data can be downloaded from https://cellxgene.cziscience.com/collections/9b02383a-9358-4f0f-9795-a891ec523bcc. We grouped the cells into 1008 endothelial cells (EC), 18468 epithelial cells (Epi), 207 fibroblasts (Fib) and 63 immune cells (IC). The expression reference matrix was constructed with MSS= (3,2,2,2), which resulted in 272 markers. The expression reference matrix was validated in a human kidney 10X dataset from Wu et al[13], which can be downloaded from https://www.ncbi.nlm.nih.gov/geo/query/acc.cgi?acc=GSE109564. We processed it with Seurat3.0 pipeline[3] using the same parameter choice from paper. We retained the cells with >300 and <4000 genes expressed and mitochondrial contents<30%. *NormalizeData* function was used to log-normalize the data with scaling factor 10000. After data normalization, we identified highly variable genes as mean expression between 0.0125 and 6, and dispersion>1. After scaling of the data, we used first 20 principal components from PCA to do tSNE dimensionality reduction. *FindNeighbors* and *FindClusters* functions were used to identify 16 clusters with resolution=0.6. We classified the cells into 4 cell types with canonical marker gene expression from the same paper[13], which are 521 endothelial cells (EC), 1748 epithelial cells (Epi), 292 fibroblasts (Fib) and 1715 immune cells (IC).

Prostate: The prostate expression reference matrix was constructed with a human prostate 10X scRNA-seq dataset from Henry et al [14]. The dataset can be downloaded from https://www.ncbi.nlm.nih.gov/geo/query/acc.cgi?acc=GSE117403. We processed it with Seurat3.0 pipeline[3] using the same parameter choice from paper. We retained the cells with >500 and <3000 genes expressed and mitochondrial contents<10%. *NormalizeData* function was used to log-normalize the data with scaling factor 10000. Cell cycle state was predicted with *CellCycleScoring* function. The highly variable genes are identified as mean expression between 0.2 and 5, and dispersion>1. After scaling and regressing out variation in total UMI, percent mitochondrial genes and cell cycle phase, we used first 30 principal components from PCA to do tSNE dimensionality reduction. *FindNeighbors* and *FindClusters* functions were used to identify 14 clusters with resolution=0.6. We classified the cells into 6 cell types with canonical marker gene expression from the same paper[14]. We used patient 3 sample to construct the reference and patient 1&2 samples to validate the expression reference. After annotation, the patient 3 sample contains 5000 basal cells (Basal), 1702 endothelial cells (EC), 1372 fibroblasts (Fib), 2669 luminal cells (Luminal), 331 immune cells (IC) and 1174 smooth muscle cells (SMC). The patient 1&2 samples have in total 26216 basal cells (Basal), 2335 endothelial cells (EC), 11946 fibroblasts (Fib), 7654 luminal cells (Luminal), and 8614 smooth muscle cells (SMC). The expression reference matrix was constructed with MSS= (3,3,3,3,3,3), which resulted in 680 markers.

Breast: The mammary gland expression reference matrix was constructed with Tabula Muris data [9]. We combined the 10X mammary gland data and the Smart-Seq2 subcutaneous fat tissue data, for the 10X mammary gland data failed to profile fat cells. There are 392 basal cells (Basal), 251 endothelial cells (EC), 1678 fat cells (Fat), 700 fibroblast (Fib), 459 luminal cells (Luminal), 2493 lymphocytes (Lym) and 186 macrophages (Macro) in the combined data. The Seurat objects can be downloaded from https://tabula-muris.ds.czbiohub.org. The data is in log scale, and no additional normalization is made when combining the two datasets. The reference matrix was constructed running EpiSCORE with MSS= (3,3,6,3,3,3,3), which resulted in 2907 markers. Mouse genes were transformed into homologous human genes with ENSEMBL database. The expression reference matrix was validated in Tabula Muris Smart-Seq2 mammary gland data. The processing steps are the same as described previously.

Colon: The colon expression reference matrix was constructed with human large intestine 10X single cell RNA-seq data from Elmentaite et al[15], which can be downloaded from https://www.gutcellatlas.org/. The data were provided with UMI counts and annotated with cell types by the authors. We selected cells collected from large intestine of healthy adult. We processed it with Seurat4.0 pipeline[16]. We retained the cells with >500 and <4000 genes expressed and mitochondrial contents<20%. Genes were filtered for expression in more than 3 cells. We log-normalized the data with scaling factor 10000. There are 1599 endothelial cell (EC), 17504 epithelial cells (Epi), 13814 lymphocytes (Lym), 681 myeloid (Mye) and 3104 stromal cells remaining in the dataset. An expression reference matrix was constructed with MSS= (4,4,3,4,4), which resulted in 381 markers. The expression reference matrix was validated in HCL colon and rectum data. The processing steps are the same as described previously.

Heart: The heart expression reference matrix was constructed with a human heart droplet-based scRNA-seq dataset from Wang et al [17], which can be downloaded from https://www.ncbi.nlm.nih.gov/geo/query/acc.cgi?acc=GSE109816. Cell type annotation was provided by author. The UMIs were normalized by library size and then log2-transformed with pseudocount +1. There are 3393 cardiac muscle cells (CM), 1946 endothelial cells (EC), 406 fibroblast (Fib), 286 macrophages (Macro), 700 smooth muscle cells (SMC) in the dataset. The expression reference matrix was constructed with MSS= (4,2,4,2,2), which resulted in 1180 markers, and validated in the Tabula Muris 10X Heart and Aorta data [9]. The processing steps are the same as described previously.

Brain: The brain expression reference matrix was constructed with a 10X snRNA-seq dataset from Li et al [18]. Data was downloaded from http://development.psychencode.org/. The cell type annotation and data normalization were provided by the author. There are 8 cell types from adult human prefrontal cortex, which included 12678 neurons, 2078 astrocytes (Astro), 899 oligodendrocytes (Oligo), 464 endothelial cells (Endo), 130 microglia, and 810 oligodendrocyte progenitor cells (OPC). The expression reference matrix was constructed with EpiSCORE using MSS= (5,4,4,3,5,3), which resulted in 1243 markers, and validated in a Smart-Seq adult human brain dataset from Darmanis et al [19]

(https://www.ncbi.nlm.nih.gov/geo/query/acc.cgi?acc=GSE67835). The cell type annotation was provided by author and we normalized the validation dataset by library size and log2-transformed with pseudocount +1.

Esophagus: The esophagus expression reference matrix was constructed with a human esophagus 10X dataset from Madissoon et al [20]. The processed Seurat object was downloaded from https://www.tissuestabilitycellatlas.org/. We have 1542 endothelial cells (EC), 8916 basal epithelial cells (Epi_basal), 10000 stratified epithelial cells (Epi_stratified, after downsampling), 8736 suprabasal epithelial cells (Epi_suprabasal), 9094 upper epithelial cells (Epi_upper), 537 fibroblasts, 868 gland cells and 3267 immune cells (IC). The expression reference matrix was constructed running EpiSCORE with MSS= (5,4,5,4,5,5,4,4), which resulted in 1227 markers. The expression reference matrix was validated in HCL Microwell-Seq esophagus dataset [4] (https://figshare.com/articles/dataset/HCL_DGE_Data/7235471). The cell types were annotated by author. The counts were divided by the total counts within the cell and then multiplied by the maximum total count across all cells. Then they were log2-transformed with pseudocount +1. According to canonical marker gene expression from literatures, we re-annotated the cells in HCL. The KRT13 high and KRT14 high cells were treated as basal cells. The KRT17 high, KRT7 high and MMP7 high cells were treated as gland cells. The KRT4 high, KRT16 high and keratinocytes were treated as stratified epithelial cells.

Construction of reference matrices from Human Cell Landscape

The quality control processed HCL Microwell-Seq data [4] can be downloaded from https://figshare.com/articles/dataset/HCL_DGE_Data/7235471 . The cell type annotation is provided by the author. Counts were normalized as in the Seurat algorithm, but no cells were removed as all of these already passed QC.

Brain: The brain expression reference matrix was constructed from the HCL adult cerebellum sample. In this dataset there are 1776 astrocytes (Astro), 159 endothelial cells (EC), 1736 microglia, 304 neurons, 2346 oligodendrocytes (Oligo), 291 oligodendrocyte progenitor cells (OPC). When applying the EpiSCORE procedure to build the reference matrices, even when using the least stringent MSS, the mRNA expression reference matrix only contained a few neuronal markers, resulting in no neuronal markers for the DNAm reference matrix. Thus, we relaxed the marker selection procedure from the scRNA-Seq data, not using medians and not counting contrasting cell-types where the median value is zero. Instead, we used the log fold change of mean expression: for one cell type, we compared the log FC of mean expression to each of the other cell types. Then we selected genes with the maximum mean expression level in that cell type and with logFC>0.3 when compared to at least 3 other cell types. The marker gene should also pass FDR<0.05 in Wilcoxon test between one cell type against all the other cell types. This resulted in an expression reference matrix with 264 Astro, 126 EC, 254 Microglia, 131 Neuron, 359 Oligo and 102 OPC expression markers (total N=1236). For the imputed DNAm reference matrix, there were 39 Astro, 15 EC, 59 Microglia, 9 Neuron, 39 Oligo and 17 OPC markers (a total of N=178 markers).

Heart: The heart expression reference matrix was constructed with 2 adult heart HCL samples. In this dataset, there are 201 cardiac muscle cells (CM), 329 endothelial cells (EC), 490

fibroblast (FB), 630 macrophages (MP) and 492 smooth muscle cells (SMC). The expression reference matrix was constructed with EpiSCORE using MSS = (2,2,2,2,2), which resulted in 32 CM, 13 EC, 27 FB, 37 MP and 47 SMC markers (N=156). The imputed DNAm reference matrix contains 3 CM, 5 EC, 11 FB, 10 MP and 9 SMC markers (N=38).

References

1. Travaglini, K.J. *et al.* A molecular cell atlas of the human lung from single-cell RNA sequencing. *Nature* **587**, 619-625 (2020).
2. Yu, Z. *et al.* Single-Cell Transcriptomic Map of the Human and Mouse Bladders. *J Am Soc Nephrol* **30**, 2159-2176 (2019).
3. Stuart, T. *et al.* Comprehensive Integration of Single-Cell Data. *Cell* **177**, 1888-1902 e21 (2019).
4. Han, X. *et al.* Construction of a human cell landscape at single-cell level. *Nature* **581**, 303-309 (2020).
5. MacParland, S.A. *et al.* Single cell RNA sequencing of human liver reveals distinct intrahepatic macrophage populations. *Nat Commun* **9**, 4383 (2018).
6. Aizarani, N. *et al.* A human liver cell atlas reveals heterogeneity and epithelial progenitors. *Nature* **572**, 199-204 (2019).
7. Durante, M.A. *et al.* Single-cell analysis of olfactory neurogenesis and differentiation in adult humans. *Nature Neuroscience* **23**, 323-326 (2020).
8. Solé-Boldo, L. *et al.* Single-cell transcriptomes of the human skin reveal age-related loss of fibroblast priming. *Communications Biology* **3**, 188 (2020).
9. Schaum, N. *et al.* Single-cell transcriptomics of 20 mouse organs creates a Tabula Muris: The Tabula Muris Consortium. *Nature* **562**, 367 (2018).
10. Baron, M. *et al.* A Single-Cell Transcriptomic Map of the Human and Mouse Pancreas Reveals Inter- and Intra-cell Population Structure. *Cell Syst* **3**, 346-360 e4 (2016).
11. Enge, M. *et al.* Single-Cell Analysis of Human Pancreas Reveals Transcriptional Signatures of Aging and Somatic Mutation Patterns. *Cell* **171**, 321-330.e14 (2017).
12. Muto, Y. *et al.* Single cell transcriptional and chromatin accessibility profiling redefine cellular heterogeneity in the adult human kidney. *Nature Communications* **12**, 2190 (2021).
13. Wu, H. *et al.* Single-Cell Transcriptomics of a Human Kidney Allograft Biopsy Specimen Defines a Diverse Inflammatory Response. *J Am Soc Nephrol* **29**, 2069-2080 (2018).
14. Henry, G.H. *et al.* A Cellular Anatomy of the Normal Adult Human Prostate and Prostatic Urethra. *Cell Rep* **25**, 3530-3542.e5 (2018).
15. Elmentaite, R. *et al.* Cells of the human intestinal tract mapped across space and time. *Nature* **597**, 250-255 (2021).
16. Hao, Y. *et al.* Integrated analysis of multimodal single-cell data. *Cell* **184**, 3573-3587.e29 (2021).
17. Wang, L. *et al.* Single-cell reconstruction of the adult human heart during heart failure and recovery reveals the cellular landscape underlying cardiac function. *Nat Cell Biol* **22**, 108-119 (2020).
18. Li, M. *et al.* Integrative functional genomic analysis of human brain development and

neuropsychiatric risks. **362**, 1264 (2018).

19. Darmanis, S. *et al.* A survey of human brain transcriptome diversity at the single cell level. *Proc Natl Acad Sci U S A* **112**, 7285-90 (2015).

20. Madissoon, E. *et al.* scRNA-seq assessment of the human lung, spleen, and esophagus tissue stability after cold preservation. *Genome Biol* **21**, 1 (2019).
