# Supplementary material for: A pan-tissue DNA methylation atlas enables in silico decomposition of human tissue methylomes at cell-type resolution
Source: Nat Methods. 2022 Mar 11;19(3):296–306. doi: 10.1038/s41592-022-01412-7 (PMC8916958; doi:10.1038/s41592-022-01412-7)
Supplement: Supplementary file 2 — Reporting Summary [file 41592_2022_1412_MOESM2_ESM.pdf]

nature research

Corresponding author(s): Andrew E Teschendorff

Last updated by author(s): Jan 7, 2022

Reporting Summary

Nature Research wishes to improve the reproducibility of the work that we publish. This form provides structure for consistency and transparency in reporting. For further information on Nature Research policies, see our Editorial Policies and the Editorial Policy Checklist.

Statistics

For all statistical analyses, confirm that the following items are present in the figure legend, table legend, main text, or Methods section.

n/a	Confirmed	
☐	☒	The exact sample size (*n*) for each experimental group/condition, given as a discrete number and unit of measurement
☐	☒	A statement on whether measurements were taken from distinct samples or whether the same sample was measured repeatedly
☐	☒	The statistical test(s) used AND whether they are one- or two-sided<br>*Only common tests should be described solely by name; describe more complex techniques in the Methods section.*
☐	☒	A description of all covariates tested
☐	☒	A description of any assumptions or corrections, such as tests of normality and adjustment for multiple comparisons
☐	☒	A full description of the statistical parameters including central tendency (e.g. means) or other basic estimates (e.g. regression coefficient) AND variation (e.g. standard deviation) or associated estimates of uncertainty (e.g. confidence intervals)
☐	☒	For null hypothesis testing, the test statistic (e.g. F, t, r) with confidence intervals, effect sizes, degrees of freedom and P value noted<br>*Give P values as exact values whenever suitable.*
☒	☐	For Bayesian analysis, information on the choice of priors and Markov chain Monte Carlo settings
☒	☐	For hierarchical and complex designs, identification of the appropriate level for tests and full reporting of outcomes
☐	☒	Estimates of effect sizes (e.g. Cohen's *d*, Pearson's *r*), indicating how they were calculated

Our web collection on statistics for biologists contains articles on many of the points above.

Software and code

Policy information about availability of computer code

Data collection	All data was downloaded directly from the respective websites as specified in the Data availability section. No software was used to download data.
Data analysis	EpiSCORE version 0.9.4 is freely available as an R-package from figshare (https://figshare.com/articles/software/EpiSCORE_R_package/14401340), from github (https://github.com/aet21/EpiSCORE) under a GPL-2 licence, or from zenodo (https://doi.org/10.5281/zenodo.3893646) under a Creative Commons Attribution 4.0 International Public License ("Public License"). The R-package comes with a vignette and tutorial, sample datasets and a reference manual. A capsule demonstrating a simple use of the DNAm-atlas reproducing some of the results for liver-tissue is available on CodeOcean (http://codeocean.com) via the provisional DOI 10.24433/CO.8741744.v1 . Other software packages used and their versions include R-packages mixtools_1.2.0 , MASS_7.3-54 , impute_1.66.0 , SuperExactTest_1.0.7 , minfi_1.38.0 , EpiDISH_2.8.0 , RcisTarget_1.12.0

For manuscripts utilizing custom algorithms or software that are central to the research but not yet described in published literature, software must be made available to editors and reviewers. We strongly encourage code deposition in a community repository (e.g. GitHub). See the Nature Research guidelines for submitting code & software for further information.

Data

Policy information about availability of data

All manuscripts must include a data availability statement. This statement should provide the following information, where applicable:

- Accession codes, unique identifiers, or web links for publicly available datasets
- A list of figures that have associated raw data
- A description of any restrictions on data availability

The DNAm datasets analyzed in this manuscript are all publicly available from the respective publications or from GEO (www.ncbi.nlm.nih.gov/geo/) under the

following accession numbers: GSE123995 (hepatocytes), GSE49656 (cholangiocarcinoma), GSE122126, GSE143209 and GSE124809 (pancreatic cell-types), GSE84274 (human aorta samples), GSE130711 (human prefrontal cortex snmC-seq2), GSE66351 (human frontal cortex 450k), GSE51954 (human dermis & epidermis). The human purified dorsolateral prefrontal cortex 450k dataset from Guintivano et al is available from FlowSorted.DLPFC.450k Bioconductor package. The TCGA Illumina 450k datasets for LUAD, LUSC, BLCA, LIHC, CHOL, SKCM, PAAD, KICH, KIRC, KIRP, PRAD, BRCA, COAD, READ, GBM, LGG, ESCA datasets are available from the GDC data portal https://portal.gdc.cancer.gov/. The DNAm-atlas, including all the mRNA and DNAm reference matrices for the 13 tissue-types, is published as a resource on figshare (https://figshare.com/projects/EpiSCORE-atlas_version-1_/111473). The datafile hg19-500bp-upstream-7species.mc9nr.feather, which contains the motif rankings for regions 500bp upstream of the TSS of 22284 genes across 7 species is available online (https://resources.aertslab.org/cistarget/ . Source Data for each main figure has been provided in excel spreadsheets labelled with their respective figure-number. All source data excel files are in the zip file labelled "SourceData.zip

Field-specific reporting

Please select the one below that is the best fit for your research. If you are not sure, read the appropriate sections before making your selection.

☒ Life sciences ☐ Behavioural & social sciences ☐ Ecological, evolutionary & environmental sciences

For a reference copy of the document with all sections, see nature.com/documents/nr-reporting-summary-flat.pdf

Life sciences study design

All studies must disclose on these points even when the disclosure is negative.

Sample size	In this work we only analyze publicly available datasets, and therefore we are restricted to the sample-sizes as provided by the published studies. For the purpose of this work, sample sizes were in general large enough to achieve statistical significance in our analyses.
Data exclusions	In general no samples were excluded. Probes or CpG measurements that did not pass quality control were removed as described in Methods and in the respective publications where the DNA methylation datasets were generated.
Replication	The mRNA expression reference matrices were all validated in independent single-cell RNA-Seq datasets. The DNAm reference matrices and the cell-type fractions derived from them are validated in independent DNA methylation datasets. Number of replications/validations is limited by dataset availability and ranged from 1 to 3.
Randomization	This study does not generate new data, hence not applicable.
Blinding	This study does not generate new data. Phenotypic information was downloaded alongside the mRNA and DNAm datasets, and used in the final analyses.

Reporting for specific materials, systems and methods

We require information from authors about some types of materials, experimental systems and methods used in many studies. Here, indicate whether each material, system or method listed is relevant to your study. If you are not sure if a list item applies to your research, read the appropriate section before selecting a response.

Materials & experimental systems

n/a	Involved in the study	
☒	☐	Antibodies
☒	☐	Eukaryotic cell lines
☒	☐	Palaeontology and archaeology
☒	☐	Animals and other organisms
☒	☐	Human research participants
☒	☐	Clinical data
☒	☐	Dual use research of concern

Methods

n/a	Involved in the study	
☒	☐	ChIP-seq
☒	☐	Flow cytometry
☒	☐	MRI-based neuroimaging
