# Supplementary material for: A pan-tissue DNA methylation atlas enables in silico decomposition of human tissue methylomes at cell-type resolution
Source: Nat Methods. 2022 Mar 11;19(3):296–306. doi: 10.1038/s41592-022-01412-7 (PMC8916958; doi:10.1038/s41592-022-01412-7)
Supplement: Supplementary file 5 — Readme file and example R-script+ data to run example. [file 41592_2022_1412_MOESM5_ESM.zip › README.docx]

This README file describes basic features of the **EpiSCORE R-package and the DNAm-atlas resource.** The up-to-date version of EpiSCORE (current version is 0.9.2) will always be available from <https://github.com/aet21/EpiSCORE> . Version 0.9.2 is available from <https://figshare.com/articles/software/EpiSCORE_R_package/14401340> . The DNAm-atlas resource is available from <https://figshare.com/projects/EpiSCORE-atlas_version-1_/111473> .

1. **System Requirements:**

Both the DNAm-atlas resource and the EpiSCORE R-package are designed to be used with the open-source statistical programming environment “R” (<http://cran.r-project.org>), version 3.6 or higher. The DNAm-atlas resource consists of .Rda files which contain the tissue-specific mRNA and DNAm reference matrices for 13 tissue-types. For convenience, these matrices are also provided as .csv files. The EpiSCORE package has the following R-package dependencies: org.Hs.eg.db, AnnotationDbi, MASS, parallel, EpiDISH and presto. All these packages except presto can be downloaded via the BiocManager::install() command from the Bioconductor R website (<http://www.bioconductor.org> ). The “presto” R-package needs to be installed via github and is available from <https://github.com/immunogenomics/presto> . EpiSCORE can be run on most linux-systems, MAC-OSX and Windows. We have tested EpiSCORE on Ubuntu 18.04.5 LTS and CentOS Linux 7, and with R_3.6 and R_4.1.0. For end-user applications, we recommend at least 16GB RAM, and access to a machine with parallel computing facilities (>4 processing cores).

1. **Installation Guide:**

From your R-session:

*> library(devtools)*

*> install_github(“immunogenomics/presto”)*

*> install_github(“aet21/EPISCORE”)*

Installation time is on the order of seconds.

1. **Demo**

A full demonstration including vignette and tutorial of how to run EpiSCORE is provided with the R-package upon installation. The examples run in the tutorial illustrate the basic functionality of the package in building and validating a tissue-specific mRNA expression reference matrix, the imputation of a corresponding tissue-specific DNAm reference matrix, and subsequent application of this DNAm reference matrix to estimate cell-type fractions and infer cell-type specific differential DNAm. The total runtime for running the tutorial in the vignette is approximately 1 minute on a standard workstation.

1. **Instructions for use**

Below is pseudocode for using the DNAm-atlas resource. For instance, in the case of skin, we would download the R-object data file for skin from the figshare site given above, and load it into R using the command

- *load(“SkinRef.rda”)*

Upon loading this data object, there are two reference matrices, one for gene-expression (*Skin_Expref.m*) and another for DNA methylation (*Skin_Mref.m*). The DNAm reference matrix for skin is defined for 111 marker genes and 7 cell-types which include endothelial cells (EC), fibroblasts (Fib), differentiated and undifferentiated keratinocytes (Kera_diff & Kera_undiff), macrophages (Macro), melanocytes (Mela) and T-cells (Tcells). To validate the DNAm reference matrix, we can apply it to an Illumina 450k DNAm skin dataset with samples from dermis and epidermis, since the epidermis contains mostly keratinocytes whilst the dermis is mostly composed of fibroblasts and endothelial cells. A version of this dataset where DNAm is summarized at the gene-level (NCBI EntrezIDs), is provided, so we first load it in using

- load(“VandiverDNAm.rda”)

The next step is to then estimate cell-type fractions in this data, which can be accomplished using the *wRPC* function of the EpiSCORE R-package:

- *estF.o <- wRPC(VandiverDNAm.m,ref=Skin_Mref.m,useW=TRUE,wth=0.4,maxit=200)*

In the above, the parameter *useW=TRUE*, means that we are going to use the weights (a quality/confidence score for each marker gene) when performing the multivariate regression, and the threshold *wth=0.4*, specifies that we will only use marker genes with weights larger than 0.4 when performing the weighted regression. The last parameter *maxit*  specifies the maximum number of iterations to perform.

The estimated cell-type fraction matrix, i.e. a matrix with rows labeling the samples and columns labeling the 7 cell-types can be found in the output object as *estF.o$est.* Finally, one would check that the fractions for endothelial and fibroblast cells are highest in the dermis, whilst the fractions for the keratinocytes are highest in the epidermis. Meta-information regarding the skin samples, including dermis/epidermis, is provided in the *PhenoTypesVAND.lv$Tissue* object.

In addition, we also provide an Ocean Code Capsule demonstrating a simple use of the DNAm-atlas, reproducing some of the results for liver-tissue. This capsule should be accessible on OceanCode (http://codeocean.com ) via the provisional DOI 10.24433/CO.8741744.v1 . Of note, there is a brief delay between assignment of the DOI and publication of the capsule. Editors may wish to contact OceanCode directly to gain access to the capsule with the above DOI.
