# Supplementary material for: A pan-tissue DNA methylation atlas enables in silico decomposition of human tissue methylomes at cell-type resolution
Source: Nat Methods. 2022 Mar 11;19(3):296–306. doi: 10.1038/s41592-022-01412-7 (PMC8916958; doi:10.1038/s41592-022-01412-7)
Supplement: Supplementary file 3 — Peer Review Information [file 41592_2022_1412_MOESM3_ESM.pdf]

Peer Review Information

Journal: Nature Methods
Manuscript Title: A pan-tissue DNA methylation atlas enables in-silico decomposition of human tissue methylomes at cell-type resolution
Corresponding author name(s): Charles E. Breeze, Andrew E. Teschendorff

Reviewer Comments & Decisions:

Decision Letter, initial version:

Subject: Decision on Nature Methods submission NMETH- RS46537A-Z
Message:

20th Sep 2021

Dear Professor Teschendorff,

Your Resource entitled "A pan-tissue DNA methylation atlas enables in-silico microdissection of human tissue methylomes at cell-type resolution" has now been seen by 3 reviewers, whose comments are attached. While they find your work of some potential interest, they have raised seriously technical concerns which in our view are sufficiently important that they preclude publication of the work in Nature Methods.

That being said, we are willing to consider a revised manuscript if further data allow you to address all the major criticisms of the reviewers (unless, of course, something similar has by then been accepted at Nature Methods or appeared elsewhere). This includes submission or publication of a portion of this work somewhere else.

The required new experiments and data include, but are not limited to, additional validations of the DNA methylation atlas as well as demonstrations showing that the use of such methylation atlas has advantages than that of a single large scRNA-seq dataset. We hope you understand that until we have read the revised paper in its entirety we cannot promise that it will be sent back for peer-review.

If you are interested in revising this manuscript for submission to Nature Methods in the future, please email me about your appeal before making any revisions. Otherwise, we hope that you find the reviewers' comments helpful when preparing your paper for submission elsewhere.

Sincerely,
Lei

Lei Tang, Ph.D.
Senior Editor
Nature Methods

Reviewers' Comments:

Reviewer #1:
Remarks to the Author:
In the manuscript entitled "A pan-tissue DNA methylation atlas enables in-silico microdissection of human tissue methylomes at cell-type solution" Zhu et al., describe and extensively validate a DNA methylation atlas that facilitates the cellular deconvolution of 13 different solid-tissue types. This atlas is essentially a collection of DNA methylation reference matrices, the necessary substrate for reference-based deconvolution, and allow one to predict the fractions of the cellular components of heterogenous biospecimens, provided that the biospecimen used for DNA methylation profiling is one of the 13 solid-tissue types in the atlas. The atlas described and presented in this manuscript is logical extension of a recently published method from this group called EpiSCORE (essentially, the methodological/computational approach for creating DNA methylation reference matrices using a combination of single cell RNAseq data and paired mRNA and DNA methylation data), but expands the repertoire of tissue types amenable for deconvolution beyond what were considered in the original EpiSCORE paper. One of the fundamental challenges facing EWAS with bulk-tissue methylation data lies in the interpretation of differentially CpGs derived from such data as bulk-tissue is comprised of a myriad of different cell types, each with their own unique methylation signature. Thus, to obtain a more complete understanding of the biological processes underlying some disease, one needs to look at how DNA methylation is altered within specific cell populations. This fact is now widely recognized within the epigenomics research community. The atlas described here has the potential to facilitate such analyses when used in tandem with recently published methodologies that allow for cell-specific analyses of bulk-tissue methylation data (e.g., cellDMC, TCA, etc.) and therefore, fills a critical niche among methods/tools for analysis array-based DNA methylation collected in the context of large-scale epidemiological studies. The described atlas was validated using a number of publicly available data sets from different solid tissue types (e.g., liver, brain, pancreas, skin, etc.), which all showcased some of the

novel insights that can be gleaned from such a resource. This reviewer is very enthusiastic about the atlas described in this manuscript and the potential avenues it could open up within the epigenomics research community. That said, there are certain points that I would like the authors to address as I feel they could strengthen the manuscript at the corresponding DNA methylation atlas:

1. The one major point that deserves some discussion/consideration is that all of the DNA methylation reference matrices are based on the now discontinued Illumina HumanMethylation450 array (aka 450K array). This by no means invalidates or diminishes the contributions of this work as there a great many publicly available 450K methylation data sets consisting of DNA methylation profiled in the solid tissue types that comprise the provided atlas, however the community would be best served if reference matrices based on the EPIC array were also provided. Recognizing the extent of work that such would entail, it would be sufficient to demonstrate that the 450K reference matrices comprising the existing atlas are sufficient to enable accurate and reproducible deconvolution estimates when applied to a EPIC data set. Since ~90% of the CpGs on the 450K array are also contained on the EPIC array, chances are that the majority of CpGs in the existing reference sets are also on the EPIC array and thus, one could simply use the overlapping CpGs to do the deconvolution. The concern that I have is that some of the reference sets are rather small in terms of the number of CpGs relative to the number of cell types being deconvoluted and my fear is that removing even a small number of CpGs from the reference matrix might throw off the deconvolution estimates. Another consideration regarding the EPIC array is that since it has twice the coverage of the 450K array, might it be possible to construct reference matrices based on the EPIC array that improve the accuracy of deconvolution estimates beyond that which can be obtained considering only 450K probes?

2. This might be beyond the scope of the current paper as this comment is less about the atlas itself and more about how this resource fits into the broader context of other recent methodological developments for bulk-tissue methylation data (e.g., cellDMC, TCA, etc.), but I think it would be extremely valuable to cross-compare cell-specific methylation events/effects identified using this atlas in tandem with cellDMC/TCA, when applied to bulk-tissue methylation data in a solid tissue type, to the results of single cell methylation data in the same tissue type. For example, suppose we have 450K methylation data in liver collected from healthy and diseased subjects. If there exists single cell methylation data in the same tissue type for the same biological conditions (doesn't necessarily need to be the exact same subjects with 450K data), are the results for cell-specific methylation effects consistent? Has this been done before?

3. Another question I had is that the authors only consider CpGs that are both proximal to the TSS of marker genes and strongly anticorrelated with the expression of those genes, to build DNA methylation reference libraries. While I understand and completely agree with the second condition (e.g., that the CpG site must exhibit a strong correlation with expression), otherwise the proposed imputation procedure falls apart, why restrict only to CpGs that are proximal to the TSS of the gene? Why not just

consider CpGs that a located in/near the gene of interest and that exhibit strong correlation with gene expression? Seems like there might be a missed opportunity here in building more robust DNA methylation libraries, but perhaps I'm missing something.

4. The final point that I have is that the Discussion section doesn't explicitly acknowledge the limitations of this study. No study, no method, no resource, etc., is without its limitations and I feel that it is important for the authors to shed light on the limitations of their resource and the study as a whole.

Reviewer #2:
Remarks to the Author:
DNA methylation is being studied extensively as a potential biomarker for human diseases, and one of the most important problems is how to account for cell type composition within bulk tissue samples. The Teschenforff group has developed several mathematical models for reference-based deconvolution of DNA methylation data, including CellDMC which identifies disease-associated methylation within a specific cell type in the mixture, and EPISCORE, which exploits the high resolution cell type clusters available from single-cell RNA sequencing to impute cell-type specific DNA methylation values. These are important tools, given the general lack of availability of DNA methylation reference data derived from either single-cell or FACS-sorted methylation sequencing.

The Teschendorff group published the EPISCORE algorithm in Teschendorff et al. 2020 (Genome Biology), and validated for lung and breast cell types. They also showed how it could be combined with CellDMC to identify cell type specific differences in bulk lung tumor tissue. This new manuscript creates similar imputed reference atlases for 11 additional tissue types, developing a pipeline for collecting scRNA-seq data and validating cell type clusters, then imputing DNA methylation references. They validate their deconvolution estimates using non-methylation based deconvolution methods on TCGA tumor data, as well as methylation profiles of FACS-sorted samples from individual studies of tissues of healthy individuals. They use this new atlas with EPISCORE and CellDMC make several interesting findings about cell type composition and DNA methylation changes in melanoma, olfactory neuroblastoma, and schizophrenia.

While most of the elements of this work were already present in the 2020 Genome Biology paper, this new manuscript represents a significant amount of work to standardize and validate the process of collecting additional cell types. Additionally, all the data and R code are publicly available on github and figshare, making this a useful resource. My main concerns are about several tissue types that perform poorly, and how that should be handled in the quality control pipeline.

The manuscript was clear and properly referenced.

Major concerns.

My major concern has to do with quality control, since the tissue scRNA-seq data used to build the atlas comes from diverse sources and sequencing techniques, which may present consistency problems for the framework. Here are two examples:

- The main systematic validation uses TCGA multi-omic data to compare EPISCORE to other methods of deconvolution (most importantly, RNA-seq based) in Fig 2a-b. The KICH cancer type is the main outlier, which has poor correlation between EPISCORE and all other deconvolution methods. In looking at the Kidney cell types in the imputed Atlas (Fig S7), it does look like kidney has a relatively small number of features, and a lot of confusion between cell types. Should this tissue type be excluded due to low quality? Should you implement minimum quality standards so that users don't use poor models?

- The gold standard validation is against FACS-sorted cell types profiled using the DNA methylation Infinium array. The authors provide four such validations - hepatocytes in liver, neuron vs. glia in brain tissue, Dermis vs epidermis in skin, and endocrine vs. epithelial cell types in pancreas. EPISCORE performs relatively well on the first three , which are shown in main figures, but performs poorly on the 4th, which is shown only as a supplemental figure. Despite the fact that all QC metrics appear to look fine for the pancreatic scRNA-seq clusters and imputation (Fig S6), there is significant confusion between ductal and endocrine cells in multiple FACS-sorted samples from different sources, and confusion between acinar and ductal cells in another (Fig S17). So where is this problem coming from, is it the scRNA-seq data, the imputation method, or neither? Was it because the imputation was based on whole-genome data and the test data was on the array platform? Should this be used to omit the pancreatic model from the Atlas? This is important because the main value of this work is for the resource of tissue specific models provided and the reliability.

The issues with heterogeneity of underlying scRNA-seq data and different quality levels beg the question of whether this approach would be more reliable using a single large scRNA-seq dataset based on a common technology and analysis methods. Such a dataset became available from GTEx/Broad Institute just before (https://doi.org/10.1101/2021.07.19.452954). I realize this dataset came too late for you to analyze, but I think it's important to comment on how this will improve quality, and how quality of the underlying models can be better quantified/validated.

Minor

Melanoma is shown in Fig 2 and then again in Fig 6. It is confusing and would be better shown in a single figure/section

Reviewer #3:
Remarks to the Author:
In their manuscript, Zhu et al. present methodology for reference-based deconvolution of DNA methylation data as well as an atlas of deconvoluted DNA methylomes. The novelty of their approach is that reference DNA methylation profiles are not obtained experimentally, but imputed based on available resources of bulk or single-cell gene expression measurements. This has high significance for the field, as reference DNA methylation profiles of major cell types remain rather scarce, whereas the number of cell atlases that are based on single-cell transcriptomic assays is growing rapidly. Overall the proposed approach for atlas construction is original and is based on solid assumptions about strong association between expression levels of cell type-specific marker genes and DNA methylation levels of their promoters. Although the computational method EPISCORE underlying current work was published earlier (Teschendorff et al., Genome Biology, 2020), in the current manuscript the authors apply it to variety of datasets and complex biological systems to clearly demonstrate its benefits and provide a resource of imputed DNA reference matrices for subsequent studies. The authors creatively designed numerous validation experiments to prove the feasibility and biological meaningfulness of obtained results. Overall, the framework appears to generate robust cell type estimates in multiple tissues that potentially lead to novel disease-relevant insights. Provided source code and example analyses could be run and reproduced seamlessly. Nevertheless, in my opinion there are several major gaps in validation analyses summarised in several points below, in particular the absence of clear-cut comparison to an appropriate ground truth.

1) During the validation of the tissue-specific reference gene expression matrices the prediction accuracy in independent scRNA-seq datasets was sometimes rather low for certain tissues and cell types (e.g. skin). Could this ambiguity result in wrong or biased composition estimates and lead to erroneous findings? Can authors suggest an approach to measure and mitigate the impact of these inaccuracies?

2) For two independent scRNA-seq datasets used to construct reference gene expression matrices, what were the criteria to select the primary dataset for marker selection and the secondary for validation? Could authors perform a round of reciprocal construction-validation procedures and compare the obtained markers and cell type estimation accuracy to their original results?

3). While the validation of imputed DNA methylation reference matrices appears convincing, it is rather indirect. Would it be possible to validate the imputed matrices directly on SCM2 and RMAP datasets (or any other similar dataset with both data modalities, e.g. brain tissue data presented in the manuscript) by comparing them to respective ground truth matrices using correlation or other appropriate distance?

4) Despite that numerous validations of the proposed methodology have been performed, a direct and quantitative comparison to a plausible ground truth is missing. Could the authors use one of the tissues for which both, reference (sc)RNA-seq and DNA methylation data of the same cell types available, and compare the EPISCORE results to those of any of the standard reference-based deconvolution algorithms (e.g Houseman's method, EpiDISH etc)? The brain tissue data presented in the manuscript appear to be perfectly suitable for this validation. How do proportions of brain cell types in schizophrenia recovered with EPISCORE-imputed DNA methylation matrix compare to those obtained using e.g. the pseudo-bulk scnmC-seq-based reference and how consistent would downstream annotation and interpretation be? Another possibility would be to use data obtained with one of the multi-modal single-cell protocols yielding expression and methylation data from the same cell (e.g. mouse gastrulation atlas in Argelaguet, Nature, 2019).

5) Although not explicitly emphasized, there is no obvious reason why similar atlases cannot be constructed based on bulk gene expression data, either array- or RNA-seq-based. In case it is indeed possible, rich reference epigenome resources (e.g. BLUEPRINT project for hematopoietic cells) can be used to perform a direct validation as described above.

6) What are the reasons for applying the proposed framework separate to each tissue? Would it be possible to impute DNA methylation matrices across multiple tissues and use them to estimate cell type proportions in samples of unknown origin? Could the authors test this?

On the minor side, which is however quite important for the statistical soundness of the obtained conclusions, I wonder whether a proportion test perhaps be more suitable for testing the differences of recovered cell type fractions as compared to the Wilcoxon rank sum test used for many analyses throughout the manuscript?

Last but not leasrt, the manuscript is written very clearly, with high accuracy in details, comprehensive and well-readable graphics. Relevant work is referenced appropriately.

** For Nature Research Group general information and news for authors, see http://npg.nature.com/authors.

Author Rebuttal to Initial comments

Detailed Response to Reviewers' Comments:

Reviewer #1:

General Comment: One of the fundamental challenges facing EWAS with bulk-tissue methylation data lies in the interpretation of differentially CpGs derived from such data as bulk-tissue is comprised of a myriad of different cell types, each with their own unique methylation signature. Thus, to obtain a more complete understanding of the biological processes underlying some disease, one needs to look at how DNA methylation is altered within specific cell populations. This fact is now widely recognized within the epigenomics research community. The atlas described here has the potential to facilitate such analyses when used in tandem with recently published methodologies that allow for cell-specific analyses of bulk-tissue methylation data (e.g., cellDMC, TCA, etc.) and therefore, fills a critical niche among methods/tools for analysis array-based DNA methylation collected in the context of large-scale epidemiological studies. The described atlas was validated using a number of publicly available data sets from different solid tissue types (e.g., liver, brain, pancreas, skin, etc.), which all showcased some of the novel insights that can be gleaned from such a resource. This reviewer is very enthusiastic about the atlas described in this manuscript and the potential avenues it could open up within the epigenomics research community. That said, there are certain points that I would like the authors to address as I feel they could strengthen the manuscript at the corresponding DNA methylation atlas.

Response: We would like to thank the reviewer for taking time to read and evaluate our MS. We are delighted that the reviewer shares our enthusiasm for this work, and that he/she agrees with the value that the DNAm-atlas would bring to the eigenomics community.

Specific comments:

Comment 1. The one major point that deserves some discussion/consideration is that all of the DNA methylation reference matrices are based on the now discontinued Illumina HumanMethylation450 array (aka 450K array). This by no means invalidates or diminishes the contributions of this work as there a great many publicly available 450K methylation data sets consisting of DNA methylation profiled in the solid tissue types that comprise the provided atlas, however the community would be best served if reference matrices based on the EPIC array were also provided. Recognizing the extent of work that such would entail, it would be sufficient

to demonstrate that the 450K reference matrices comprising the existing atlas are sufficient to enable accurate and reproducible deconvolution estimates when applied to a EPIC data set. Since ~90% of the CpGs on the 450K array are also contained on the EPIC array, chances are that the majority of CpGs in the existing reference sets are also on the EPIC array and thus, one could simply use the overlapping CpGs to do the deconvolution. The concern that I have is that some of the reference sets are rather small in terms of the number of CpGs relative to the number of cell types being deconvoluted and my fear is that removing even a small number of CpGs from the reference matrix might throw off the deconvolution estimates. Another consideration regarding the EPIC array is that since it has twice the coverage of the 450K array, might it be possible to construct reference matrices based on the EPIC array that improve the accuracy of deconvolution estimates beyond that which can be obtained considering only 450K probes?

Response: We appreciate the reviewer's point but as we argue below this issue is not a major concern, because our DNAm reference matrices are not just derived from 450k data but also from WGBS data of the Epigenomics Roadmap. In fact, there seems to be some confusion as to how the DNAm reference matrices were built, since they are not directly generated from 450k or WGBS data: the DNAm reference matrices are generated by imputation from a corresponding tissue-specific scRNA-Seq reference matrix. The imputation step filters marker genes in the mRNA expression reference matrix for a subset of marker genes for which there is an anticorrelation between promoter DNAm and gene-expression, as assessed over two separate databases of matched DNAm and mRNA expression profiles: only one of these databases is 450k based (SCM2) whereas the one derived from the Epigenomics Roadmap is WGBS-based. When building the final DNAm reference matrix we did not take the intersection of the two filtered marker gene lists, but the *union*, which means that our DNAm reference matrices are *not* actually restricted to 450k data. Indeed, the reviewer should also bear in mind that our DNAm reference matrices are defined at the level of gene-promoters, so as long as there is one probe or one CpG with sufficient coverage in the gene-promoter, a DNAm-value can be assigned to that marker gene and the gene will not drop out from the analysis. In fact, although 90% of the 450k probes are present on the EPIC array, the relevant question would be, for how many of our anticorrelated (i.e imputable) genes (a total of 2810) there is an EPIC probe

mapping to its promoter (within 200bp of the TSS). We have computed this and the fraction is 69.1%. As a comparison, for the 450k array this fraction is 68.7%, so as the reviewer can see, the fractions are extremely similar and for the EPIC array it is even higher. Thus, applying our DNAm reference matrices to EPIC data would not be a limitation at all. The only limitation stems from the identification of imputable genes, since if we had a comparable database to SCM2 but with EPIC instead of 450k, we would then be able to have a marginally higher pool of imputable genes. It would not be significantly higher, since as mentioned earlier, our imputable gene list derives from the union of two separate analysis, one of which was done at the level of WGBS. **In response to the reviewer's point we intend to clarify these important points in Discussion.**

And to further alleviate the reviewer's concern, **we plan to perform additional validation analyses using the EPIC array.** For instance, we already have an EPIC dataset suitable for testing the skin DNAm reference matrix, since in this EPIC dataset they profiled 8 skin fibroblast samples. The result of applying our skin DNAm reference matrix to this dataset confirms that all 8 samples are classified as fibroblasts. For convenience, we display the figure below, **which we would plan to add as a new Suppl.Fig. to a revised version of the MS:**

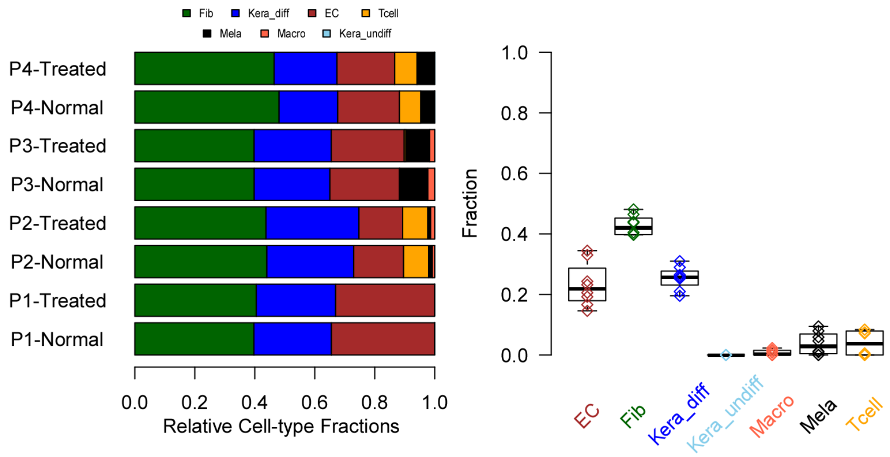

***Figure legend: Validation of skin DNAm reference in EPIC data of skin fibroblasts**. Left barplots display the estimated cell-type fractions using our skin DNAm reference matrix in each of 8 skin fibroblast samples. Boxplots to the right help the visual comparison between cell-types, confirming that all 8 samples would be classified as fibroblasts.*

Comment 2. This might be beyond the scope of the current paper as this comment is less about the atlas itself and more about how this resource fits into the broader context of other recent methodological developments for bulk-tissue methylation data (e.g., cellDMC, TCA, etc.), but I think it would be extremely valuable to cross-compare cell-specific methylation events/effects identified using this atlas in tandem with cellDMC/TCA, when applied to bulk-tissue methylation data in a solid tissue type, to the results of single cell methylation data in the same tissue type. For example, suppose we have 450K methylation data in liver collected from healthy and diseased subjects. If there exists single cell methylation data in the same tissue type for the same biological conditions (doesn't necessarily need to be the exact same subjects with 450K data), are the results for cell-specific methylation effects consistent? Has this been done before?

Response: We agree with the reviewer that in theory doing this would be a great way to validate the DNAm reference matrices, but of course the problem with this suggestion is that such data is not available for any of the solid tissue types (or not available in the sufficient numbers to reach solid conclusions). Generation of such data would also be extremely challenging technically and costly because one would have to generate genome-wide DNAm profiles for 100s of samples at the bulk-tissue level and then again for <u>all</u> cell-types in the tissue by FACS-sorting of these cell-types using surface markers that are imperfect or even controversial, so it is practically very difficult to do what the reviewer is suggesting. Moreover, the little single-cell methylomics data that is currently available is <u>very sparse</u>, suffering from low coverage. For instance, the snmC-Seq2 data matrix of brain cell-types that we have analysed in this work has 90% missing entries. Nevertheless, in response to the reviewer's point we have used the snmC-Seq2 data of brain cell subtypes to build a pseudo-bulk DNAm reference matrix, and have cross-compared estimated cell-type fractions from this reference with those of our EpiSCORE DNAm-atlas brain reference, in independent DNAm datasets (Gasparoni et al & Guintivano et al) profiling purified brain cell-types.

<u>asparoni et al:</u>

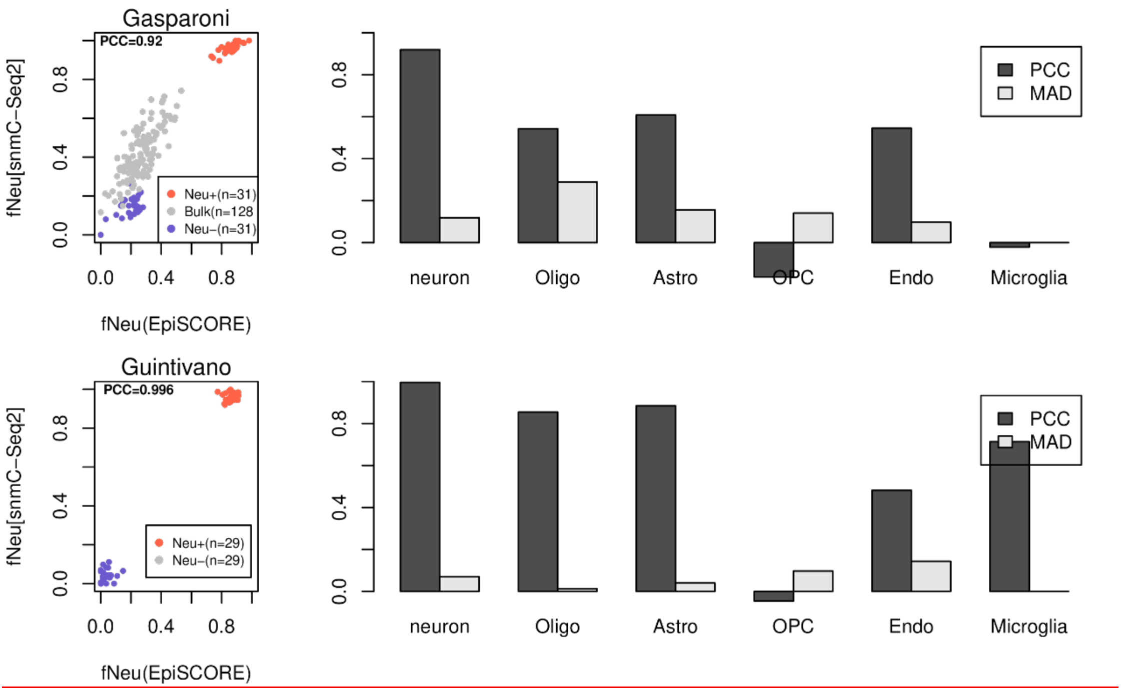

As we can see, there is excellent agreement, thus providing a strong validation. To the left we provide a scatterplot of the estimated neuronal fractions of both reference matrices in FACS sorted neuronal (Neu+) and non-neuronal (Neu-) populations (Gasparoni & Guintivano), as well as in bulk frontal cortex tissue (Gasparoni only). The Pearson Correlation Coefficients (PCC) are very high. To the right we display the Pearson Correlation Coefficient (PCC) and Median Absolute Deviation (MAD) between the snmC-Seq2 and EpiSCORE derived cell-type fractions for each brain cell-type. The most important measure to consider here is MAD, since for a cell-type that is only present in very low proportions, the corresponding PCC value is not expected to be large or may even be negative, even if the MAD between the two methods is excellent and very small. As we can see from both Guintivano and Gasparoni, the MAD is generally less than 0.2 and often also less than 0.1, suggesting good agreement of our EpiSCORE derived estimates with those from the snmC-Seq2 derived brain reference.

Comment 3. Another question I had is that the authors only consider CpGs that are both proximal to the TSS of marker genes and strongly anticorrelated with the expression of those genes, to build DNA methylation reference libraries. While I understand and completely agree

with the second condition (e.g., that the CpG site must exhibit a strong correlation with expression), otherwise the proposed imputation procedure falls apart, why restrict only to CpGs that are proximal to the TSS of the gene? Why not just consider CpGs that a located in/near the gene of interest and that exhibit strong correlation with gene expression? Seems like there might be a missed opportunity here in building more robust DNA methylation libraries, but perhaps I'm missing something.

Response: The reviewer has raised an excellent question. Among all probes/CpGs that map close to a gene, according to our analysis in Jiao Y, Teschendorff Bioinformatics 2014, the most predictive probes are those mapping close to the TSS. In fact, there is a substantially stronger association for these probes compared to probes mapping to the 5'UTR, further upstream from the TSS, or within the gene body, with only probes mapping to the 1st Exon demonstrating comparable strength. We did in fact explore using 1st Exon probes too, but results did not change markedly. What may lead to a significant improvement is to use probes/CpGs that map to enhancers linked to the gene in question, and indeed this is a question we already explored in our Genome Biol.2020 paper for the case of whole blood. In the case of whole blood there is a need to distinguish more similar cell-types from each other (e.g. CD4+ and CD8+ T-cells), and here we found that using enhancers in addition to promoters led to a significant improvement. However, blood is not the tissue where we need a tool like EpiSCORE since generating DNAm profiles for FACS-sorted purified cell-types is possible and has been done. In future, we would want to further improve the cellular resolution of the DNAm reference matrices built here. To this purpose, it is clear to us that probes/CpGs in the enhancer regions will need to be incorporated. However, the challenge here is that linking enhancers to genes is not trivial. We are planning a future improvement of the EpiSCORE method where we will do this, but this would clearly go beyond the scope of this MS and resource.

Comment: 4. The final point that I have is that the Discussion section doesn't explicitly acknowledge the limitations of this study. No study, no method, no resource, etc., is without its limitations and I feel that it is important for the authors to shed light on the limitations of their resource and the study as a whole.

Response: We absolutely agree that any resource and method has its limitations, and by no means did we imply that our resource does not have limitations. **In response to this point, we will include a paragraph discussing the current limitations of this resource.**

Reviewer #2:

General Comment: DNA methylation is being studied extensively as a potential biomarker for human diseases, and one of the most important problems is how to account for cell type composition within bulk tissue samples. The Teschenforff group has developed several mathematical models for reference-based deconvolution of DNA methylation data, including CellDMC which identifies disease-associated methylation within a specific cell type in the mixture, and EPISCORE, which exploits the high resolution cell type clusters available from single-cell RNA sequencing to impute cell-type specific DNA methylation values. These are important tools, given the general lack of availability of DNA methylation reference data derived from either single-cell or FACS-sorted methylation sequencing. The Teschendorff group published the EPISCORE algorithm in Teschendorff et al. 2020 (Genome Biology), and validated for lung and breast cell types. They also showed how it could be combined with CellDMC to identify cell type specific differences in bulk lung tumor tissue. This new manuscript creates similar imputed reference atlases for 11 additional tissue types, developing a pipeline for collecting scRNA-seq data and validating cell type clusters, then imputing DNA methylation references. They validate their deconvolution estimates using non-methylation based deconvolution methods on TCGA tumor data, as well as methylation profiles of FACS-sorted samples from individual studies of tissues of healthy individuals. They use this new atlas with EPISCORE and CellDMC make several interesting findings about cell type composition and DNA methylation changes in melanoma, olfactory neuroblastoma, and schizophrenia.

Response: We would like to thank the reviewer for taking time to read and evaluate our MS. We are delighted with the reviewer's positive feedback and for this reviewer to have recognized the value of the resource as well as many validations and interesting findings we have obtained

across a broad range of diseases including melanoma, olfactory neuroblastoma and schizophrenia.

Comment: While most of the elements of this work were already present in the 2020 Genome Biology paper, this new manuscript represents a significant amount of work to standardize and validate the process of collecting additional cell types. Additionally, all the data and R code are publicly available on github and figshare, making this a useful resource. My main concerns are about several tissue types that perform poorly, and how that should be handled in the quality control pipeline.

Response: We thank the reviewer for recognizing the important value that our DNAm-atlas resource will bring to the community. The reviewer is right in pointing out that for a few tissues (e.g. colon, kidney), the validation on the RNA-Seq side was not as strong as we would have liked. The purpose of displaying the validation accuracies is therefore to alert the user as to which of the downstream DNAm reference matrices would be most reliable. In response to the reviewer's point, we have now regenerated the expression reference matrices for kidney and colon using either new recently generated and higher quality scRNA-Seq atlases, or by lowering the cellular resolution (i.e. by combining highly similar cell-types into one broader cell-class). Validation accuracies are now much higher, as shown in the figures we provide below. In addition, we will provide a new Suppl.Table ranking the tissues by overall validation performance.

Comment: The manuscript was clear and properly referenced.

Response: We thank the reviewer for emphasizing this, as we did indeed put a lot of effort into it.

Major concerns.

Comment: My major concern has to do with quality control, since the tissue scRNA-seq data used to build the atlas comes from diverse sources and sequencing techniques, which may present consistency problems for the framework. Here are two examples:

- The main systematic validation uses TCGA multi-omic data to compare EPISCORE to other methods of deconvolution (most importantly, RNA-seq based) in Fig 2a-b. The KICH cancer type is the main outlier, which has poor correlation between EPISCORE and all other deconvolution methods. In looking at the Kidney cell types in the imputed Atlas (Fig S7), it does look like kidney has a relatively small number of features, and a lot of confusion between cell types. Should this tissue type be excluded due to low quality? Should you implement minimum quality standards so that users don't use poor models?

Response: We thank the reviewer for raising this good point. First of all, let us clarify that in Fig.2a-b, there are in fact 3 different kidney cancer types: KIRC, KIRP and KICH, and that for all 3 we are using the exact same kidney DNAm reference matrix. For KIRC and KIRP, validation is excellent, suggesting that the weaker validation in KICH is not necessarily a quality-issue of our kidney DNAm reference matrix. In fact, there are two other potential explanations why validation in KICH is weaker: the P-value for KICH would be expected to be less significant simply because the correlations were estimated across a relatively smaller number of samples (n=66). In contrast, the number of samples for KIRC (n=297) and KIRP (n=195) was much greater. A second potential reason is that KICH is a much rarer kidney tumor-type that exhibits substantial morphological variance, and so it could well be that our DNAm reference matrix is not applicable to this cancer-type, in the same way for instance that a lung DNAm reference matrix without a lung neuroendocrine cell-type would not be applicable to lung neuroendocrine cancers.

Nevertheless, we also agree with the reviewer that the relatively low validation accuracy of the kidney expression reference matrix is a concern. In response to this, we have now regenerated the kidney DNAm reference matrix at a lower cellular resolution (4 cell-types instead of previous 6), by defining a broader tubule epithelial cell class, and using this more coarse resolution reference matrix we obtain much better validation performance in the independent scRNA-Seq dataset, as shown in the figure below, which we intend to add to a revised Fig.S7:

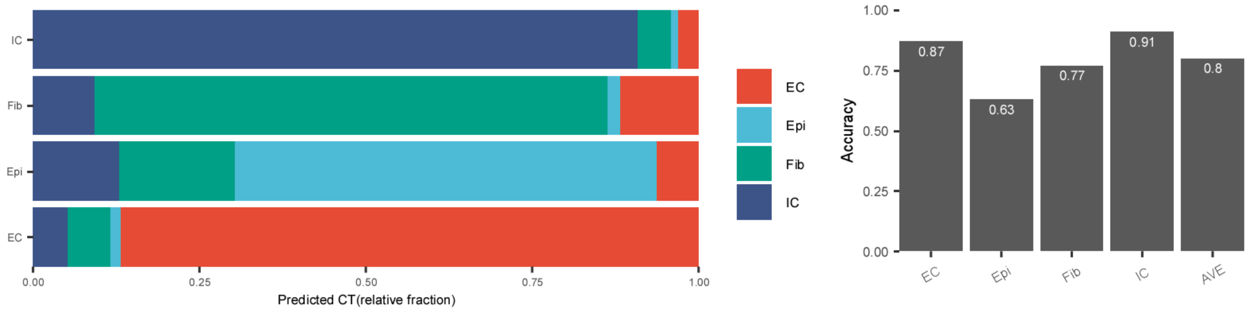

With this new kidney DNAm reference matrix, <u>the estimates of tumor purity and total immune-cell fractions in KICH also improve, as shown in a new Fig.2a-b, which for convenience we display below:</u>

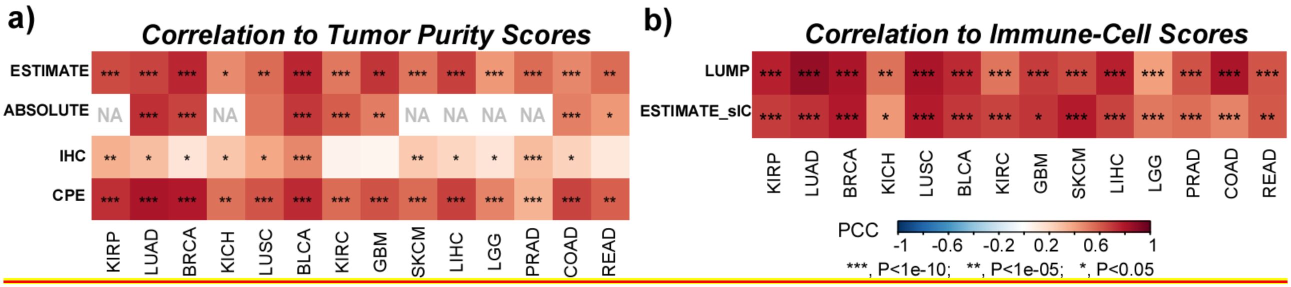

In response to the reviewer's point, <u>we will also add (i) a sentence in Discussion to alert the reader that our DNAm reference matrices yield predictions that should be interpreted with caution, and (ii) a Supplementary Table ranking the tissue-specific scRNA-Seq and DNAm reference matrices by their validation accuracies in the independent scRNA-Seq and DNAm datasets.</u> We believe that this ranking of tissues will be very useful to highlight those which have been more extensively validated.

- The gold standard validation is against FACS-sorted cell types profiled using the DNA methylation Infinium array. The authors provide four such validations - hepatocytes in liver, neuron vs. glia in brain tissue, Dermis vs epidermis in skin, and endocrine vs. epithelial cell types in pancreas. EPISCORE performs relatively well on the first three , which are shown in main figures, but performs poorly on the 4th, which is shown only as a supplemental figure.

Despite the fact that all QC metrics appear to look fine for the pancreatic scRNA-seq clusters and imputation (Fig S6), there is significant confusion between ductal and endocrine cells in multiple FACS-sorted samples from different sources, and confusion between acinar and ductal cells in another (Fig S17). So where is this problem coming from, is it the scRNA-seq data, the imputation method, or neither? Was it because the imputation was based on whole-genome data and the test data was on the array platform? Should this be used to omit the pancreatic model from the Atlas? This is important because the main value of this work is for the resource of tissue specific models provided and the reliability.

Response: The reviewer has raised a valid point. As far as Fig.S17a is concerned, the first 3 panels display a very small number of acinar (n=3), beta (n=3) and ductal (n=4) samples from Moss et al Nat Commun 2018. There is surprisingly little information provided in this publication as to how these samples were generated. We must presume that these are FACS-sorted cell populations, yet purity does not seem to have been assessed. In our experience, given the relatively small numbers of samples and given that FACS-cell populations may not be of high purity, we were not expecting big differences in the estimated cell-type proportions. What the EpiSCORE fractions do demonstrate is that there is broad consistency. As far as the other two panels in Fig.S17a are concerned, these display the estimated fractions in Langerhan islet preparations, which once again don't need to be pure. The only expectation here would be that these islet preparations would exhibit an enriched fraction for endocrine cells, as correctly predicted by EpiSCORE (of note by enrichment we here mean a relatively high fraction of endocrine cells). In our sincere opinion, the data being used for validation in Fig.S17a is the main limitation since the purity of these validation samples is questionable, and therefore this does not reflect a quality issue of the pancreas DNAm reference matrix. In support of this, the **data shown in Fig.S17b, as well as in Fig.4b,c,d,f demonstrate that the pancreas DNAm reference matrix is making the correct predictions: PNETs derive mostly from alpha and beta-cells, PAAD derives from ductal cells, and most importantly, EpiSCORE has correctly identified misdiagnosed PAAD cases, which are in fact PNETs.** For this reason, we would not remove the pancreas DNAm reference matrix from this work, because the overall evidence is in favour of this reference matrix being useful.

Comment: The issues with heterogeneity of underlying scRNA-seq data and different quality levels beg the question of whether this approach would be more reliable using a single large scRNA-seq dataset based on a common technology and analysis methods. Such a dataset became available from GTEx/Broad Institute just before (https://doi.org/10.1101/2021.07.19.452954). I realize this dataset came too late for you to analyze, but I think it's important to comment on how this will improve quality, and how quality of the underlying models can be better quantified/validated.

Response: The reviewer has raised an excellent point. However, we opine differently in that use of a single large scRNA-Seq dataset would be disadvantageous, because such a resource is unlikely to offer high-quality tissue-specific atlases for *all* tissue-types. Indeed, based on our experience analyzing data from such resources like the Mouse Cell Atlas or the Human Cell Landscape (HCL), we observe that the quality of individual tissue-specific datasets varies a lot between tissue-types, which is not entirely surprising since each tissue requires tailored experimental protocols that are also variably suited to different technologies. For instance, **if we take the HCL, which is a single large scRNA-Seq multi-tissue atlas, this would be problematic because (i) for skin no scRNA-Seq dataset was generated, (ii) for liver, the scRNA-Seq atlas failed to capture cholangiocytes, an important component of the liver epithelium, (iii) for pancreas, the scRNA-Seq atlas failed to capture gamma and delta endocrine cells, two of the 4 endocrine cell subtypes, and (iv) for brain, very few neuron cells were captured, only yielding relatively few neuronal marker genes, none of which were imputable for DNAm analysis.** These 4 tissue-types are important ones since for these we have DNAm datasets for objective validation of the derived DNAm reference matrices. Thus, **it is very clear from this, that the use of a single multi-tissue atlas like HCL would not allow us to construct complete and reliable DNAm reference matrices for many tissue-types**.

To make this point clear, we have compared performance of our DNAm-reference matrices for brain and liver to those we would have derived had we used the HCL scRNA-Seq multi-tissue atlas. The results in the validation DNAm datasets displayed below clearly demonstrate that our DNAm reference matrices perform better, demonstrating clearer separability of these FACS-sorted samples in both brain and liver:

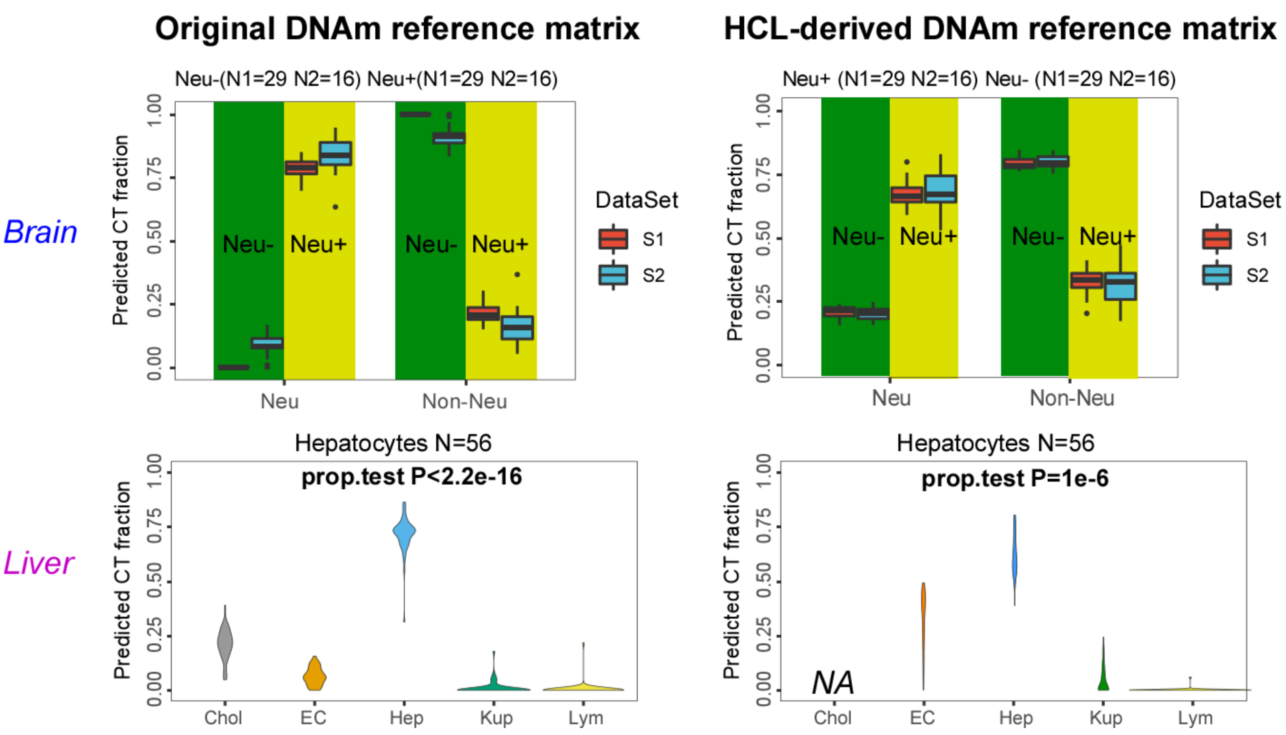

Figure legend: Comparison of our DNAm-atlas to DNAm-reference matrices built from HCL (brain and liver): *Top panel displays the predicted neuronal (Neu) and non-neuronal (Non-Neu) cell-type fractions of Neu+ and Neu- samples in two independent DNAm datasets from Guintivano and Gasparoni et al. The separability between the Neu+ and Neu+ proportions is greater with our DNAm-brain reference matrix (left) compared to that derived from HCL (right). Lower panel makes a similar comparison but now for the liver DNAm-reference matrices in a DNAm dataset encompassing 56 hepatocyte samples. Here too, the separability between the hepatocyte fraction and all other cell-type fractions is greater for our DNAm-liver reference matrix.*

Thus, only if the common technology were optimally designed for each particular tissue-type, e.g. in providing an unbiased catalogue of all major cell-types and at sufficient read depth for each one of them, would such a resource be marginally advantageous. The new single-cell resource from GTEX the reviewer is pointing towards has unfortunately not yet been formally

published, and is only available as a preprint which came out at the same time we submitted this MS for publication.

It should also be clarified that the use of a single large scRNA-Seq atlas would only really be necessary if there was a need for us to cross-compare between tissue-types. However, in our case, we don't need to cross-compare between tissue-types, which is a significant advantage as adjusting for batch effects across tissues is problematic. In the approach taken in this MS, a given scRNA-Seq dataset is only being used to generate a corresponding tissue-specific gene-expression reference matrix, i.e. effectively a task of identifying differentially expressed genes where fold-changes are as big as possible. Indeed, our expression reference matrices are effectively binary entities (expressed/not expressed), from which we then impute the corresponding tissue-specific DNAm reference matrices. Hence, the scRNA-Seq data is being used at a level (binary ON/OFF) which also makes it quite insensitive to the underlying technology. In summary, the main limitation for the resource is certainly **not** the use of one or multiple scRNA-Seq technologies, but the actual imputation method, where we do anticipate that future significant further improvements will be possible.

In response to the reviewer's excellent point, we will include a paragraph in Discussion where we elaborate on the above issues. **In addition, we will also include a new SuppFigure, including the above panel for brain and liver, to convey the message that making use of the HCL resource as a single multi-tissue atlas to generate the expression and DNAm reference matrices, leads to worse validation accuracies in the validation DNAm datasets, as compared to our DNAm-atlas resource where we use different sources for the tissue-specific scRNA-Seq atlases.**

Minor point: Melanoma is shown in Fig 2 and then again in Fig 6. It is confusing and would be better shown in a single figure/section

Response: We appreciate the reviewer's point, but we don't see this as a problem, since Fig.2 is dedicated to demonstrating validations, whilst Fig.6 is dedicated to demonstrating how novel insights can be gained through application of the DNAm-atlas.

Reviewer #3:

General Comment: In their manuscript, Zhu et al. present methodology for reference-based deconvolution of DNA methylation data as well as an atlas of deconvoluted DNA methylomes. The novelty of their approach is that reference DNA methylation profiles are not obtained experimentally, but imputed based on available resources of bulk or single-cell gene expression measurements. This has high significance for the field, as reference DNA methylation profiles of major cell types remain rather scarce, whereas the number of cell atlases that are based on single-cell transcriptomic assays is growing rapidly. Overall the proposed approach for atlas construction is original and is based on solid assumptions about strong association between expression levels of cell type-specific marker genes and DNA methylation levels of their promoters.

Response: We sincerely thank the reviewer for taking time to evaluate our manuscript. We are delighted that the reviewer recognizes our work to be of "high significance for the field", and for the significant originality in the methodological approach.

General Comment: Although the computational method EPISCORE underlying current work was published earlier (Teschendorff et al., Genome Biology, 2020), in the current manuscript the authors apply it to variety of datasets and complex biological systems to clearly demonstrate its benefits and provide a resource of imputed DNA reference matrices for subsequent studies. The authors creatively designed numerous validation experiments to prove the feasibility and biological meaningfulness of obtained results. Overall, the framework appears to generate robust cell type estimates in multiple tissues that potentially lead to novel disease-relevant insights. Provided source code and example analyses could be run and reproduced seamlessly. Nevertheless, in my opinion there are several major gaps in validation analyses summarised in several points below, in particular the absence of clear-cut comparison to an appropriate ground truth.

Response: We sincerely thank the reviewer for recognizing that we have come up with creative and numerous ways to validate our DNAm reference matrices. This was indeed one of the most significant challenges, as for tissues other than blood it is extremely difficult to find data where such validation analyses can be performed. Indeed, we need to stress here that blood is a

unique tissue, for which highly accurate DNAm reference matrices already exist. Purpose of our DNAm-atlas is to provide DNAm reference matrices for solid tissues.

Specific Comment 1): During the validation of the tissue-specific reference gene expression matrices the prediction accuracy in independent scRNA-seq datasets was sometimes rather low for certain tissues and cell types (e.g. skin). Could this ambiguity result in wrong or biased composition estimates and lead to erroneous findings? Can authors suggest an approach to measure and mitigate the impact of these inaccuracies?

Response: The reviewer has raised an excellent point. The overall validation accuracy for the skin mRNA expression reference matrix was 0.75, which admittedly is lower than the typical >0.9 accuracy displayed by most of the other tissues. However, in the case of skin this could reflect issues with the validation scRNA-Seq dataset, and may not necessarily reflect the quality of the expression reference matrix, as indeed for Skin the validation of the DNAm reference matrix shown in Fig.2c,d,e is clearly indicative of the high quality of this DNAm reference matrix. In our sincere opinion, the reviewer's concern would apply to tissues like colon and kidney for which the validation accuracies were just over 0.5. **In response to the reviewer's point, we have now regenerated the expression and DNAm references for colon and kidney using new recently generated and higher-quality scRNA-Seq datasets, or by lowering the cellular resolution of the previously used ones. The new validation accuracies for kidney, which are substantially higher, are shown below:**

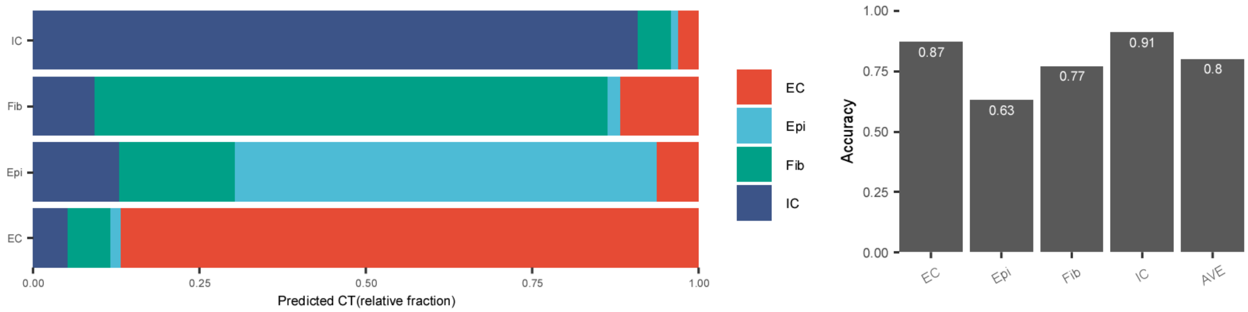

The right panel in the above figure thus demonstrates **that the average classification accuracy for the kidney expression reference matrix is 0.8,** up from 0.52. With this new

kidney DNAm reference matrix, the estimates of tumor purity and total immune-cell fractions in KIRC, KIRP and specially KICH also improve, as shown in a new Fig.2a-b, which for convenience we display below:

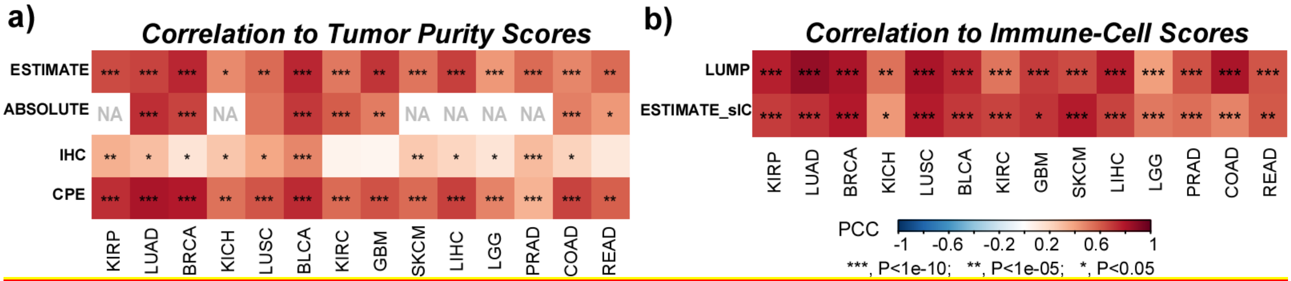

As we can see from the new panels Fig.2a-b above, the kidney DNAm reference matrix performs very well in predicting tumor purity and immune-cell scores across the 3 kidney cancer-types (KIRP, KICH and KIRC).

For colon, we have also regenerated a new expression reference matrix, which now also achieves an overall accuracy of 0.8 in the independent scRNA-Seq dataset. We display the revised SuppFig. for colon tissue below:

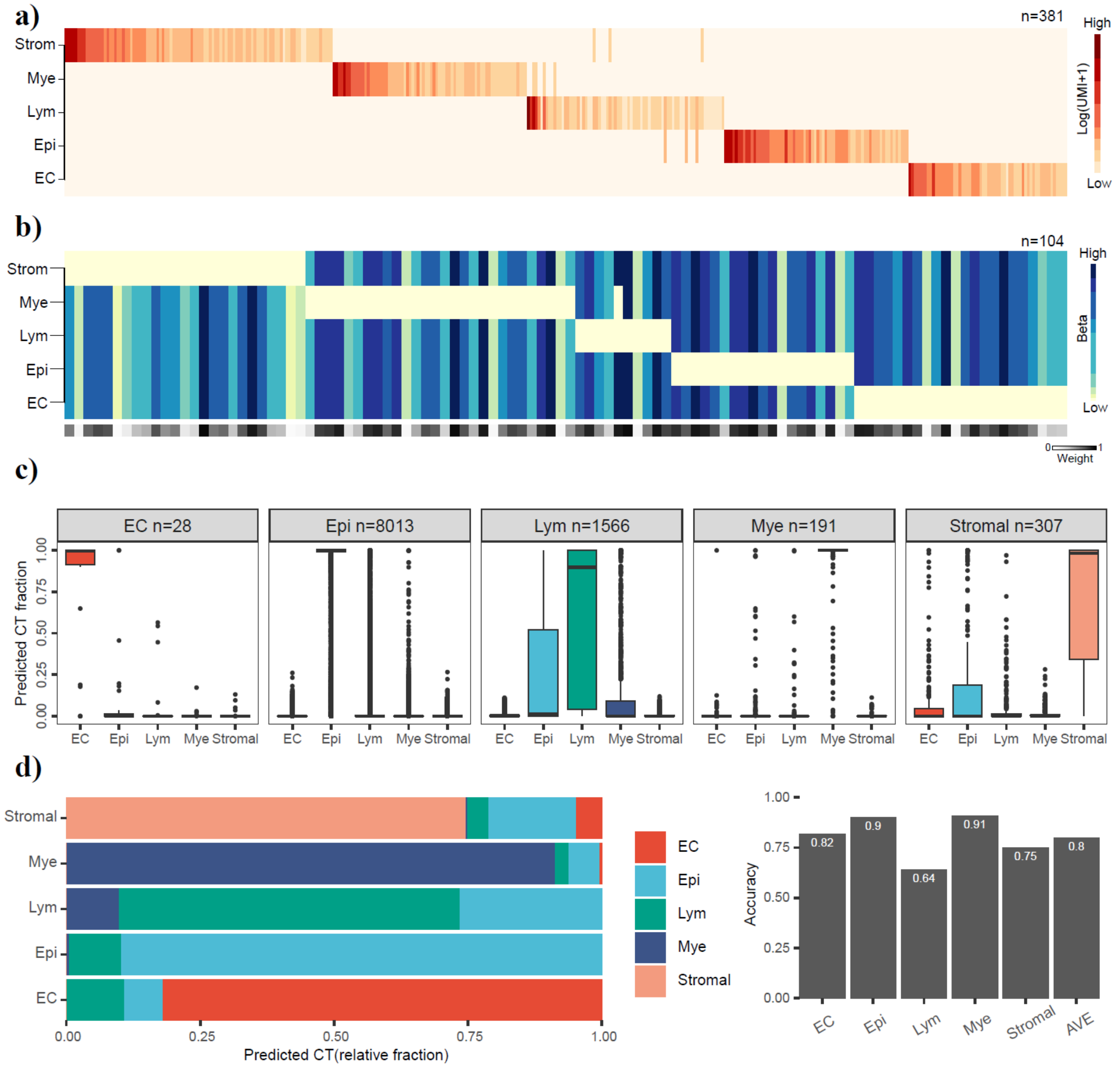

Focusing on panel-d) in the above figure, we can see that the validation accuracy is significantly higher compared to the previous version. In summary, we will revise Fig.1, Fig.2 and the relevant Supp.Figs.

In addition, we will also add a new Supplementary Table, ranking tissues by the validation accuracies attained by the expression and DNAm reference matrices. This will help readers assess which tissue-specific reference matrices have been most extensively validated.

Specific Comment 2): For two independent scRNA-seq datasets used to construct reference gene expression matrices, what were the criteria to select the primary dataset for marker selection and the secondary for validation? Could authors perform a round of reciprocal construction-validation procedures and compare the obtained markers and cell type estimation accuracy to their original results?

Response: The reviewer has raised a good point. The criteria for selecting the primary set was (i) the number of distinct cell-types profiled, as well as (ii) the number of cells within each cell-type. In effect, for the reference matrix construction it was important that the primary set contained as many of the relevant cell-types as possible and also in sufficient numbers to ensure adequate power to detect a sufficient number of marker genes for each cell-type. For most tissues it was a clear choice as to which scRNA-Seq dataset should be primary, and which one should be used for validation. However, for a few tissues (e.g. liver) the choice was more ambiguous. In response to the reviewer's point, we have now performed the reciprocal analysis for liver, results of which will be displayed in a new Suppl.Fig. which for convenience we display below:

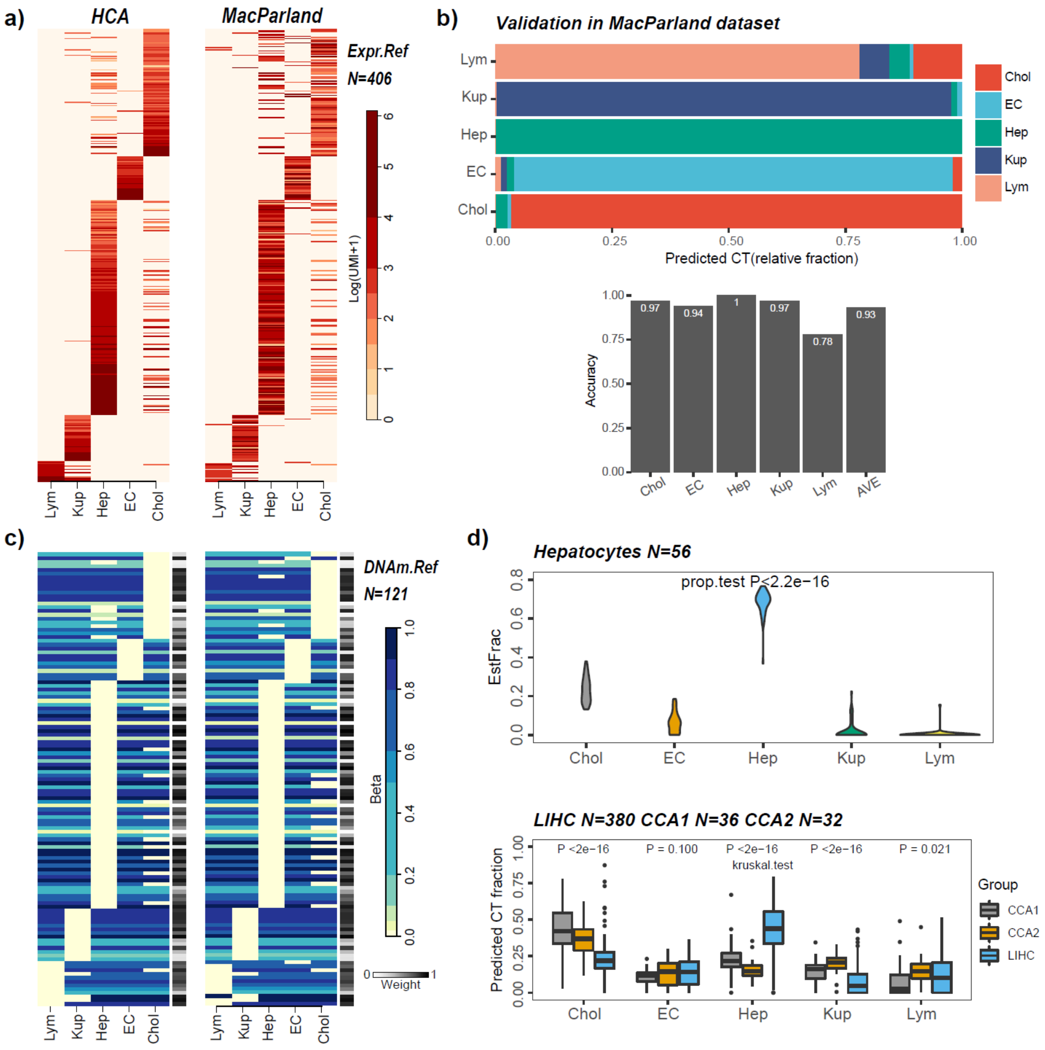

***Figure legend: a)** The two expression reference matrices for liver tissue derived from two scRNA-Seq liver atlases from the Human Cell Landscape (HCL) and MacParland et al. Shown are the expression profuiles for the same 5 cell-types and a common set of 406 overlapping marker genes. **b)** Predicted cell-type fractions and overall classification accuracy as obtained by applying the expression reference from HCL to the scRNA-Seq dataset from MacParland. **c)** The two imputed DNAm reference matrices derived from the corresponding expression reference matrices in a), as defined over a common set of 121 marker genes. **d)** Validation of the HCA liver DNAm reference matrix in independent DNAm datasets profiling hepatocytes (top panel) and TCGA hepatocellular carcinoma (LIHC) and cholangiocarcinoma (CCA1/2). To be compared with Fig.2 where results are presented for the liver DNAm reference matrix derived from MacParland.*

This new figure clearly demonstrates that results are very robust. Indeed, it is worth noting the very strong overlap and correlation of the expression and DNAm reference matrices obtained from the original and reciprocal analyses.

Comment 3). While the validation of imputed DNA methylation reference matrices appears convincing, it is rather indirect. Would it be possible to validate the imputed matrices directly on SCM2 and RMAP datasets (or any other similar dataset with both data modalities, e.g. brain tissue data presented in the manuscript) by comparing them to respective ground truth matrices using correlation or other appropriate distance?

Response: We thank the reviewer for raising this excellent point. Broadly speaking, this validation strategy is indeed what we pursued with the brain DNAm reference matrix, by comparing it to the snmC-Seq2 data in Fig.3b-c. While we do not explicitly compare the matrices to each other, we displayed the data in the form of boxplots (Fig.3b-c) to show that a marker gene highly expressed in one specific brain cell subtype (for which the imputed promoter DNAm value is 0), displays low promoter snmC-Seq2 DNAm levels in that same cell-type compared to all other cell-types (Fig.3b). For each marker gene, we thus obtain a t-statistic and P-value. We then plot the distribution of t-statistics over all marker genes of a given cell-type (Fig.3c). This shows that there is a clear trend for the marker genes to display lower promoter DNAm levels in the cell-types they are overexpressed in, compared to all other cell-types. Thus, Fig.3b-c provides a direct validation of the imputed DNAm reference matrix for brain, albeit on a relative scale.

To perform a direct validation of the imputed DNAm reference matrix on an absolute scale is more problematic because of the extremely high sparsity of the snmC-Seq2 data: indeed, the reviewer is perhaps unaware of the fact that approximately 90% of the entries in the snmC-Seq2 are missing due to low read coverage. This means that deriving a pseudo-bulk DNAm profile for each cell-type (i.e. averaging over the non-missing values in each cell-type), this pseudo-bulk derived DNAm value is obtained on average from about only 60 cells per cell-type. Nevertheless, in response to the reviewer's point, we have computed the Median Absolute Deviation (MAD-score) and Pearson Correlation Coefficient (PCC) between the imputed and the snmC-Seq2 derived pseudo-bulk DNAm matrix, observing a MAD-value of 0.11 ($P<0.0001$ as

assessed by Monte-Carlo Randomization with 10,000 permutations) and a PCC-value of 0.56 (Correlation test P<2e-16). We display these data in the figure below, which will be added as a new Supp.Fig. in a revised version of the MS:

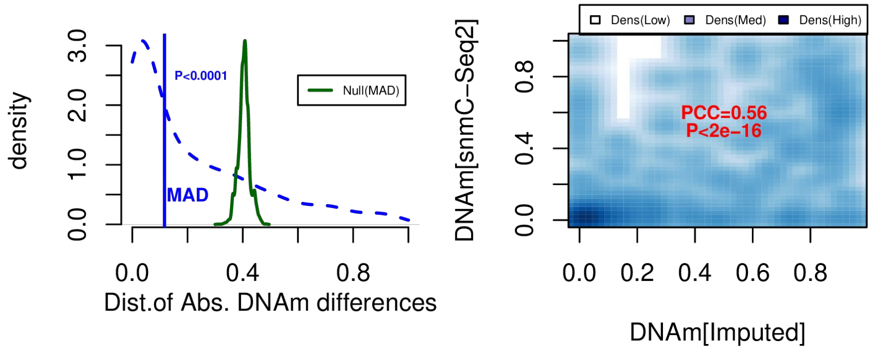

Figure legend: Left panel displays the distribution of absolute differences between the imputed and the pseudo-bulk snmC-Seq2 DNAm data matrices (dashed-curve), with the median absolute deviation (MAD) indicated by a vertical solid blue line. Green curve denotes the MAD values from 10,000 Monte-Carlo randomizations. P-value is obtained by comparing the observed MAD value to the null distribution. Right panel is a smoothed scatterplot of the imputed DNAm values and the pseudo-bulk snmC-Seq2 ones. PCC denotes the Pearson Correlation Coefficient and Correlation test P-value is given.

As far as the SCM2 and RMAP datasets are concerned, these can't be used for two reasons: First of all, these datasets are used as part of the imputation, so they don't satisfy the independence criterion required by a validation set. Second, most of the samples from SCM2 and RMAP do not in fact represent purified samples. This of course is not a limitation for finding imputable genes where promoter DNAm and gene-expression are highly anti-correlated, but it does present a limitation for the type of validation the reviewer was suggesting.

Comment 4) Despite that numerous validations of the proposed methodology have been performed, a direct and quantitative comparison to a plausible ground truth is missing. Could the authors use one of the tissues for which both, reference (sc)RNA-seq and DNA methylation data of the same cell types available, and compare the EPISCORE results to those of any of the standard reference-based deconvolution algorithms (e.g Houseman's method, EpiDISH etc)? The brain tissue data presented in the manuscript appear to be perfectly suitable for this

validation. How do proportions of brain cell types in schizophrenia recovered with EPISCORE-imputed DNA methylation matrix compare to those obtained using e.g. the pseudo-bulk scnmC-seq-based reference and how consistent would downstream annotation and interpretation be? Another possibility would be to use data obtained with one of the multi-modal single-cell protocols yielding expression and methylation data from the same cell (e.g. mouse gastrulation atlas in Argelaguet, Nature, 2019).

Response: We thank the reviewer for the excellent suggestions. Although the brain snmC-Seq2 data is very sparse (fraction of NAs is 90%) we agree that we could try to build a pseudo-bulk DNAm reference matrix from it, which we could then apply to independent DNAm datasets from purified brain cell-types, as well as to the SZ EWAS study, in order to compare results obtained with our EpiSCORE-derived DNAm reference. Below, in the scatterplots to the left, we display and compare the neuronal fraction estimated from the snmC-Seq2 derived pseudo-bulk reference matrix to the one from our brain DNAm-reference matrix (EpiSCORE) in the FACS-sorted Neu+ and Neu- samples from Guintivano et al and the Neu+, Bulk PFC and Neu-samples from Gasparoni et al:

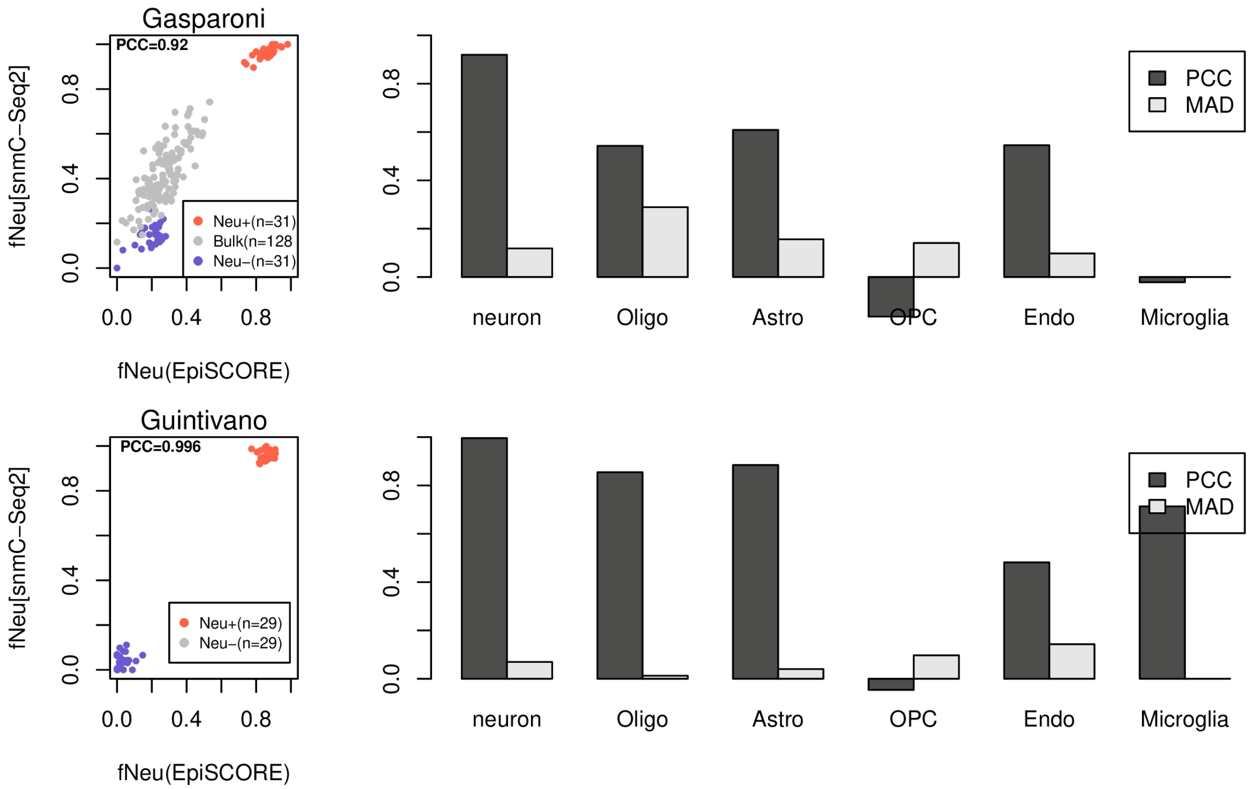

As we can see, there is excellent agreement. To the right we display the Pearson Correlation Coefficient (PCC) and Median Absolute Deviation (MAD) between the snmC-Seq2 and EpiSCORE derived cell-type fractions, and for each brain cell-type. The most important measure to consider here is MAD, since for a cell-type that is only present in very low proportions, the corresponding PCC value is not expected to be large or may even be negative, even if the MAD between the two methods is very small. As we can see from both Guintivano and Gasparoni, the MAD is generally less than 0.2 and often also less than 0.1, suggesting good agreement of our EpiSCORE derived estimates with those from the snmC-Seq2 derived brain reference.

In response to the reviewer's point, we will include the above figure in the revised version of the MS. In the revised version of the MS, we will also compare the results from both brain DNAm reference matrices on the SZ-EWAS study.

As far as the mouse gastrulation multi-modal data is concerned this is not only problematic because of the high sparsity of the underlying single-cell DNAm data, but also because it is

mouse data, and our DNAm-atlas is aimed at human tissues. We should clarify that the integrated databases SCM2 and Epigenomics Roadmap that we use to identify imputable genes are based on human samples, so all the validations of our DNAm reference matrices need to be in human tissue.
In future, it would be important to develop an analogous DNAm-atlas for mouse, specially given that Illumina now has a mouse methylation array, and there will be a great need to perform cell-type deconvolution in mouse tissues, so this is an exciting and necessary project for the future of the epigenomics field.

Comment 5) Although not explicitly emphasized, there is no obvious reason why similar atlases cannot be constructed based on bulk gene expression data, either array- or RNA-seq-based. In case it is indeed possible, rich reference epigenome resources (e.g. BLUEPRINT project for hematopoietic cells) can be used to perform a direct validation as described above.
Response: We appreciate the reviewer's point, but of course there is limited availability of bulk mRNA expression profiles of purified (e.g. FACS-sorted) samples for all cell-types within a given tissue, with the exception of a tissue like blood for which BLUEPRINT has generated mRNA and DNAm profiles for all major purified blood cell-subtypes. Thus, the reviewer's strategy can't be implemented for the solid tissue-types that we are aiming for. Incidentally, in the context of blood tissue, we did already attempt a strategy similar to what the reviewer is implying and this was shown in our EpiSCORE 2020 paper, where as proof of principle we imputed a DNAm reference matrix for peripheral blood mononuclear cells from a corresponding PBMC scRNA-Seq atlas, which we then validated using independent DNAm data from purified cell-types (FACS-sorted cell populations). For convenience, we add the corresponding figure from that paper below:

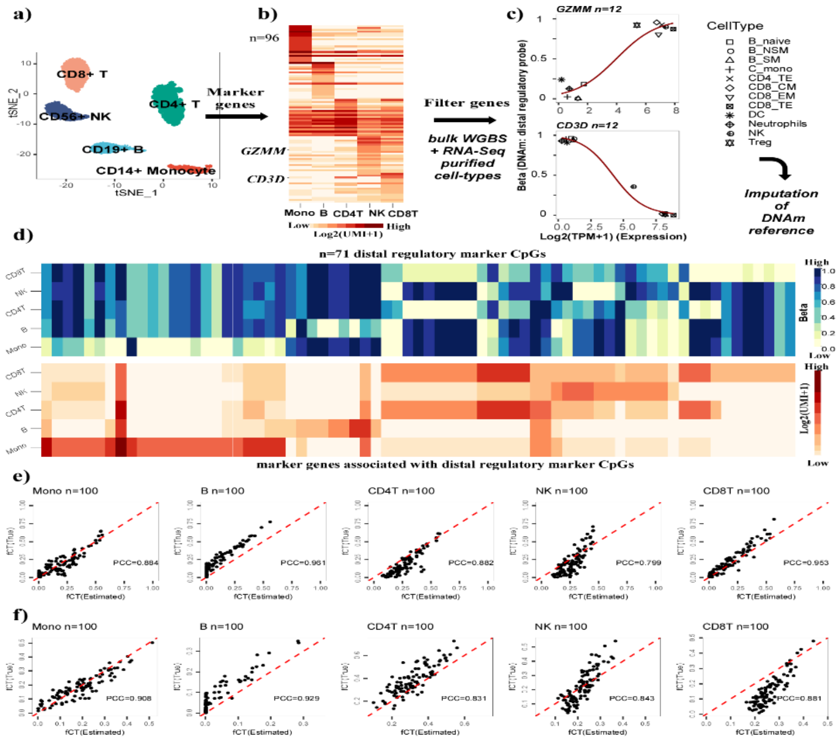

Figure-6 from EpiSCORE 2020 paper: ***Proof of EPISCORE concept in peripheral blood. a)*** *Dimensional reduction and t-SNE embedding of a 10X dataset profiling 68,000 peripheral blood mononuclear cells (PBMCs), depicting the resulting cell clusters, annotated to PBMC cell-types using a bulk RNA-Seq reference matrix for PBMCs.* ***b)*** *The expression reference matrix over 5 main PBMC cell subtypes and 96 marker genes, as constructed from the scRNA-Seq data in a).* ***c)*** *Two examples of marker genes, where differential expression across blood cell subtypes (as assessed using independent bulk RNA-Seq and DNAm data of purified blood cell types), is associated with corresponding differential DNAm at a CpG mapping to a distal regulatory element of the gene. The y-axis labels DNAm at this CpG, x-axis labels the normalized gene expression value, and datapoints are shown for all matching blood cell subtypes. A fit from a logistic regression is shown, which is used to impute DNAm at this CpG from the expression values in the scRNA-Seq reference matrix, after suitably normalizing the expression data.* ***d)*** *Heatmaps displaying the imputed DNAm reference matrix for 71 marker CpGs (top panel) and the corresponding scRNA-Seq reference matrix for X marker genes associated with these 71 CpGs.* ***e)*** *Validation of the imputed DNAm reference matrix using in-silico mixtures of PBMC cell types, using Illumina 450k profiles from Reinius et al. For each of the 5 PBMC cell-types we plot the estimated fractions (x-axis) vs. the true fractions (y-axis), for each of 100 in-silico mixtures. Mixture weights were simulated from a uniform Dirichlet distribution. PCC-values are given.* ***f)***

As e), but with mixture weights drawn from a non-uniform Dirichlet distribution, with mean and variance for each cell-type matched to observed data.

Let us clarify here again that the current manuscript is about providing a resource to the community aimed at cell-type deconvolution in solid tissue-types. As such, to perform additional analyses on BLUEPRINT data would not really help address the aim of our DNAm-atlas, which is to enable cell-type deconvolution of solid tissue-types at an unprecedented higher cellular resolution.

Comment 6) What are the reasons for applying the proposed framework separate to each tissue? Would it be possible to impute DNA methylation matrices across multiple tissues and use them to estimate cell type proportions in samples of unknown origin? Could the authors test this?

Response: The reviewer raises a very interesting question. The answer as to which strategy is more appropriate largely depends on the downstream application. For the applications we are interested in (e.g. identifying misdiagnosed cancer-cases within a tumor-type, novel prognostic subgroups within a tumor type), it makes more sense to take a tissue-centric approach, as done in our MS. There are a number of reasons for this: First of all, the strategy the reviewer is proposing would make more sense if we can assume that non tissue specific cell-types like endothelial or fibroblast cells have highly similar profiles irrespective of the tissue they are found in. While there is evidence that if we were to do a clustering of say all cell-types within two distinct organs (say lung and brain) that the endothelial cells from lung and brain would cluster together, this only means that they are more similar to each other compared to the other cell-types within these tissues. It does not mean that their profiles are sufficiently similar. Indeed, there will be critical differences between the endothelial cells found in different tissue-types like lung or brain. Thus, it seems safer to us, to build tissue-specific reference matrices that treat each cell-type in the tissue as a unique cell-type, in other words to make a distinction between say the endothelial cells found in lung from those found in brain. A second reason is that the reviewer's proposed strategy would also require comparisons of scRNA-Seq datasets across many different tissue-types, generated with potentially different technologies or sequencing

depths, which makes quantitative comparisons of expression problematic. While binarizing gene expression into ON/OFF states can partly circumvent this problem (and indeed we have explored this), it is challenging to retrieve enough marker genes from this approach as there are correspondingly many more cell-types. Alternatively, one could also try to cross-compare tissue-specific scRNA-Seq datasets if these have all been generated within the same large atlas (e.g. the Human Cell Landscape – HCL). However, this strategy leads to worse performance in the applications considered in this MS. For instance, for skin, HCL did not generate any atlas, for the liver HCL-atlas this failed to capture cholangiocytes (an important component of the liver epithelium) not allowing us to apply it to liver cancer diagnosis, for the pancreas HCL atlas, gamma and delta endocrine cells are also absent, which is not ideal for diagnostic applications in pancreatic cancer, and the brain-HCL atlas failed to capture sufficient numbers of neurons not allowing identification of sufficient neuronal marker genes that are imputable, rendering the resulting DNAm-reference for brain not applicable. This clearly shows that it is much better to use high-quality scRNA-Seq atlases tailored for each tissue-type. **In a revised version of a MS, we will include a paragraph in Discussion to clarify the above points, and we will also include a new SuppFig. to demonstrate that our DNAm-atlas validates better in the independent DNAm datasets compared to a DNAm-atlas derived from a single multi-tissue scRNA-Seq resource such as HCL.** Below we provide a preliminary version of this figure for the case of brain and liver tissue, which demonstrates the better validation performance of our DNAm reference matrices:

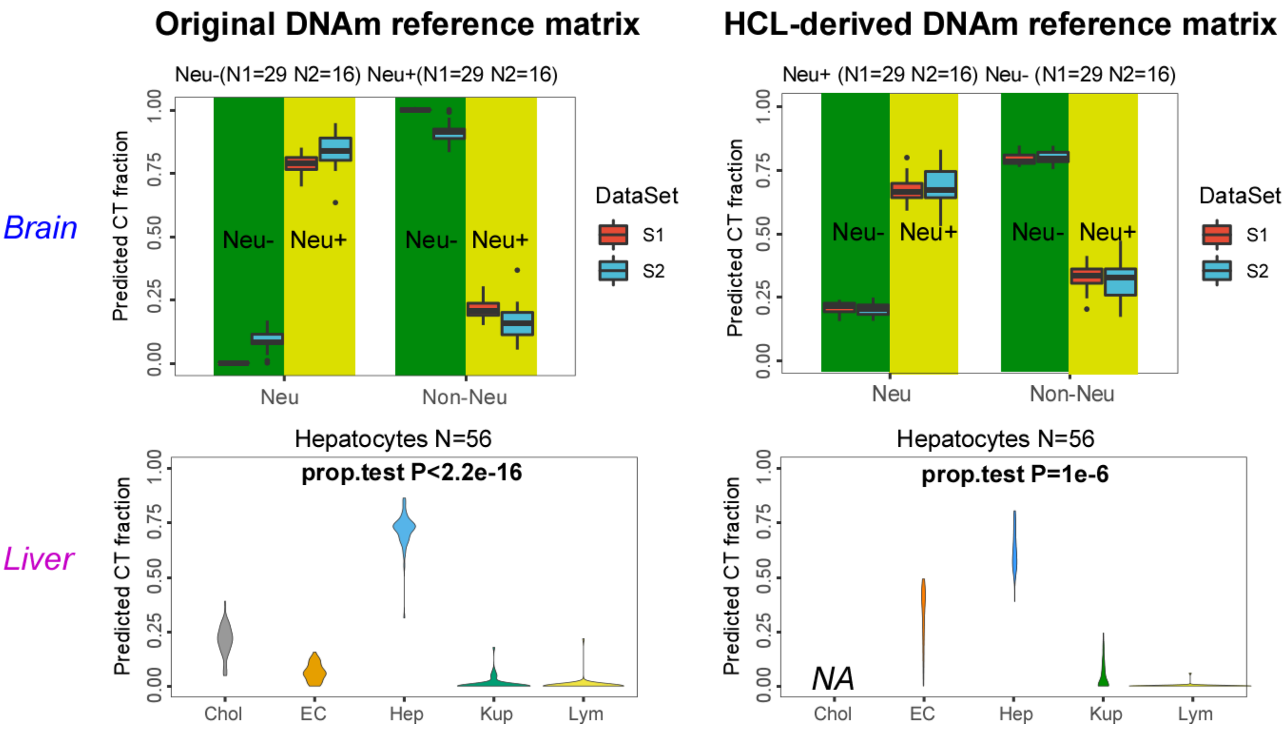

Figure legend: Comparison of our DNAm-atlas to DNAm-reference matrices built from HCL (brain and liver): *Top panel displays the predicted neuronal (Neu) and non-neuronal (Non-Neu) cell-type fractions of Neu+ and Neu- samples in two independent DNAm datasets from Guintivano and Gasparoni et al. The separability between the Neu+ and Neu+ proportions is greater with our DNAm-brain reference matrix (left) compared to that derived from HCL (right). Lower panel makes a similar comparison but now for the liver DNAm-reference matrices in a DNAm dataset encompassing 56 hepatocyte samples. Here too, the separability between the hepatocyte fraction and all other cell-type fractions is greater for our DNAm-liver reference matrix.*

Finally, while we agree that it would be valuable to be able to classify a sample of unknown origin, we see this is as a completely separate question and project, which would require an entirely different approach. For instance, such a question is more interesting in the context of measuring DNAm in cell-free DNA (serum).

Minor points:

Comment: On the minor side, which is however quite important for the statistical soundness of the obtained conclusions, I wonder whether a proportion test perhaps be more suitable for testing the differences of recovered cell type fractions as compared to the Wilcoxon rank sum test used for many analyses throughout the manuscript?

Response: The reviewer has raised a valid statistical question. If we are comparing a cell-type fraction between datasets (e.g. comparing a hepatocyte fraction between TCGA hepatocellular carcinoma and TCGA cholangiocarcinoma as done in right panel of Fig.2f), using a Wilcoxon rank sum test is entirely appropriate. The reviewer is therefore probably referring to the scenario where we are comparing different cell-type fractions within the same dataset (e.g. comparing hepatocyte to cholangiocyte fractions within TCGA hepatocellular carcinomas), where there is statistical dependence or relation between any two cell-type fractions within each sample of the dataset. In this case, we agree that an unpaired Wilcoxon rank sum test would not be strictly speaking correct, although owing to the fact that we have many cell-types (not just 2) this is not going to greatly affect the P-values. For instance, we have checked that the P-value bound shown in the left panel of Fig.2f is correct, since we obtain the same value using a test for proportions (prop.test function in R) or a paired Wilcoxon rank sum test. In the revised version we will check every single P-value to ensure that our results are statistically sound, as well as correcting the figure legend. In the case of Fig.2f, the legend was inaccurate, as different tests were implemented for the two panels in Fig.2f.

Comment: Last but not leasrt, the manuscript is written very clearly, with high accuracy in details, comprehensive and well-readable graphics. Relevant work is referenced appropriately.

Response: We greatly appreciate this positive comment.

Decision Letter, first revision:

Subject: Decision on appeal of Nature Methods submission NMETH- RS46537B-Z
Message:

14th Oct 2021

naturereseach

Dear Dr Teschendorff,

Thank you for your letter asking us to reconsider our decision on your Resource, "A pan-tissue DNA methylation atlas enables in-silico microdissection of human tissue methylomes at cell-type resolution". After careful consideration we have decided that we are willing to consider a revised version of your manuscript that includes the additional data presented in your point-by-point rebuttal letter.

When revising your paper:

* include a point-by-point response to our referees and to any editorial suggestions

* please underline/highlight any additions to the text or areas with other significant changes to facilitate review of the revised manuscript

* address the points listed described below to conform to our open science requirements

* ensure it complies with our general format requirements as set out in our guide to authors at www.nature.com/naturemethods

* resubmit all the necessary files electronically by using the link below to access your home page

[REDACTED]

Note: This URL links to your confidential home page and associated information about manuscripts you may have submitted, or that you are reviewing for us. If you wish to forward this email to co-authors, please delete the link to your homepage.

We hope to receive your revised paper within 4 weeks. If you cannot send it within this time, please let us know. In this event, we will still be happy to reconsider your paper at a later date so long as nothing similar has been accepted for publication at Nature Methods or published elsewhere.

OPEN SCIENCE REQUIREMENTS

REPORTING SUMMARY AND EDITORIAL POLICY CHECKLISTS
When revising your manuscript, please submit reporting summary and editorial policy checklists.

Reporting summary: https://www.nature.com/documents/nr-reporting-summary.zip

Editorial policy checklist: https://www.nature.com/documents/nr-editorial-policy-checklist.zip

If your paper includes custom software, we also ask you to complete a supplemental reporting summary.

Software supplement: https://www.nature.com/documents/nr-software-policy.pdf

Please submit these with your revised manuscript. They will be available to reviewers to aid in their evaluation if the paper is re-reviewed. If you have any questions about the checklist, please see http://www.nature.com/authors/policies/availability.html or contact me.

Please note that these forms are dynamic 'smart pdfs' and must therefore be downloaded and completed in Adobe Reader. We will then flatten them for ease of use by the reviewers. If you would like to reference the guidance text as you complete the template, please access these flattened versions at http://www.nature.com/authors/policies/availability.html.

IMAGE INTEGRITY
When submitting the revised version of your manuscript, please pay close attention to our href="https://www.nature.com/nature-research/editorial-policies/image-integrity">Digital Image Integrity Guidelines. and to the following points below:

-- that unprocessed scans are clearly labelled and match the gels and western blots presented in figures.
-- that control panels for gels and western blots are appropriately described as loading on sample processing controls
-- all images in the paper are checked for duplication of panels and for splicing of gel lanes.

Finally, please ensure that you retain unprocessed data and metadata files after publication, ideally archiving data in perpetuity, as these may be requested during the peer review and production process or after publication if any issues arise.

DATA AVAILABILITY
Please include a "Data availability" subsection in the Online Methods. This section should inform readers about the availability of the data used to support the conclusions of your study, including accession codes to public repositories, references to source data that may be published alongside the paper, unique identifiers such as URLs to data repository entries, or data set DOIs, and any other statement about data availability. At a minimum, you should include the following statement: "The data that support the findings of this study are available from the corresponding author upon request", describing which data is available upon request and mentioning any restrictions on availability. If DOIs are

provided, please include these in the Reference list (authors, title, publisher (repository name), identifier, year). For more guidance on how to write this section please see: http://www.nature.com/authors/policies/data/data-availability-statements-data-citations.pdf

CODE AVAILABILITY
Please include a “Code Availability” subsection in the Online Methods which details how your custom code is made available. Only in rare cases (where code is not central to the main conclusions of the paper) is the statement “available upon request” allowed (and reasons should be specified).

We request that you deposit code in a DOI-minting repository such as Zenodo, Gigantum or Code Ocean and cite the DOI in the Reference list. We also request that you use code versioning and provide a license.

For more information on our code sharing policy and requirements, please see: https://www.nature.com/nature-research/editorial-policies/reporting-standards#availability-of-computer-code

SUPPLEMENTARY PROTOCOL
To help facilitate reproducibility and uptake of your method, we ask you to prepare a step-by-step Supplementary Protocol for the method described in this paper. We encourage authors to share their step-by-step experimental protocols on a protocol sharing platform of their choice and report the protocol DOI in the reference list. Nature Research's Protocol Exchange is a free-to-use and open resource for protocols; protocols deposited in Protocol Exchange are citable and can be linked from the published article. More details can found at www.nature.com/protocolexchange/about.

ORCID
Nature Methods is committed to improving transparency in authorship. As part of our efforts in this direction, we are now requesting that all authors identified as ‘corresponding author’ on published papers create and link their Open Researcher and Contributor Identifier (ORCID) with their account on the Manuscript Tracking System (MTS), prior to acceptance. This applies to primary research papers only. ORCID helps the scientific community achieve unambiguous attribution of all scholarly contributions. You can create and link your ORCID from the home page of the MTS by clicking on ‘Modify my Springer Nature account’. For more information please visit please visit www.springernature.com/orcid.

We look forward to hearing from you soon.

Sincerely,
Lei

Lei Tang, Ph.D.
Senior Editor
Nature Methods

Author Rebuttal, first revision:

Detailed Response to Reviewers' Comments:

Reviewer #1:

General Comment: One of the fundamental challenges facing EWAS with bulk-tissue methylation data lies in the interpretation of differentially methylated CpGs derived from such data as bulk-tissue is comprised of a myriad of different cell types, each with their own unique methylation signature. Thus, to obtain a more complete understanding of the biological processes underlying some disease, one needs to look at how DNA methylation is altered within specific cell populations. This fact is now widely recognized within the epigenomics research community. The atlas described here has the potential to facilitate such analyses when used in tandem with recently published methodologies that allow for cell-specific analyses of bulk-tissue methylation data (e.g., cellDMC, TCA, etc.) and therefore, fills a critical niche among methods/tools for analysis array-based DNA methylation collected in the context of large-scale epidemiological studies. The described atlas was validated using a number of publicly available data sets from different solid tissue types (e.g., liver, brain, pancreas, skin, etc.), which all showcased some of the novel insights that can be gleaned from such a resource. This reviewer is very enthusiastic about the atlas described in this manuscript and the potential avenues it could open up within the epigenomics research community. That said, there are certain points

that I would like the authors to address as I feel they could strengthen the manuscript and the corresponding DNA methylation atlas.

Response: We would like to thank the reviewer for taking time to read and evaluate our MS. We are delighted that the reviewer shares our enthusiasm for this work, and that he/she agrees that this DNAm-atlas will be of great value to the eigenomics community.

Specific comments:

Comment 1. The one major point that deserves some discussion/consideration is that all of the DNA methylation reference matrices are based on the now discontinued Illumina HumanMethylation450 array (aka 450K array). This by no means invalidates or diminishes the contributions of this work as there a great many publicly available 450K methylation data sets consisting of DNA methylation profiled in the solid tissue types that comprise the provided atlas, however the community would be best served if reference matrices based on the EPIC array were also provided. Recognizing the extent of work that such would entail, it would be sufficient to demonstrate that the 450K reference matrices comprising the existing atlas are sufficient to enable accurate and reproducible deconvolution estimates when applied to a EPIC data set. Since ~90% of the CpGs on the 450K array are also contained on the EPIC array, chances are that the majority of CpGs in the existing reference sets are also on the EPIC array and thus, one could simply use the overlapping CpGs to do the deconvolution. The concern that I have is that some of the reference sets are rather small in terms of the number of CpGs relative to the number of cell types being deconvoluted and my fear is that removing even a small number of CpGs from the reference matrix might throw off the deconvolution estimates. Another consideration regarding the EPIC array is that since it has twice the coverage of the 450K array, might it be possible to construct reference matrices based on the EPIC array that improve the accuracy of deconvolution estimates beyond that which can be obtained considering only 450K probes?

Response: We thank the reviewer for raising this point. First of all, we should apologize for a typo in the legend to Fig.2f where we had incorrectly stated that the hepatocyte data is 450k, when it is in fact EPIC. It was correctly stated in Methods. Thus, we had in fact already included one EPIC dataset, namely the validation of the liver DNAm reference matrix in the hepatocyte

EPIC data as shown in Fig.2f (displayed again below for your convenience), demonstrating a strong validation.

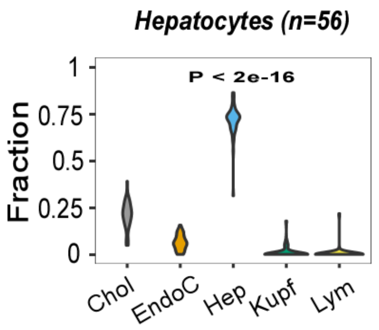

We have now corrected the legend to Fig.2f.

However, let us also clarify that there is no reason why validation accuracy in EPIC data should be worse than in 450k. To understand this, let us point out that (i) our DNAm reference matrices are not just derived from 450k data but also from WGBS data of the Epigenomics Roadmap, and (ii) that they are not directly generated from 450k or WGBS data. Our DNAm reference matrices are generated by imputation from a corresponding tissue-specific scRNA-Seq reference matrix. The imputation step filters marker genes in the mRNA expression reference matrix for a subset of marker genes for which there is an anticorrelation between promoter DNAm and gene-expression, as assessed over two separate databases of matched DNAm and mRNA expression profiles: only one of these databases is 450k based (SCM2) whereas the one derived from the Epigenomics Roadmap is WGBS-based. When building the final DNAm reference matrix we did not take the intersection of the two filtered marker gene lists, but the *union*, which means that our DNAm reference matrices are *not* actually restricted to 450k data. Indeed, the reviewer should also bear in mind that our DNAm reference matrices are defined at the level of gene-promoters, so as long as there is one probe or one CpG with sufficient coverage in the gene-promoter, a DNAm-value can be assigned to that marker gene and the gene will not drop out from the analysis. In fact, although 90% of the 450k probes are present on the EPIC array, the relevant question would be, for how many of our anticorrelated (i.e imputable) genes (a total of 2810) there is an EPIC probe mapping to its promoter (within 200bp of the TSS). We have computed this and the fraction is 69.1%. As a comparison, for the 450k array this fraction is 68.7%, so as the reviewer can see, the fractions

are extremely similar and for the EPIC array it is even higher. Thus, applying our DNAm reference matrices to EPIC data would not be a limitation at all. The only limitation stems from the identification of imputable genes, since if we had a comparable database to SCM2 but with EPIC instead of 450k, we would then have been able to have a marginally higher pool of imputable genes. It would not be significantly higher though, since as mentioned earlier, our imputable gene list derives from the union of two separate analysis, one of which was done at the level of WGBS.

Besides the excellent validation in the EPIC hepatocyte dataset (Fig.2f), we have now performed two additional validations in EPIC DNAm datasets: in one case we further validate the skin DNAm reference matrix in an EPIC dataset profiling 8 skin fibroblast samples (Sarkar TJ et al Nat Commun.2020), whilst the other validation is of the brain DNAm reference in an EPIC dataset profiling 100 FACS-sorted neuronal (Neu+) samples (Pai S et al Nat Commun. 2019). For convenience, we display the figure below, which has been added as new SuppFig.S14:

a)

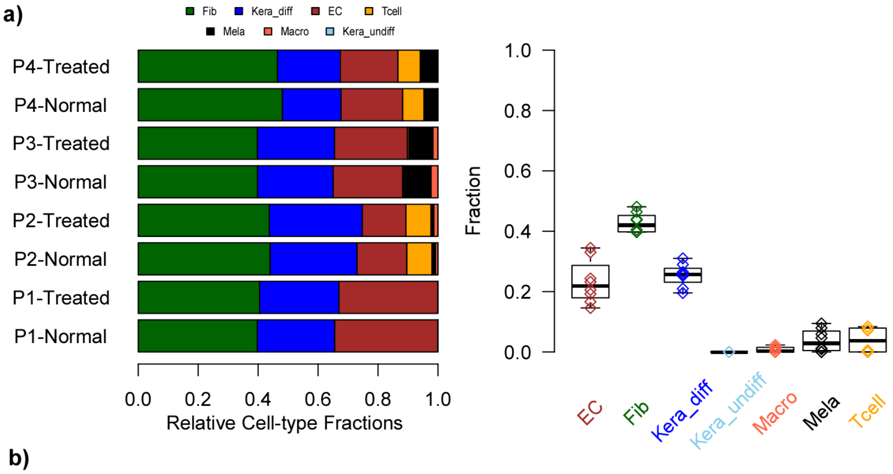

b)

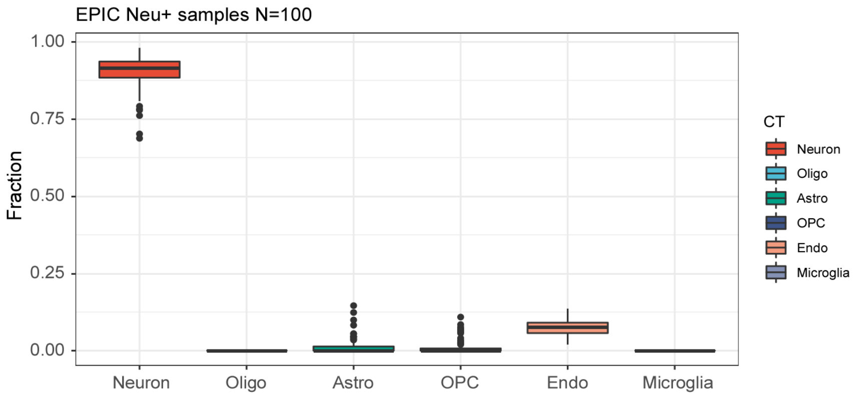

Supplementary Figure 14: Validation of skin and brain DNAm reference matrices in EPIC DNAm datasets. a) *Left barplots display the estimated cell-type fractions using our skin DNAm reference matrix in each of 8 skin fibroblast samples (EPIC data from Sarkar et al). Boxplots to the right help the visual comparison between cell-types, confirming that all 8 samples would be classified as fibroblasts.* ***b)*** *Boxplots display the estimated cell-type fractions using our brain DNAm reference matrix in 100 neuronal (Neu+) samples (EPIC data from Pai et al).*

As we can see from the above figure, all 8 skin fibroblast and all 100 Neu+ samples are classified as fibroblasts and neurons, respectively. In the case of the skin fibroblasts purity is not high, but this is not entirely unexpected given that these samples were cultured from 2mm-punch skin biopsies in a procedure which does not remove the contaminating endothelial cells (EC) (see Vangipuram M et al J Exp Vis 2013).

Comment 2. This might be beyond the scope of the current paper as this comment is less about the atlas itself and more about how this resource fits into the broader context of other recent methodological developments for bulk-tissue methylation data (e.g., cellDMC, TCA, etc.), but I think it would be extremely valuable to cross-compare cell-specific methylation events/effects identified using this atlas in tandem with cellDMC/TCA, when applied to bulk-tissue methylation data in a solid tissue type, to the results of single cell methylation data in the same tissue type. For example, suppose we have 450K methylation data in liver collected from healthy and diseased subjects. If there exists single cell methylation data in the same tissue type for the same biological conditions (doesn't necessarily need to be the exact same subjects with 450K data), are the results for cell-specific methylation effects consistent? Has this been done before?

Response: We agree with the reviewer that in theory doing this would be a great way to validate the DNAm reference matrices, but the problem with this suggestion (as indeed the reviewer was also hinting at) is that such data is not available for any of the solid tissue types, or not available in sufficient numbers to reach solid conclusions. This is certainly true for single-cell DNAm data, as the little single-cell DNAm data that is available, is generally not in a phenotype/disease context matched to corresponding bulk-tissue level data, and certainly not from large numbers of individuals. Moreover, the little single-cell methylomics data that is currently available is very sparse, suffering from low coverage. For instance, the snmC-Seq2 data matrix of brain cell-types that we have analysed in this work has 90% missing entries. The obvious alternative would be to use DNAm profiles of FACS-sorted cell populations in a case/control context, but such data is unfortunately only available for tissues like blood or brain, and in large sample numbers only for blood, which is not the tissue of relevance to EpiSCORE. The reviewer should also bear in mind that methods like CellDMC and TCA not only require cell-type fraction estimates, but they also require a very large bulk-tissue dataset (i.e large numbers of samples) in order to have the appropriate power and precision to detect cell-type specific DNAm changes. Nevertheless, in response to the reviewer's point we have performed the following analyses to alleviate the reviewer's concern: (i) we have used the snmC-Seq2 data of brain cell subtypes to build a pseudo-bulk DNAm reference matrix, and have cross-compared estimated cell-type fractions from this reference with those of our EpiSCORE DNAm-atlas brain reference, in

independent DNAm datasets (Gasparoni et al & Guintivano et al) profiling purified brain cell-types. The result is shown below, which has been added as panels e & f of a new Fig.4:

e)

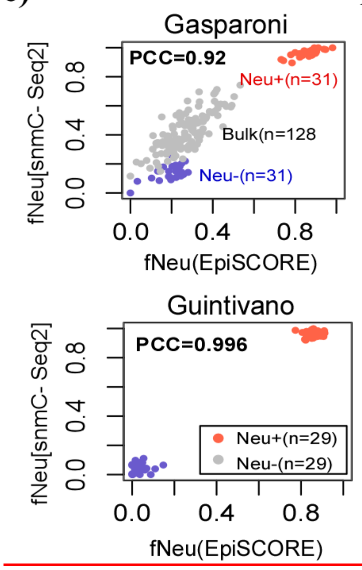

f)

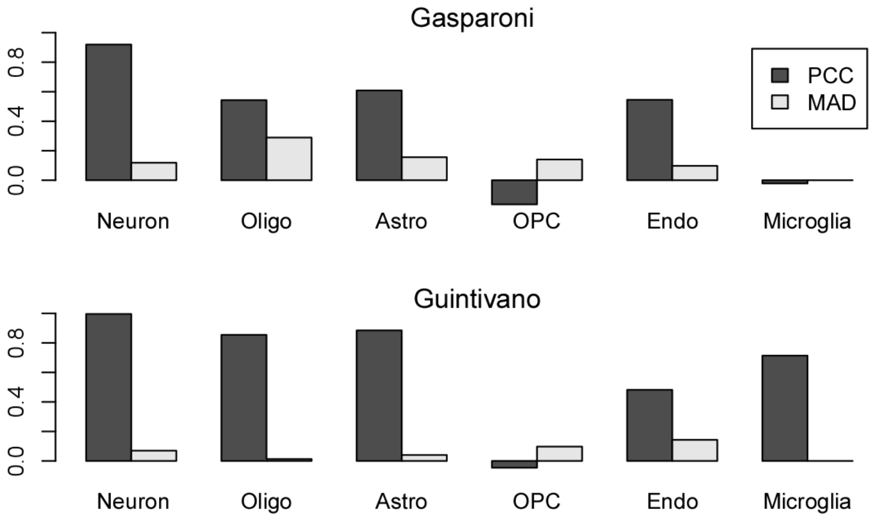

As we can see, there is excellent agreement, thus providing a strong validation. To the left we provide a scatterplot of the estimated neuronal fractions derived from the two DNAm reference matrices in FACS sorted neuronal (Neu+) and non-neuronal (Neu-) populations (Gasparoni & Guintivano), as well as in bulk frontal cortex tissue (Gasparoni only). The Pearson Correlation Coefficients (PCC) are very high. To the right we display the Pearson Correlation Coefficient (PCC) and Median Absolute Deviation (MAD) between the snmC-Seq2 and EpiSCORE derived cell-type fractions for each brain cell-type. The most important measure to consider here is MAD, since for a cell-type that displays very little variance (e.g. those cell-types present in very low proportions), the corresponding PCC value is unlikely to be large or may even be negative, even if the MAD between the two methods is excellent and very small. As we can see, in both Guintivano and Gasparoni, the MAD is generally less than 0.2 and often also less than 0.1, suggesting good agreement of our EpiSCORE derived estimates with those from the snmC-Seq2 derived brain reference.

In another analysis, we directly compare the DNAm values for neurons, as given in the EpiSCORE and snmC-Seq2 DNAm reference matrices, to the Neu+ FACS-sorted derived

DNAm values, in 3 separate datasets profiling Neu+ samples (this data is shown in a new Supp.Fig.S18, displayed below for your convenience):

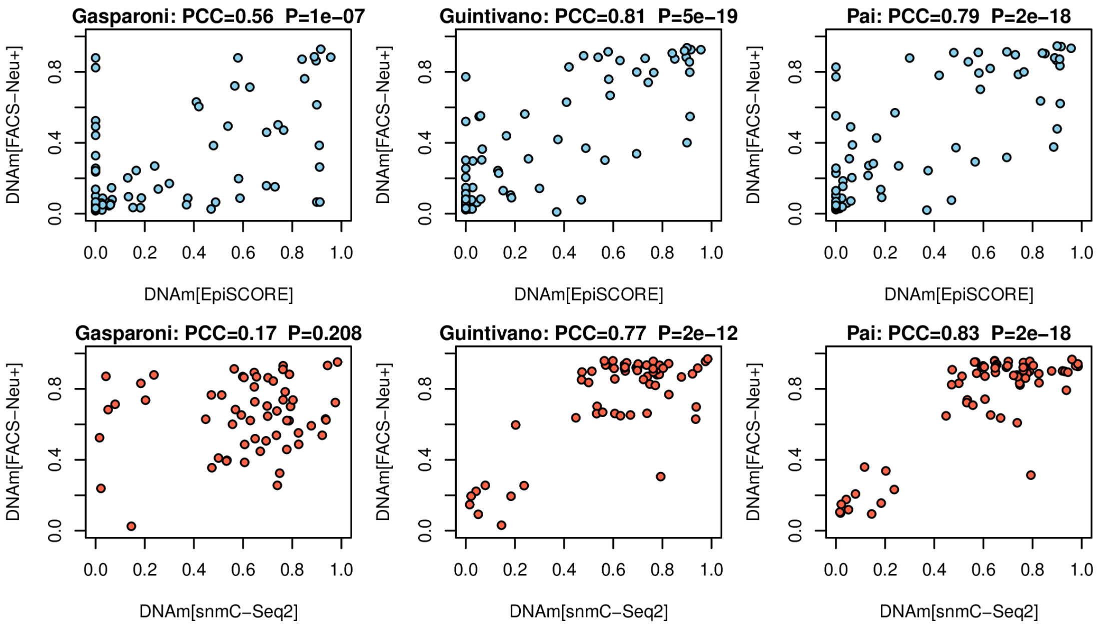

Supplementary Figure 18: Direct comparison of the neuron DNAm reference profiles with DNAm profile of FACS-sorted neuron samples. Top row compares the DNAm profile for neurons as given by the EpiSCORE derived DNAm reference matrix (x-axis) against the DNAm profile obtained by averaging FACS-sorted Neu+ samples (y-axis) from 3 different Illumina DNAm studies (Gasparoni et al, Guintivano et al and Pai et al). Pearson Correlation Coefficient (PCC) and P-value are given. Bottom row is similar to top-row but now using the neuron DNAm profile from the snmC-Seq2 derived DNAm reference matrix.

What this new Supp.Fig.S18 shows is that the agreement (as measured by a Pearson Correlation Coefficient (PCC)) between the DNAm values for neurons in the DNAm reference matrix with the corresponding DNAm values in FACS-sorted neuron samples is stronger when considering the EpiSCORE DNAm reference matrix (top-row in above figure) as compared to the snmC-Seq2 derived one (bottom-row). This suggests that the high sparsity of the snmC-Seq2 data is a limitation and that our EpiSCORE DNAm reference matrix for brain is a more accurate and reliable one.

Comment 3. Another question I had is that the authors only consider CpGs that are both proximal to the TSS of marker genes and strongly anticorrelated with the expression of those genes, to build DNA methylation reference libraries. While I understand and completely agree with the second condition (e.g., that the CpG site must exhibit a strong correlation with expression), otherwise the proposed imputation procedure falls apart, why restrict only to CpGs that are proximal to the TSS of the gene? Why not just consider CpGs that a located in/near the gene of interest and that exhibit strong correlation with gene expression? Seems like there might be a missed opportunity here in building more robust DNA methylation libraries, but perhaps I'm missing something.

Response: The reviewer has raised an excellent question. According to our Illumina-beadarray centric analysis in Jiao Y, Teschendorff Bioinformatics 2014, among all probes/CpGs that map close to a gene, the most predictive probes are those mapping close to the TSS. In fact, there is a substantially stronger association for these probes compared to probes mapping to the 5'UTR, further upstream from the TSS, or within the gene body, with only probes mapping to the 1st Exon demonstrating comparable strength. We did in fact explore using 1st Exon probes too, but results did not change markedly. What may lead to a significant improvement is to use probes/CpGs that map to enhancers linked to the gene in question, and indeed this is a question we already explored in our Genome Biol.2020 paper for the case of whole blood. In the case of whole blood there is a need to distinguish more similar cell-types from each other (e.g. CD4+ and CD8+ T-cells), and here we found that using enhancers in addition to promoters led to a significant improvement. However, blood is not the tissue where we need a tool like EpiSCORE since generating DNAm profiles for FACS-sorted purified cell-types is possible and has been done. In future, we would want to further improve the cellular resolution of the DNAm reference matrices built here. To this purpose, it is clear to us that probes/CpGs in the enhancer regions will need to be incorporated, in order to better distinguish closely related cell-types from each other (e.g. different endocrine subtypes in pancreas). Linking enhancers to genes, although extremely challenging, could be addressed with recent cell-type specific enhancer-promoter mapping studies. We are planning a future improvement of the EpiSCORE method where we will do this, but this would clearly go beyond the scope of this MS and resource. In response to the reviewer's point, we have now added a paragraph to Discussion, mentioning

some of the limitations of our current DNAm-atlas, and in particular that further improvements could be achieved by imputing DNAm at enhancer regions associated with marker genes.

Comment: 4. The final point that I have is that the Discussion section doesn't explicitly acknowledge the limitations of this study. No study, no method, no resource, etc., is without its limitations and I feel that it is important for the authors to shed light on the limitations of their resource and the study as a whole.

Response: We absolutely agree that there are limitations, and by no means did we imply that our resource does not have any. In response to this point, we have now included a paragraph discussing the current limitations of this resource.

Reviewer #2:

General Comment: DNA methylation is being studied extensively as a potential biomarker for human diseases, and one of the most important problems is how to account for cell type composition within bulk tissue samples. The Teschenforff group has developed several mathematical models for reference-based deconvolution of DNA methylation data, including CellDMC which identifies disease-associated methylation within a specific cell type in the mixture, and EPISCORE, which exploits the high resolution cell type clusters available from single-cell RNA sequencing to impute cell-type specific DNA methylation values. These are important tools, given the general lack of availability of DNA methylation reference data derived from either single-cell or FACS-sorted methylation sequencing. The Teschendorff group published the EPISCORE algorithm in Teschendorff et al. 2020 (Genome Biology), and validated for lung and breast cell types. They also showed how it could be combined with CellDMC to identify cell type specific differences in bulk lung tumor tissue. This new manuscript creates similar imputed reference atlases for 11 additional tissue types, developing a pipeline

for collecting scRNA-seq data and validating cell type clusters, then imputing DNA methylation references. They validate their deconvolution estimates using non-methylation based deconvolution methods on TCGA tumor data, as well as methylation profiles of FACS-sorted samples from individual studies of tissues of healthy individuals. They use this new atlas with EPISCORE and CellDMC make several interesting findings about cell type composition and DNA methylation changes in melanoma, olfactory neuroblastoma, and schizophrenia.

Response: We would like to thank the reviewer for taking time to read and evaluate our MS. We are delighted with the reviewer's positive feedback and for this reviewer to have recognized the value of the resource as well as the many validations and interesting findings we have obtained across a broad range of diseases including olfactory neuroblastoma, aortic dissection, melanoma and schizophrenia.

Comment: While most of the elements of this work were already present in the 2020 Genome Biology paper, this new manuscript represents a significant amount of work to standardize and validate the process of collecting additional cell types. Additionally, all the data and R code are publicly available on github and figshare, making this a useful resource. My main concerns are about several tissue types that perform poorly, and how that should be handled in the quality control pipeline.

Response: We thank the reviewer for recognizing the important value that our DNAm-atlas resource will bring to the community. The reviewer is right in pointing out that for a few tissues (e.g. colon, kidney), the validation on the RNA-Seq side was not as strong as we would have liked. In response to this, we have now regenerated the expression reference matrices for kidney and colon using either new recently generated and higher quality scRNA-Seq datasets, or by lowering the cellular resolution (i.e. by combining highly similar cell-types into one broader cell-class). Validation accuracies are now much higher (see further below). In addition, we now also provide a new Suppl.Table.6 ranking tissues by overall validation performance and extent of validation.

Comment: The manuscript was clear and properly referenced.

Response: We thank the reviewer for emphasizing this, as we did indeed put a lot of effort into it.

Major concerns.

Comment: My major concern has to do with quality control, since the tissue scRNA-seq data used to build the atlas comes from diverse sources and sequencing techniques, which may present consistency problems for the framework. Here are two examples:

- The main systematic validation uses TCGA multi-omic data to compare EPISCORE to other methods of deconvolution (most importantly, RNA-seq based) in Fig 2a-b. The KICH cancer type is the main outlier, which has poor correlation between EPISCORE and all other deconvolution methods. In looking at the Kidney cell types in the imputed Atlas (Fig S7), it does look like kidney has a relatively small number of features, and a lot of confusion between cell types. Should this tissue type be excluded due to low quality? Should you implement minimum quality standards so that users don't use poor models?

Response: We thank the reviewer for raising this good point. First of all, let us clarify that in Fig.2a-b, there are in fact 3 different kidney cancer types: KIRC, KIRP and KICH, and that for all 3 we are using the exact same kidney DNAm reference matrix. For KIRC and KIRP, validation is excellent, suggesting that the weaker validation in KICH is not necessarily a quality-issue of our kidney DNAm reference matrix. In fact, there are two other more likely explanations why validation in KICH is weaker: the P-value for KICH would be expected to be less significant simply because the correlations were estimated across a relatively smaller number of samples (n=66). The number of samples for KIRC (n=297) and KIRP (n=195) was much greater. A second potential reason is that KICH is a much rarer kidney tumor-type that exhibits substantial morphological variance, and so it could well be that our DNAm reference matrix is not applicable to this cancer-type, in the same way for instance that a lung DNAm reference matrix without a lung neuroendocrine cell-type would not be applicable to lung neuroendocrine cancers.

Nevertheless, we also agree with the reviewer that the relatively low validation accuracy of the kidney expression reference matrix is a concern. In response to this, we have now regenerated the kidney DNAm reference matrix at a lower cellular resolution (4 cell-types instead of previous

6), by defining a broader tubule epithelial cell class, and using this coarser resolution reference matrix we obtain much better validation performance in the independent scRNA-Seq dataset, as shown in the figure below, which has been included into a revised Supp.Fig.S7:

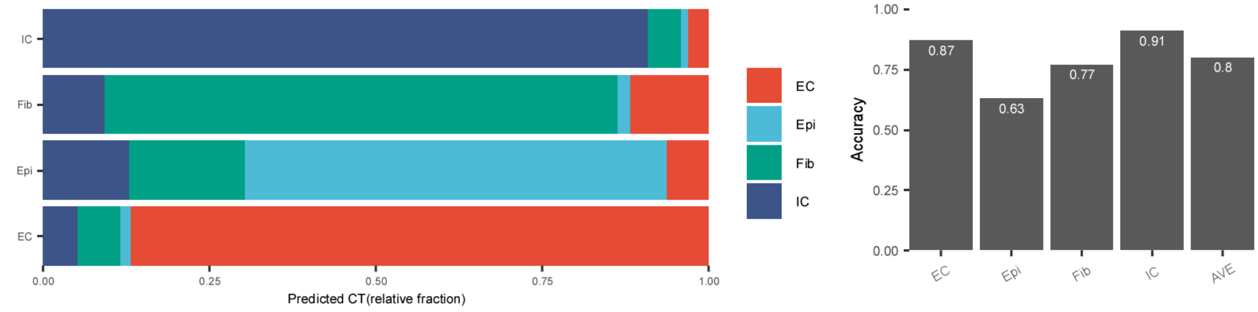

With this new kidney DNAm reference matrix, the estimates of tumor purity and total immune-cell fractions in KICH also improve, as shown in a new Fig.2a-b, which for convenience we display below:

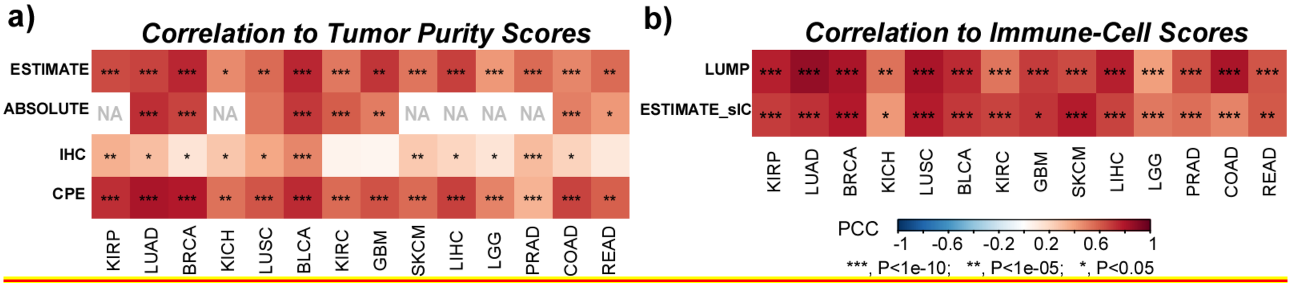

In response to the reviewer's point, we have now also added a new Supplementary Table 6 ranking the tissue-specific scRNA-Seq and DNAm reference matrices by their validation performance and validation extent. We believe that this ranking of tissues will be very useful to highlight those which we believe are most reliable.

- The gold standard validation is against FACS-sorted cell types profiled using the DNA methylation Infinium array. The authors provide four such validations - hepatocytes in liver, neuron vs. glia in brain tissue, Dermis vs epidermis in skin, and endocrine vs. epithelial cell types in pancreas. EPISCORE performs relatively well on the first three , which are shown in

main figures, but performs poorly on the 4th, which is shown only as a supplemental figure. Despite the fact that all QC metrics appear to look fine for the pancreatic scRNA-seq clusters and imputation (Fig S6), there is significant confusion between ductal and endocrine cells in multiple FACS-sorted samples from different sources, and confusion between acinar and ductal cells in another (Fig S17). So where is this problem coming from, is it the scRNA-seq data, the imputation method, or neither? Was it because the imputation was based on whole-genome data and the test data was on the array platform? Should this be used to omit the pancreatic model from the Atlas? This is important because the main value of this work is for the resource of tissue specific models provided and the reliability.

Response: The reviewer has raised an excellent point. As far as Fig.S17a is concerned, the first 3 panels display a very small number of acinar (n=3), beta (n=3) and ductal (n=4) samples from Moss et al Nat Commun 2018. There is surprisingly little information provided in this publication as to how these samples were generated. We must assume that these are FACS-sorted cell populations, yet purity does not seem to have been assessed. In our experience, given the relatively small numbers of samples and given that FACS-sorted cell populations may not always be of high purity, we were not expecting big differences in the estimated cell-type proportions. What the EpiSCORE fractions do demonstrate is that there is broad consistency. As far as the other two panels in Fig.S17a are concerned, these display the estimated fractions in Langerhan islet preparations, which once again don't need to be pure. The only expectation here would be that these islet preparations would exhibit an enriched fraction for endocrine cells, as correctly predicted by EpiSCORE (of note by enrichment we here mean a relatively high fraction of endocrine cells). In our opinion, the data being used for validation in Fig.S17a is the main limitation since the purity of these validation samples is questionable, and therefore this does not necessarily reflect a quality issue of the pancreas DNAm reference matrix. In support of this, the **data shown in Fig.S17b, as well as in Fig.4b,c,d,f demonstrate that the pancreas DNAm reference matrix is making the correct predictions: PNETs derive mostly from alpha and beta-cells, PAAD derives from ductal cells, and most importantly, EpiSCORE has correctly identified misdiagnosed PAAD cases, which are in fact PNETs.** For this reason, we would not remove the pancreas DNAm reference matrix from this work, because the overall evidence is in favour of this reference matrix being useful.

Comment: The issues with heterogeneity of underlying scRNA-seq data and different quality levels beg the question of whether this approach would be more reliable using a single large scRNA-seq dataset based on a common technology and analysis methods. Such a dataset became available from GTEx/Broad Institute just before (https://doi.org/10.1101/2021.07.19.452954). I realize this dataset came too late for you to analyze, but I think it's important to comment on how this will improve quality, and how quality of the underlying models can be better quantified/validated.

Response: The reviewer has raised another excellent point. However, we opine differently in that use of a single large scRNA-Seq atlas would be disadvantageous, <u>because such a resource is unlikely to have generated high-quality tissue-specific datasets for *all* tissue-types.</u> Indeed, based on our experience analyzing data from such resources like the Mouse Cell Atlas or the Human Cell Landscape (HCL), we observe that the quality of individual tissue-specific datasets varies a lot between tissue-types, which is not entirely surprising since each tissue requires tailored experimental protocols that are also variably suited to different technologies. For instance, **if we take the HCL, which is a single large scRNA-Seq multi-tissue atlas generated with a uniform technology, this would be problematic because (i) for skin no scRNA-Seq dataset was generated, (ii) for liver, the scRNA-Seq atlas failed to capture cholangiocytes, an important component of the liver epithelium, (iii) for pancreas, the scRNA-Seq atlas failed to capture gamma and delta endocrine cells, two of the 4 endocrine cell subtypes, and (iv) for brain, very few neuron cells were captured, only yielding relatively few neuronal marker genes.** These 4 tissue-types are important ones since for these we have DNAm datasets that serve for objective validation of the derived DNAm reference matrices. Thus, **it is very clear from this, that the use of a single multi-tissue atlas like HCL would not allow us to construct complete and reliable DNAm reference matrices for many tissue-types**. To make this point clear, <u>we have now compared performance of our DNAm-reference matrices for brain and heart to those we would have derived had we used the HCL scRNA-Seq multi-tissue atlas. The results in the validation DNAm datasets (displayed below) clearly demonstrate that our DNAm reference matrices perform better, demonstrating clearer separability of the FACS-sorted samples in the case of brain, and</u>

in human aorta correctly predicting the increased macrophage to fibroblast ratio in aortic dissection. This figure has been included as a new Fig.3:

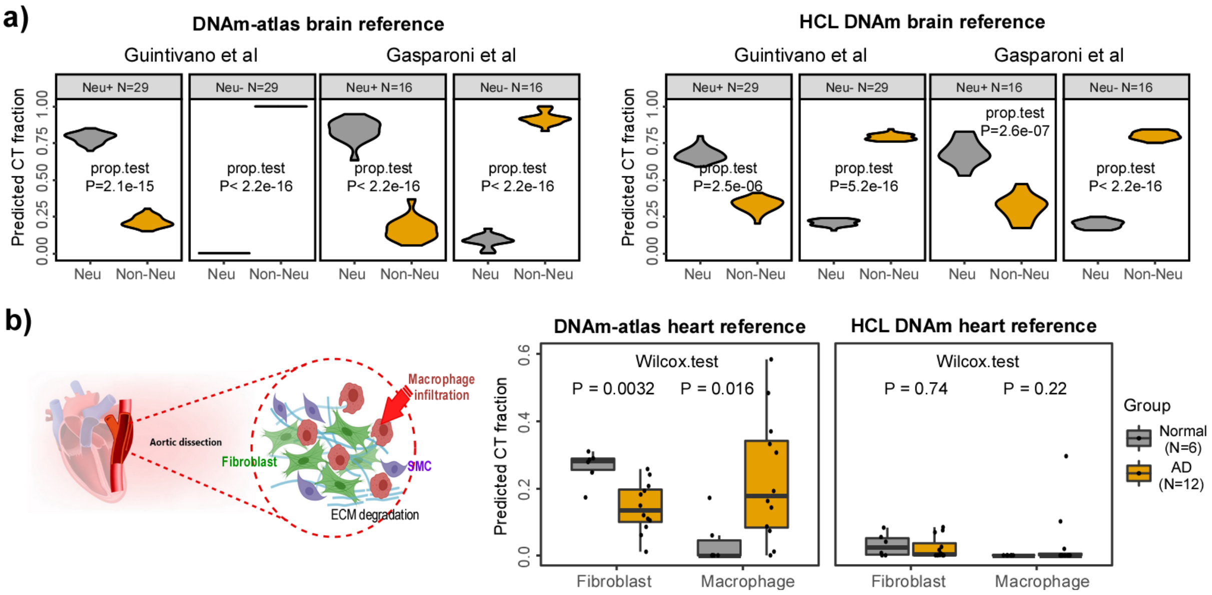

Figure-3. Comparison between DNAm-atlas and HCL-derived DNAm reference matrices. a) *Violin plots depicting predicted cell-type (CT) fractions (y-axis) of neuronal (Neu+) and non-neuronal (Neu-) samples from Guintivano and Gasparoni et al DNAm datasets (in Gasparoni only control samples were included), using the brain DNAm reference matrix from our DNAm-atlas (left) and an analogous one built by starting out from the HCL scRNA-Seq brain dataset (right). Predicted CT fractions are shown for neurons (Neu) and for all other brain cell subtypes combined (Non-Neu) (x-axis). In each case, we assess separability of the Neu and Non-Neu fractions using a test of proportions with the associated P-value shown in plots.* ***b)*** *Aortic dissection is characterized by an increased macrophage infiltration and a concomitant degradation of the extracellular matrix (ECM) (i.e. a reduction in fibroblasts). Boxplots display the predicted CT fractions (y-axis) in a 450k DNAm dataset profiling 6 normal and 12 aortic dissection (AD) samples, obtained using either the heart DNAm reference matrix from our DNAm-atlas (left) or an analogous one built from the HCL heart scRNA-Seq dataset (right). Here we compare fractions between normal and AD samples using a one-tailed Wilcoxon rank sum test.*

Thus, we conclude that only if the common technology were optimally designed for each particular tissue-type, e.g. in providing an unbiased catalogue of all major cell-types, in sufficient

numbers and at sufficient read depth for each one of them, would such a resource be marginally advantageous. The new single-cell resource from GTEX the reviewer is pointing towards has to our knowledge not yet been formally published, and is only available as a preprint which came out at the same time we submitted this MS for publication.

It should also be clarified that the use of a single large scRNA-Seq atlas would only really be necessary if there was a need for us to cross-compare between tissue-types. However, in our case, we don't need to cross-compare between tissue-types, which is a significant advantage as adjusting for batch effects across tissues is problematic. In the approach taken in this MS, a given scRNA-Seq dataset is only being used to generate a corresponding tissue-specific gene-expression reference matrix, i.e. effectively a task of identifying differentially expressed genes where fold-changes are as big as possible. Indeed, our expression reference matrices are effectively binary entities (expressed/not expressed), from which we then impute the corresponding tissue-specific DNAm reference matrices. Hence, the scRNA-Seq data is being used at a level (binary ON/OFF) which also makes it quite insensitive to the underlying technology. In summary, the main limitation for the resource is certainly not the use of one or multiple scRNA-Seq technologies, but the actual DNAm imputation method, where we do anticipate that future significant further improvements will be possible.

In response to the reviewer's excellent point, we have now added a new subsection and the new Fig.3 above to convey the message that using a single multi-tissue scRNA-Seq atlas resource (HCL) to generate the expression and DNAm reference matrices, leads to worse performance in the validation DNAm datasets, as well as presenting a major limitation in that generation of DNAm reference matrices for tissues like skin or liver is not even possible.

Minor point: Melanoma is shown in Fig 2 and then again in Fig 6. It is confusing and would be better shown in a single figure/section

Response: We appreciate the reviewer's point, but we don't see this as a problem, since Fig.2 is dedicated to demonstrating validations, whilst Fig.6 is dedicated to demonstrating how novel insights can be gained through application of the DNAm-atlas. We felt that presentation had to be in this way since a method needs to be validated first in the context of multiple tissue-types, with the applications generating novel insight following the validation.

Reviewer #3:

General Comment: In their manuscript, Zhu et al. present methodology for reference-based deconvolution of DNA methylation data as well as an atlas of deconvoluted DNA methylomes. The novelty of their approach is that reference DNA methylation profiles are not obtained experimentally, but imputed based on available resources of bulk or single-cell gene expression measurements. This has high significance for the field, as reference DNA methylation profiles of major cell types remain rather scarce, whereas the number of cell atlases that are based on single-cell transcriptomic assays is growing rapidly. Overall the proposed approach for atlas construction is original and is based on solid assumptions about strong association between expression levels of cell type-specific marker genes and DNA methylation levels of their promoters.

Response: We sincerely thank the reviewer for taking time to evaluate our manuscript. We are delighted that the reviewer recognizes our work to be of "high significance for the field", and for the significant originality in the methodological approach.

General Comment: Although the computational method EPISCORE underlying current work was published earlier (Teschendorff et al., Genome Biology, 2020), in the current manuscript the authors apply it to variety of datasets and complex biological systems to clearly demonstrate its benefits and provide a resource of imputed DNA reference matrices for subsequent studies. The authors creatively designed numerous validation experiments to prove the feasibility and biological meaningfulness of obtained results. Overall, the framework appears to generate robust cell type estimates in multiple tissues that potentially lead to novel disease-relevant insights. Provided source code and example analyses could be run and reproduced seamlessly. Nevertheless, in my opinion there are several major gaps in validation analyses summarised in several points below, in particular the absence of clear-cut comparison to an appropriate ground truth.

Response: We sincerely thank the reviewer for recognizing that we have come up with creative and numerous ways to validate our DNAm reference matrices. This was indeed one of the most

significant challenges, as for tissues other than blood it is extremely difficult to find data where such validation analyses can be performed. Indeed, we need to stress here that blood is a unique tissue, for which highly accurate DNAm reference matrices already exist. Purpose of our DNAm-atlas is to provide DNAm reference matrices for solid tissues.

Specific Comment 1): During the validation of the tissue-specific reference gene expression matrices the prediction accuracy in independent scRNA-seq datasets was sometimes rather low for certain tissues and cell types (e.g. skin). Could this ambiguity result in wrong or biased composition estimates and lead to erroneous findings? Can authors suggest an approach to measure and mitigate the impact of these inaccuracies?

Response: The reviewer has raised an excellent point. The overall validation accuracy for the skin mRNA expression reference matrix was 0.75, which admittedly is lower than the typical >0.9 accuracy displayed by most of the other tissues. However, in the case of skin this could reflect issues with the validation scRNA-Seq dataset, and may not necessarily reflect the quality of the expression reference matrix, as indeed for skin the validation of the DNAm reference matrix, as shown in Fig.2c,d,e, is very good. In our sincere opinion, the reviewer's concern would apply more to tissues like colon and kidney for which the validation accuracies were just over 0.5. In response to the reviewer's point, we have now regenerated the expression and DNAm references for colon and kidney using new recently generated and higher-quality scRNA-Seq datasets, or by lowering the cellular resolution of the previously used ones. The new validation accuracies for kidney, which are substantially higher, are shown below (included in new Supp.Fig.S7):

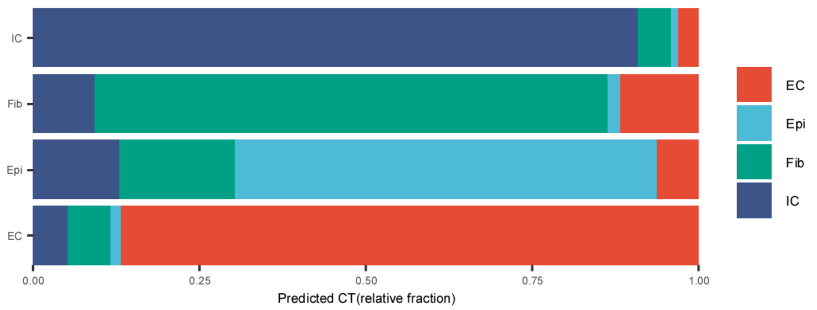

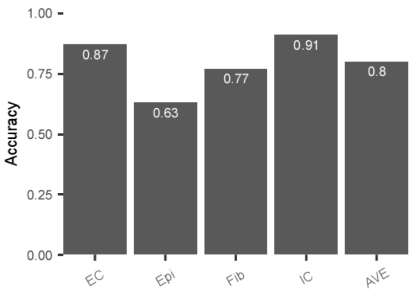

The right panel in the above figure thus demonstrates **that the average classification accuracy for the kidney expression reference matrix is 0.8,** up from 0.52. With this new kidney DNAm reference matrix, the estimates of tumor purity and total immune-cell fractions in KIRC, KIRP and specially KICH also improve, as shown in a new Fig.2a-b, which for convenience we display below:

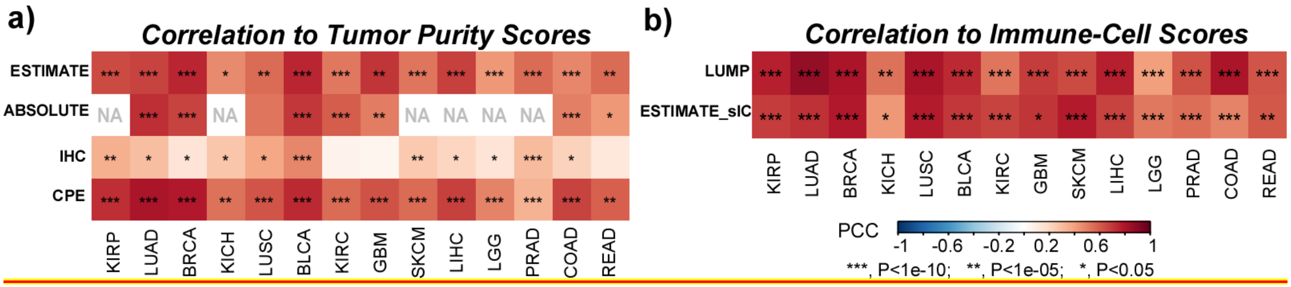

For colon, we have also regenerated a new expression reference matrix, which now also achieves an overall accuracy of 0.8 in the independent scRNA-Seq dataset. We display the revised SuppFig. (new Supp.Fig.S10) for colon tissue below:

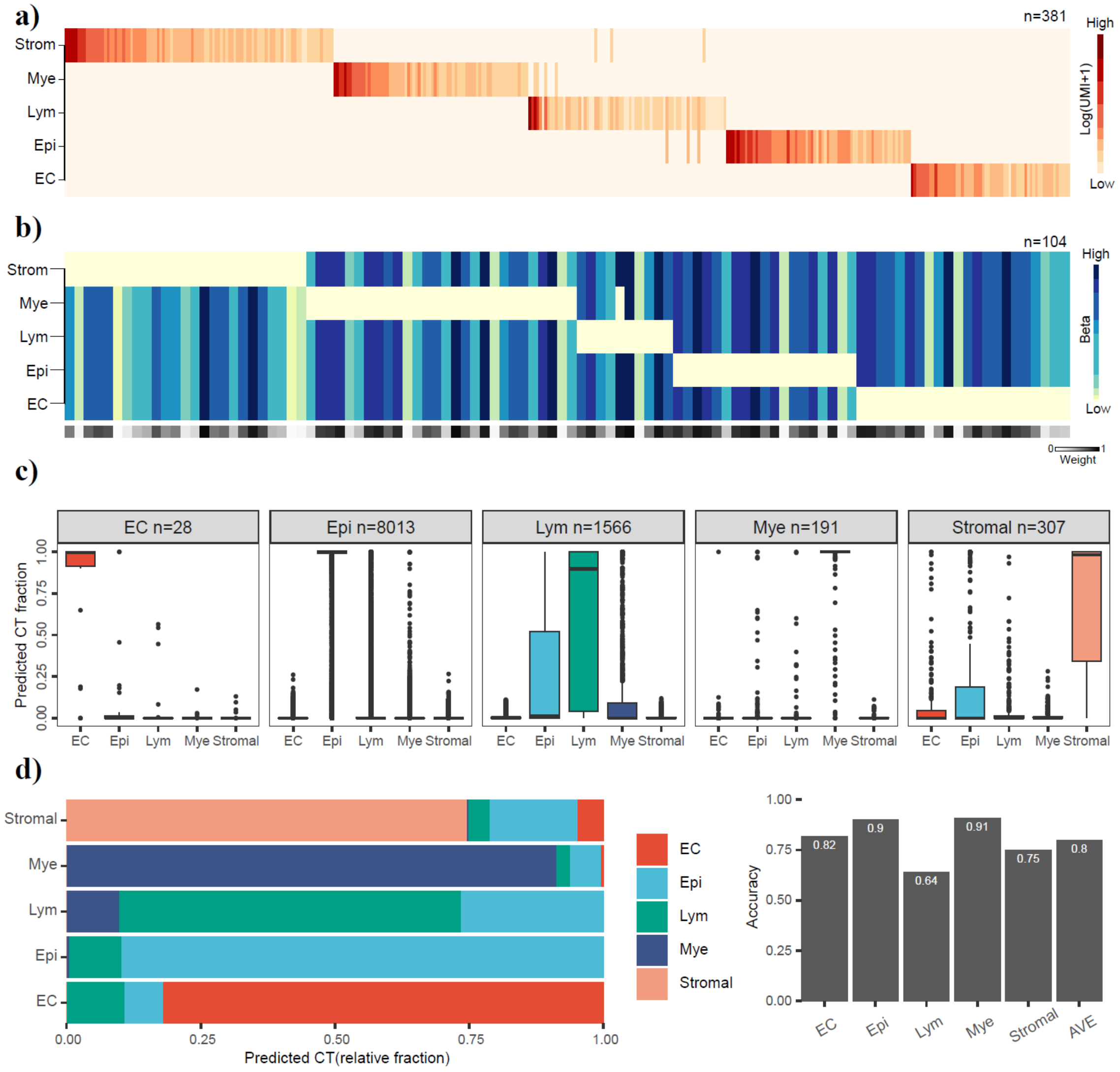

Focusing on panel-d) (right panel) in the above figure, we can see that the validation accuracy is significantly higher compared to the previous version. In addition, we have also added a new Supplementary Table 6, ranking tissues by their validation performance and validation extent. This will help readers assess which tissue-specific DNAm reference matrices are most reliable.

Specific Comment 2): For two independent scRNA-seq datasets used to construct reference gene expression matrices, what were the criteria to select the primary dataset for marker selection and the secondary for validation? Could authors perform a round of reciprocal

construction-validation procedures and compare the obtained markers and cell type estimation accuracy to their original results?

Response: The reviewer has raised a good point. The criteria for selecting the primary set was (i) the number of distinct cell-types profiled, as well as (ii) the number of cells within each cell-type. In effect, for the reference matrix construction it was important that the primary set contained as many of the relevant cell-types as possible and also in sufficient numbers to ensure adequate power to detect a sufficient number of marker genes for each cell-type. For most tissues it was a clear choice as to which scRNA-Seq dataset should be primary, and which one should be used for validation. However, for a few tissues (e.g. liver) the choice was more ambiguous. In response to the reviewer's point, we have now performed the reciprocal analysis for liver, results of which are displayed below:

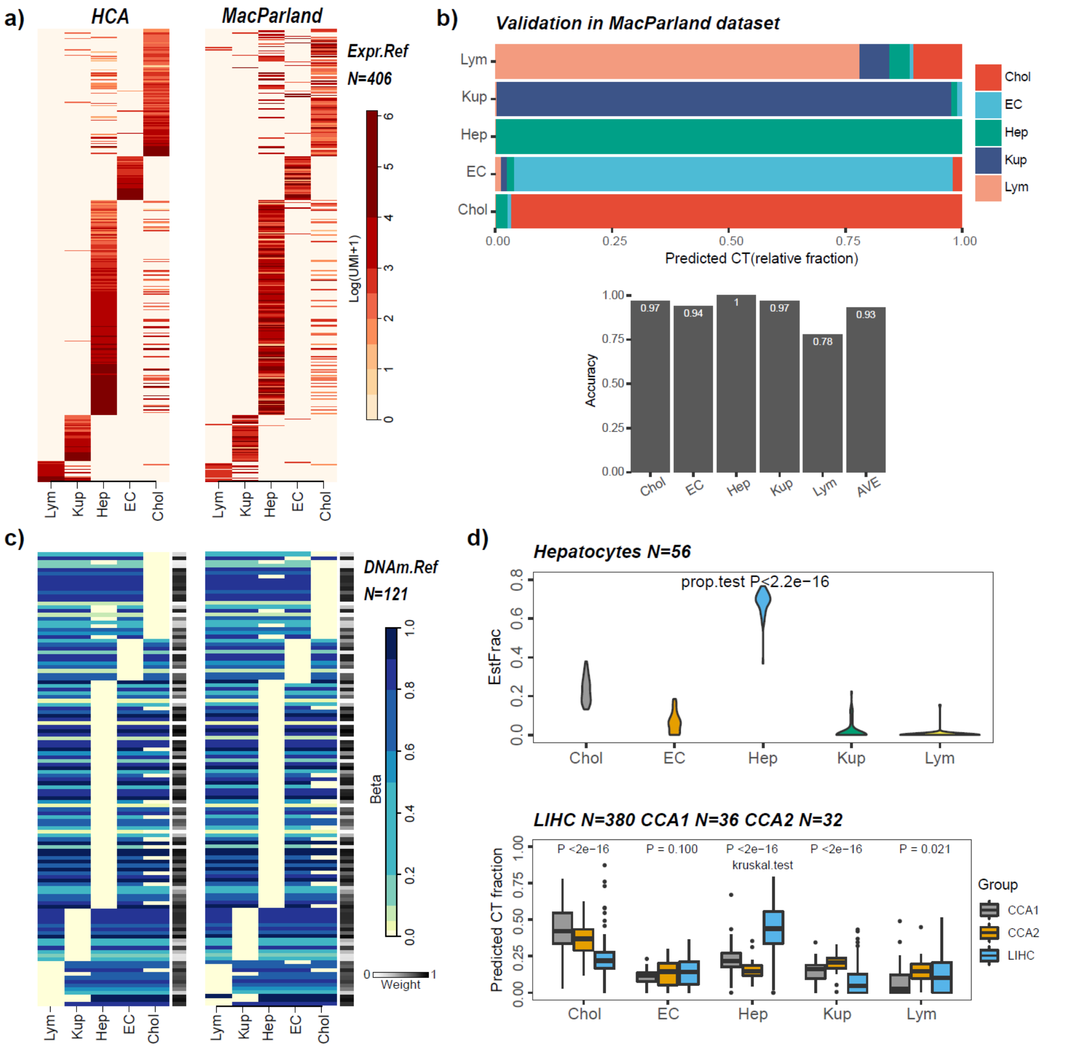

***Figure legend:* a)** *The two expression reference matrices for liver tissue derived from two scRNA-Seq liver atlases from the Human Cell Landscape (HCL) and MacParland et al. Shown are the expression profiles for the same 5 cell-types and a common set of 406 overlapping marker genes.* **b)** *Predicted cell-type fractions and overall classification accuracy as obtained by applying the expression reference from HCL to the scRNA-Seq dataset from MacParland.* **c)** *The two imputed DNAm reference matrices derived from the corresponding expression reference matrices in a), as defined over a common set of 121 marker genes.* **d)** *Validation of the HCA liver DNAm reference matrix in independent DNAm datasets profiling hepatocytes (top panel) and TCGA hepatocellular carcinoma (LIHC) and cholangiocarcinoma (CCA1/2). To be compared with Fig.2 where results are presented for the liver DNAm reference matrix derived from MacParland.*

This figure clearly demonstrates that results are very robust. Indeed, it is worth noting the very strong overlap and correlation of the expression and DNAm reference matrices obtained from the original and reciprocal analyses. While we would be happy to include the above analysis in the MS, we feel it might also disrupt the flow and due to the MS already being long, we prefer to let the editor decide if inclusion of this figure as a new Supplementary Figure is necessary or not.

Comment 3). While the validation of imputed DNA methylation reference matrices appears convincing, it is rather indirect. Would it be possible to validate the imputed matrices directly on SCM2 and RMAP datasets (or any other similar dataset with both data modalities, e.g. brain tissue data presented in the manuscript) by comparing them to respective ground truth matrices using correlation or other appropriate distance?

Response: We thank the reviewer for raising this excellent point. Broadly speaking, this validation strategy is what we pursued with the brain DNAm reference matrix, by comparing it to the snmC-Seq2 data in old Fig.3b-c (now new Fig.4). While we do not explicitly compare the matrices to each other, we displayed the data in the form of boxplots (new Fig.4b-c) to show that a marker gene highly expressed in one specific brain cell subtype (for which the imputed promoter DNAm value is ~0), displays low promoter snmC-Seq2 DNAm levels in that same cell-type compared to all other cell-types (Fig.4b). For each marker gene, we thus obtain a t-statistic and P-value. We then plot the distribution of t-statistics over all marker genes of a given cell-type (Fig.4c). This shows that there is a clear trend for the marker genes to display lower promoter DNAm levels in the cell-types they are overexpressed in, compared to all other cell-types. Thus, Fig.4b-c provides a direct validation of the imputed DNAm reference matrix for brain, albeit on a relative scale.

To perform a direct validation of the imputed DNAm reference matrix on an absolute scale is more problematic because of the high sparsity of the snmC-Seq2 data: indeed, the reviewer is perhaps unaware of the fact that approximately 90% of the entries in the snmC-Seq2 are missing due to low read coverage. This means that in deriving a pseudo-bulk DNAm profile for each cell-type (i.e. averaging over the non-missing values in each cell-type), that this pseudo-bulk derived DNAm value is obtained on average from only about 60 cells per cell-type.

Nevertheless, in response to the reviewer's point, we have computed the Median Absolute Deviation (MAD-score) and Pearson Correlation Coefficient (PCC) between the EpiSCORE-imputed and the snmC-Seq2 derived pseudo-bulk DNAm matrix, observing a MAD-value of 0.11 (P<0.0001 as assessed by Monte-Carlo Randomization with 10,000 permutations) and a PCC-value of 0.56 (Correlation test P<2e-16). We display these data in the figure below, which has been added as a new Supp.Fig.S17:

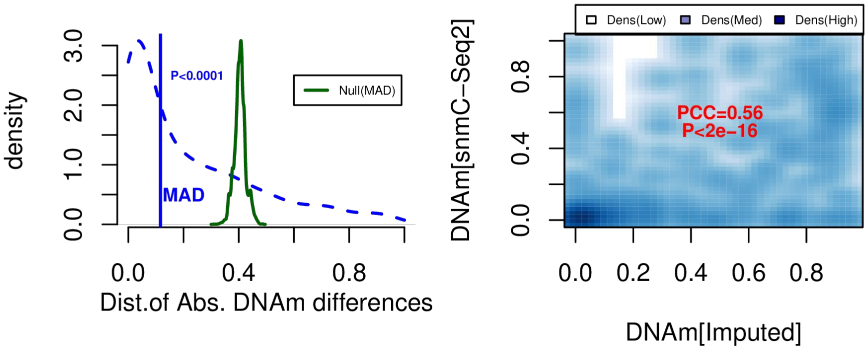

Figure legend: Left panel displays the distribution of absolute differences between the imputed and the pseudo-bulk snmC-Seq2 DNAm data matrices (dashed-curve), with the median absolute deviation (MAD) indicated by a vertical solid blue line. Green curve denotes the MAD values from 10,000 Monte-Carlo randomizations (null distribution). P-value is obtained by comparing the observed MAD value to the null distribution. Right panel is a smoothed scatterplot of the imputed DNAm values and the pseudo-bulk snmC-Seq2 ones. PCC denotes the Pearson Correlation Coefficient and Correlation test P-value is given.

As far as the SCM2 and RMAP datasets are concerned, these can't be used for two reasons: First of all, these datasets were used as part of the imputation, so they don't satisfy the independence criterion required by a validation set. Second, most of the samples from SCM2 and RMAP do not in fact represent purified samples. This of course is not a limitation for finding imputable genes where promoter DNAm and gene-expression are highly anti-correlated, but it does present a limitation for the type of validation the reviewer is suggesting.

Comment 4) Despite that numerous validations of the proposed methodology have been performed, a direct and quantitative comparison to a plausible ground truth is missing. Could the authors use one of the tissues for which both, reference (sc)RNA-seq and DNA methylation

data of the same cell types available, and compare the EPISCORE results to those of any of the standard reference-based deconvolution algorithms (e.g Houseman's method, EpiDISH etc)? The brain tissue data presented in the manuscript appear to be perfectly suitable for this validation. How do proportions of brain cell types in schizophrenia recovered with EPISCORE-imputed DNA methylation matrix compare to those obtained using e.g. the pseudo-bulk scnmC-seq-based reference and how consistent would downstream annotation and interpretation be? Another possibility would be to use data obtained with one of the multi-modal single-cell protocols yielding expression and methylation data from the same cell (e.g. mouse gastrulation atlas in Argelaguet, Nature, 2019).

Response: We thank the reviewer for the excellent suggestions. Although the brain snmC-Seq2 data is very sparse (fraction of NAs is 90%) we agree that we could try to build a pseudo-bulk DNAm reference matrix from it, which we could then apply to independent DNAm datasets from purified brain cell-types or bulk brain tissue, in order to compare results obtained with our EpiSCORE-derived DNAm reference. <u>Below, in the scatterplots to the left, we display and compare the neuron fractions estimated from the snmC-Seq2 derived pseudo-bulk reference matrix to the ones derived from our brain DNAm-reference matrix (EpiSCORE) in the FACS-sorted Neu+ and Neu- samples from Guintivano et al, and the Neu+, Bulk PFC and Neu- samples from Gasparoni et al:</u>

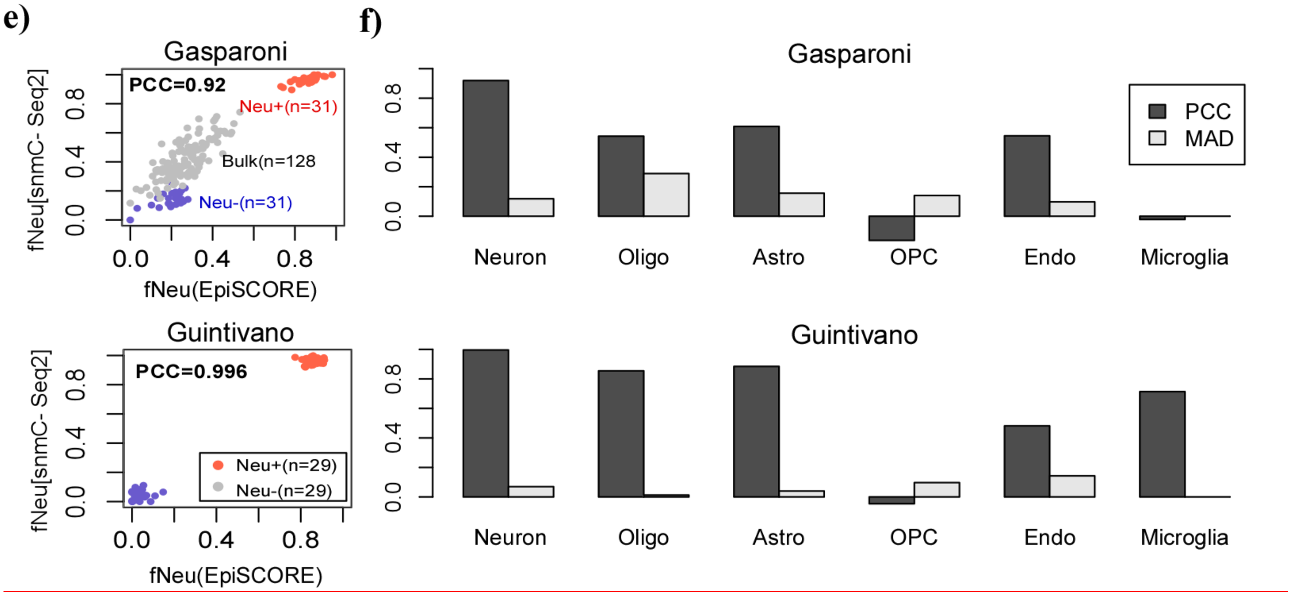

These panels now constitute panels e & f in a new Fig.4, and as we can see in panel e) there is very good agreement. In panel f) we display the Pearson Correlation Coefficient (PCC) and Median Absolute Deviation (MAD) between the snmC-Seq2 and EpiSCORE derived cell-type fractions, separately for each brain cell-type. The most important measure to consider here is MAD, since for a cell-type that displays very little variance (e.g. a cell-type present in very low proportions), the corresponding PCC value is not expected to be large and may even be negative, even if the MAD between the two methods is very small. As we can see, in both Guintivano and Gasparoni, the MAD is generally less than 0.2 and often also less than 0.1, suggesting good agreement of our EpiSCORE derived estimates with those from the snmC-Seq2 derived brain reference.

In another analysis that sheds additional important insight, we directly compare the DNAm values for neurons, as given in the EpiSCORE and snmC-Seq2 DNAm reference matrices, to the Neu+ FACS-sorted derived DNAm values, in 3 separate datasets profiling Neu+ samples (this data is shown in a new Supp.Fig.S18, displayed below for your convenience):

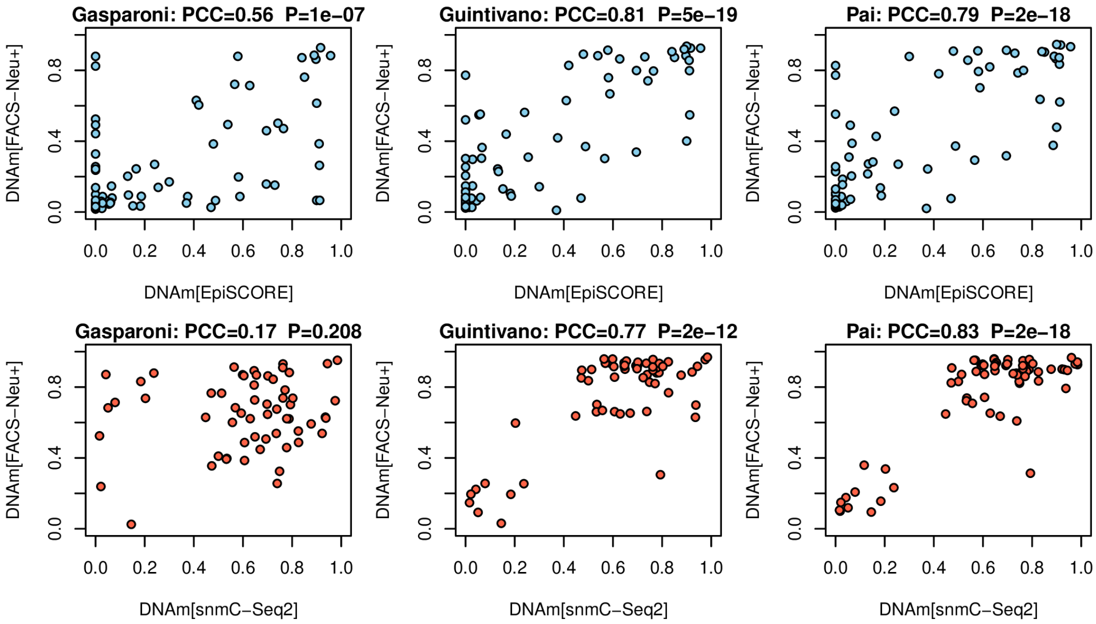

Supplementary Figure 18: Direct comparison of the neuron DNAm reference profiles with DNAm profile of FACS-sorted neuron samples. *Top row compares the DNAm profile for neurons as given by the EpiSCORE derived DNAm reference matrix (x-axis) against the DNAm profile obtained by averaging FACS-sorted Neu+ samples (y-axis) from 3 different Illumina DNAm studies (Gasparoni et al,*

Guintivano et al and Pai et al). Pearson Correlation Coefficient (PCC) and P-value are given. Bottom row is similar to top-row but now using the neuron DNAm profile from the snmC-Seq2 derived DNAm reference matrix.

What this new Supp.Fig.S18 shows is that the agreement (as measured by a Pearson Correlation Coefficient (PCC)) between the DNAm values for neurons with the corresponding DNAm values in FACS-sorted neuron samples is stronger when considering the EpiSCORE DNAm reference matrix (top-row in above figure) as compared to the snmC-Seq2 derived one (bottom-row). This suggests that the high sparsity of the snmC-Seq2 data is a limitation and that our EpiSCORE DNAm reference matrix for brain is potentially a more accurate and reliable one. As far as the mouse gastrulation multi-modal data is concerned this is not only problematic because of the high sparsity of the underlying single-cell DNAm data, but also because it is mouse data, and our DNAm-atlas is aimed at human tissues. We should clarify that the integrated databases SCM2 and Epigenomics Roadmap that we use to identify imputable genes are based on human samples, so all the validations of our DNAm reference matrices need to be in human tissue. In future, it would be important to develop an analogous DNAm-atlas for mouse, specially given that Illumina now has a mouse methylation array, and there will be a great need to perform cell-type deconvolution in mouse tissues, so this is an exciting and necessary project for the future of the epigenomics field.

Comment 5) Although not explicitly emphasized, there is no obvious reason why similar atlases cannot be constructed based on bulk gene expression data, either array- or RNA-seq-based. In case it is indeed possible, rich reference epigenome resources (e.g. BLUEPRINT project for hematopoietic cells) can be used to perform a direct validation as described above.

Response: We appreciate the reviewer's point, but of course there is limited availability of bulk mRNA expression profiles of purified (e.g. FACS-sorted) samples for all cell-types within a given tissue, with the exception of a tissue like blood for which BLUEPRINT has generated mRNA and DNAm profiles for all major purified blood cell-subtypes. Thus, the reviewer's strategy can't be implemented for the solid tissue-types that we are aiming for. Moreover, in the context of blood tissue, we did already attempt a strategy similar to what the reviewer is implying and this was shown in our EpiSCORE 2020 paper, where as proof of principle we imputed a DNAm reference

matrix for peripheral blood mononuclear cells from a corresponding PBMC scRNA-Seq atlas, which we then validated using independent DNAm data from purified cell-types (FACS-sorted cell populations). For convenience, we add the corresponding figure from that paper below:

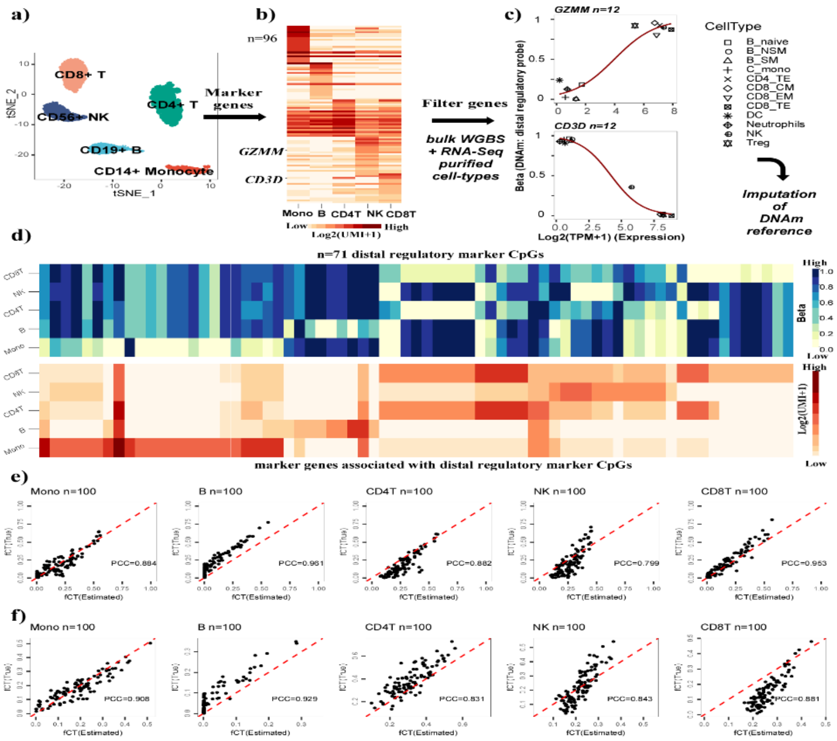

Figure-6 from EpiSCORE 2020 paper: ***Proof of EPISCORE concept in peripheral blood. a)*** *Dimensional reduction and t-SNE embedding of a 10X dataset profiling 68,000 peripheral blood mononuclear cells (PBMCs), depicting the resulting cell clusters, annotated to PBMC cell-types using a bulk RNA-Seq reference matrix for PBMCs.* ***b)*** *The expression reference matrix over 5 main PBMC cell subtypes and 96 marker genes, as constructed from the scRNA-Seq data in a).* ***c)*** *Two examples of marker genes, where differential expression across blood cell subtypes (as assessed using independent bulk RNA-Seq and DNAm data of purified blood cell types), is associated with corresponding differential DNAm at a CpG mapping to a distal regulatory element of the gene. The y-axis labels DNAm at this CpG, x-axis labels the normalized gene expression value, and datapoints are shown for all matching blood cell subtypes. A fit from a logistic regression is shown, which is used to impute DNAm at this CpG from the expression values in the scRNA-Seq reference matrix, after suitably normalizing the expression data.* ***d)*** *Heatmaps displaying the imputed DNAm reference matrix for 71 marker CpGs (top panel) and the corresponding scRNA-Seq reference matrix for X marker genes associated with these 71 CpGs.* ***e)*** *Validation of the imputed DNAm reference matrix using in-silico mixtures of PBMC cell*

types, using Illumina 450k profiles from Reinius et al. For each of the 5 PBMC cell-types we plot the estimated fractions (x-axis) vs. the true fractions (y-axis), for each of 100 in-silico mixtures. Mixture weights were simulated from a uniform Dirichlet distribution. PCC-values are given. ***f)*** *As e), but with mixture weights drawn from a non-uniform Dirichlet distribution, with mean and variance for each cell-type matched to observed data.*

Let us clarify here again that the current manuscript's aim is to provide a resource to the community to enable cell-type deconvolution of solid tissue-types at an unprecedented higher cellular resolution. As such, to perform additional analyses on BLUEPRINT data would not help address this aim.

Comment 6) What are the reasons for applying the proposed framework separate to each tissue? Would it be possible to impute DNA methylation matrices across multiple tissues and use them to estimate cell type proportions in samples of unknown origin? Could the authors test this?

Response: The reviewer raises a very interesting question. The answer as to which strategy is more appropriate largely depends on the downstream application. For the applications we are interested in (e.g. identifying misdiagnosed cancer-cases within a tumor-type, novel prognostic subgroups within a tumor type), it makes more sense to take a tissue-centric approach, as done in our MS. There are a number of reasons for this: First of all, the strategy the reviewer is proposing would make more sense if we can assume that non tissue specific cell-types like endothelial or fibroblast cells have highly similar profiles irrespective of the tissue they are found in. While there is evidence that if we were to do a clustering of say all cell-types within two distinct organs (say lung and brain) that the endothelial cells from lung and brain would cluster together, this only means that they are more similar to each other compared to the other cell-types within these tissues. It does not mean that their profiles are sufficiently similar to equate them. Indeed, there will be critical differences between the endothelial cells found in different tissue-types like lung or brain. Thus, it seems safer to us, to build tissue-specific reference matrices that treat each cell-type in the tissue as a unique cell-type, in other words to make a distinction between say the endothelial cells found in lung from those found in brain. A second reason is that the reviewer's proposed strategy would also require comparisons of scRNA-Seq

datasets across many different tissue-types, generated with potentially different technologies or sequencing depths, which makes quantitative comparisons of expression problematic. While binarizing gene expression into ON/OFF states can partly circumvent this problem (and indeed we have explored this), it is challenging to retrieve enough marker genes from this approach as there would be correspondingly many more cell-types. Alternatively, one could also try to cross-compare tissue-specific scRNA-Seq datasets if these have all been generated within the same large atlas (e.g. the Human Cell Landscape – HCL). However, this strategy leads to worse performance in the applications considered in this MS. For instance, for skin, HCL did not generate any atlas; for liver the corresponding HCL-atlas failed to capture cholangiocytes (an important component of the liver epithelium) not allowing us to apply it to liver cancer diagnosis; for the pancreas HCL atlas, gamma and delta endocrine cells are also absent, which is not ideal for diagnostic applications in pancreatic cancer; and the brain-HCL atlas failed to capture sufficient numbers of neurons not allowing identification of sufficient neuronal marker genes that are imputable, rendering the resulting DNAm-reference for brain unreliable. This clearly shows that it is much better to use high-quality scRNA-Seq datasets tailored for each tissue-type. To make this point clear, we have now added a new subsection and a new Fig.3 where we compare the performance of our DNAm reference matrices to those derived from a common multi-tissue scRNA-Seq atlas resource (in our instance this is the HCL-atlas), which for convenience we display below:

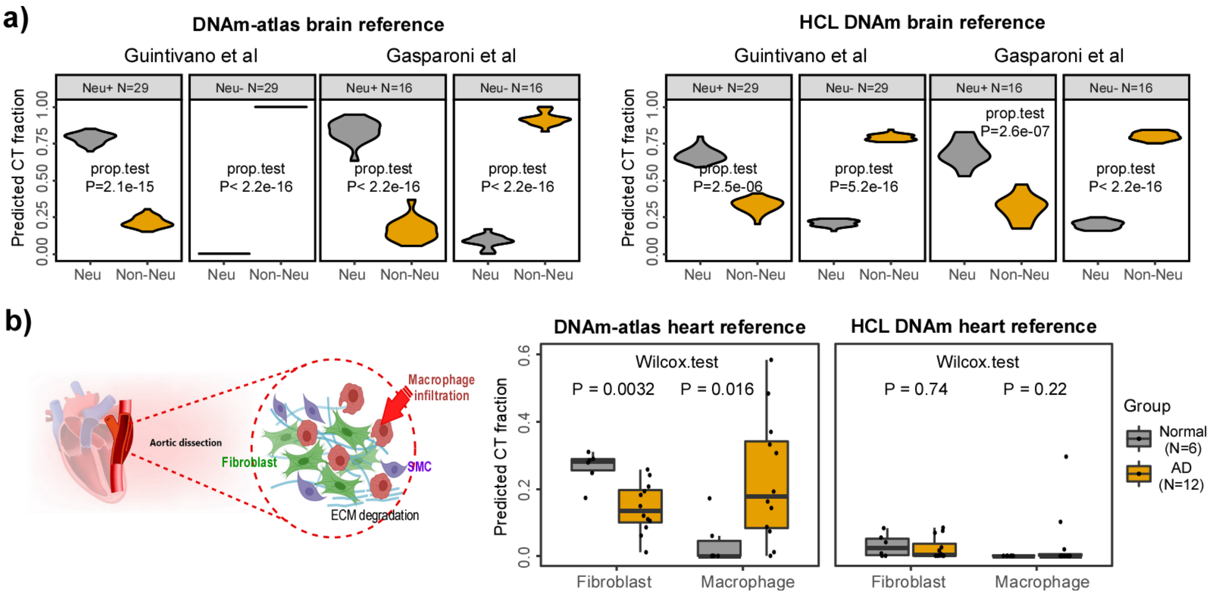

Figure-3. Comparison between DNAm-atlas and HCL-derived DNAm reference matrices. a) *Violin plots depicting predicted cell-type (CT) fractions (y-axis) of neuronal (Neu+) and non-neuronal (Neu-) samples from Guintivano and Gasparoni et al DNAm datasets (in Gasparoni only control samples were included), using the brain DNAm reference matrix from our DNAm-atlas (left) and an analogous one built by starting out from the HCL scRNA-Seq brain dataset (right). Predicted CT fractions are shown for neurons (Neu) and for all other brain cell subtypes combined (Non-Neu) (x-axis). In each case, we assess separability of the Neu and Non-Neu fractions using a test of proportions with the associated P-value shown in plots.* ***b)*** *Aortic dissection is characterized by an increased macrophage infiltration and a concomitant degradation of the extracellular matrix (ECM) (i.e. a reduction in fibroblasts). Boxplots display the predicted CT fractions (y-axis) in a 450k DNAm dataset profiling 6 normal and 12 aortic dissection (AD) samples, obtained using either the heart DNAm reference matrix from our DNAm-atlas (left) or an analogous one built from the HCL heart scRNA-Seq dataset (right). Here we compare fractions between normal and AD samples using a one-tailed Wilcoxon rank sum test.*

These new analyses performed on tissues (brain & heart) where the DNAm reference matrices derived from HCL contained a reasonable number of markers, clearly demonstrate a much better validation performance of our DNAm reference matrices, e.g. we observe much clearer separability of the estimated Neu and Non-Neu fractions in FACS-sorted cells using the DNAm reference matrices derived from separate scRNA-Seq datasets as compared to those derived from a single resource (HCL).

Finally, while we agree that it would be valuable to be able to classify a sample of unknown origin, we see this is as a completely separate question and project, which would require an entirely different approach. For instance, such a question is more interesting in the context of measuring DNAm in cell-free DNA (serum).

Minor points:

Comment: On the minor side, which is however quite important for the statistical soundness of the obtained conclusions, I wonder whether a proportion test perhaps be more suitable for testing the differences of recovered cell type fractions as compared to the Wilcoxon rank sum test used for many analyses throughout the manuscript?

Response: The reviewer has raised a valid statistical question. If we are comparing a cell-type fraction between datasets (e.g. comparing a hepatocyte fraction between TCGA hepatocellular carcinoma and TCGA cholangiocarcinoma as done in right panel of Fig.2f), using a Wilcoxon rank sum test is entirely appropriate. The reviewer is therefore probably referring to the scenario where we are comparing different cell-type fractions within the same dataset (e.g. comparing hepatocyte to cholangiocyte fractions within TCGA hepatocellular carcinomas), where there is statistical dependence or relation between any two cell-type fractions within each sample of the dataset. In this case, we agree that an unpaired Wilcoxon rank sum test would not be strictly speaking correct, although owing to the fact that we have many cell-types (not just 2) this is not going to greatly affect the P-values. For instance, <u>we have checked that the P-value bound shown in the left panel of Fig.2f is correct, since we obtain the same value using a test for proportions (prop.test function in R) or a paired Wilcoxon rank sum test. In response to the reviewer's point, we now compute P-values using a test for proportions, and all results remain unaltered.</u>

Comment: Last but not leasrt, the manuscript is written very clearly, with high accuracy in details, comprehensive and well-readable graphics. Relevant work is referenced appropriately.

Response: We greatly appreciate this positive comment.

Decision Letter, second revision:

Subject: AIP Decision on Manuscript NMETH- RS46537C
Message:

Our ref: NMETH- RS46537C

23rd Dec 2021

Dear Dr. Teschendorff,

Happy Holidays! Thank you for your letters that respond to the concerns raised by the reviewer #2 regarding your Resource paper entitled "A pan-tissue DNA methylation atlas enables in-silico microdissection of human tissue methylomes at cell-type resolution". It has now been evaluated by the editorial team, and we'll be happy in principle to publish the paper in Nature Methods, pending revisions proposed in your response letter to settle the concerns about the pancreas results as well as to comply with our editorial and formatting guidelines.

We are now performing detailed checks on your paper and will send you a checklist detailing our editorial and formatting requirements in about a week. Please do not upload the final materials and make any revisions until you receive this additional information from us. Please expect some delay in receiving the checklists since we are short-staffed in the holiday season.

TRANSPARENT PEER REVIEW
Nature Methods offers a transparent peer review option for new original research manuscripts submitted from 17th February 2021. We encourage increased transparency in peer review by publishing the reviewer comments, author rebuttal letters and editorial decision letters if the authors agree. Such peer review material is made available as a supplementary peer review file. Please state in the cover letter 'I wish to participate in transparent peer review' if you want to opt in, or 'I do not wish to participate in transparent peer review' if you don't. Failure to state your preference will result in delays in accepting your manuscript for publication.
Please note: we allow redactions to authors' rebuttal and reviewer comments in the interest of confidentiality. If you are concerned about the release of confidential data, please let us know specifically what information you would like to have removed. Please note that we cannot incorporate redactions for any other reasons. Reviewer names will be published in the peer review files if the reviewer signed the comments to authors, or if reviewers explicitly agree to release their name. For more information, please refer to our FAQ page.

Thank you again for your interest in Nature Methods Please do not hesitate to contact me if you have any questions.

Best regards,
Lei

Lei Tang, Ph.D.
Senior Editor
Nature Methods

ORCID
IMPORTANT: Non-corresponding authors do not have to link their ORCIDs but are encouraged to do so. Please note that it will not be possible to add/modify ORCIDs at proof. Thus, please let your co-authors know that if they wish to have their ORCID added to the paper they must follow the procedure described in the following link prior to acceptance:
https://www.springernature.com/gp/researchers/orcid/orcid-for-nature-research

Reviewer #1 (Remarks to the Author):

I appreciate the author's attention and time to the comments, suggestions, and critiques from the previous review. While I am mostly satisfied with the authors' responses and the additions they made to their revised manuscript, I have a two lingering comments.

1. In regard to this reviewer's previous comment to "cross-compare cell-specific methylation events/effects identified using this atlas in tandem with cellDMC/TCA, when applied to bulk-tissue methylation data in a solid tissue type, to the results of single cell methylation data in the same tissue type" the authors aptly point out that such an analysis would be challenging given the type of data that would be needed for such a comparison and the corresponding dearth of such data that are available in the public domain. The authors present two additional analyses in their response to "alleviate the reviewer's concern", however these analyses do not directly address the original question. The first analysis (now panels e and f in Fig. 4) shows agreement in the cell type fractions for neuronal (Neu +) and non-neuronal (Neu -) populations while the second analysis (now, Supplementary Figure 18) depicts a direct comparison of the neuron DNAm reference profiles with DNAm profile of FACS-sorted neuron samples. Both analyses are interesting in their own right and relevant to the scope of this paper, they just don't address this reviewer's initial comment, which was to use the cellular landscape estimates from EpiSCORE in tandem with cellDMC/TCA to see if the estimated cell-specific methylation effects are consistent with what is observed purified cell populations or single cell DNA methylation data. I understand that such analysis, at least one that is conducted with a sufficient sample size to ensure statistical rigor, is challenging based on what data are (rather are not) available in the public domain.

Perhaps it is best left at that and described as a limitation of the paper or as an opportunity for future work. One final point on this front regarding the results shown in Supplementary Figure 18. The PCC is the only reported metric in this analysis and I feel that the authors should additionally report the RMSE as I suspect that the PCC is being driven by the clusters of points with high and low methylation values.
2. In the introduction, the authors state, "To address these challenges, a number of reference-based and reference-free cell-type deconvolution methods have recently been proposed". As a matter of record, I feel that the author's should include the Houseman 2012 paper as a reference here as this was the first paper to describe cellular deconvolution in the context of DNA methylation data.

Reviewer #2 (Remarks to the Author):

I am still troubled by the pancreas results. First, regarding the FACS-sorted validation samples from Moss et al. 2018 (acinar, beta, and duct cells). You responded "There is surprisingly little information provided in the Moss et al. 2018 publication as to how these samples were generated. We must assume that these are FACS-sorted cell populations, yet purity does not seem to have been assessed". It was actually easy for me to find this information in their methods section, it said that acinar and duct cells were isolated by FACS as in DOI:10.1007/s00125-011-2283-5, and beta cells also by FACS as in DOI:10.1073/pnas.1713736114. Both of these publications show the FACS dot plots used, in addition to side scatter plots and immunofluorescent staining of the selection markers. The 2017 Neiman et al. paper for beta cells is from many of the same authors as the Moss 2018 paper, so we can assume it was probably done in the same lab, maybe by the same person. The Moss 2018 paper itself validated these samples in several ways, including generating in silico mixtures for each cell type individually. Their most convincing validation was the detection of beta cells in cfDNA of islet transplant patients. In these patient samples, the fraction of beta cells estimated based on methylation deconvolution using the FACS-purified cells was nearly identical to the fraction of beta cells using deep methylation sequencing of the INS promoter (pearson r=0.996, from their Figure 5). The INS promoter has been used as a highly specific methylation marker for at least a decade. Based on all of the above, it still seems nearly inconceivable to me that their FACS-sorted acinar and duct cells would have 30-40% endocrine contamination and their beta cells would have nearly 40% duct contamination. It seems much more likely that there is confusion between the duct and endocrine states of the EPISCORE model. One indication of this is that if you did have true contamination, you might expect it to vary from sample to sample, yet EPISCORE predicts the level of contamination to be very tight between replicates, almost always within 5% of the same level.

Since I could not figure out a way to resolve the inconsistencies with the FACS-sorted samples, I delved deeper into your other major validation of the pancreatic model, which are pancreatic tumors from TCGA. The results for Pancreatic Ductal Adenocarcinoma (PAAD) are strange. They prejudice a median of

~20% endocrine cells, which goes counter to the view of experts which is that PAAD tumors should contain only a very small fraction. To check this, I went through a number of the recent single-cell RNAseq studies of human PAAD. None of them found more than a few percent endocrine cells. The most recent study from 2021, Wang et al. (10.1038/s41421-021-00271-4) profiled 9 human PAAD tumors and analyzed them together with 4 PAAD and 2 adjacent pancreas from an earlier study. Their results showed that most tumors contained well under 1% endocrine cells, with the highest having 4.5% (Supplemental Table 4 from the Wang et al paper).

I looked at several other studies, all of which showed similarly low fractions of endocrine cells:
Elyada et al. 2019 (10.1158/2159-8290.CD-19-0094): scRNAseq of 6 human PDACs, and 2 adjacent normal human pancreas included in the analysis above but with their own independent clustering analysis.
Dominguez et al. 2019 (10.1158/2159-8290.CD-19-0644): scRNAseq of 22 human PDAC
Moncada et al. 2020 (10.1038/s41587-019-0392-8): 2 human PDACs with scRNAseq and spatial transcriptomics

The results in your main Figure 6 indicate that the endocrine content might be coming mostly from PP (gamma) cells, based on a deconvolution with more specific endocrine cell types. You support this by showing that expression of the PP-specific gene PPY is relatively high in TCGA PAAD tumors, and somewhat correlated with your gamma content prediction (although only pearson r=0.29). I checked TCGA and there is indeed higher PPY expression in PDAC than any other TGCA cancer type. But my suspicion is that this bulk signal is the result of very high expression in a very small fraction of cells (given PPY's role as a secreted protein, it might need to be very highly expressed).

Another thing that raises suspicion regarding your PAAD analysis is that the estimated endocrine and exocrine fractions are highly correlated along the identity line of Figure 6c. This again indicates likely confusion between cell types in your model, reminiscent of the results on FACS-sorted pancreatic cells. Clearly from this same figure, the model is good enough to distinguish PAADs from PNETs relatively well, but that is not enough to justify your positioning of this as a true deconvolution model (the title of your manuscript implies accurate deconvolution - "in silico microdissection of human tissue methylomes").

The issues above regarding the the PAAD analyses have to be resolved before I can have confidence in this method. Part of the problem with this analysis could be because, as the TCGA PAAD paper estimates, these tumors have a median of ~80% stromal content, composed mostly of cell types not represented at all in your model (fibroblast and leukocytes). So it brings into question whether it's appropriate at all to analyze PAAD tumors without adding these cell types to your model. Whether or not you make a new model including these cell types, it will be important to further validate your claim that endocrine cells make up ~20% of PAAD tumors. You can reanalyze the several datasets I listed above, or others, to validate this prediction. You are by now experts in downloading and processing

these types of scRNAseq datasets. You should look at the expression of your model's endocrine marker genes and the PPY gene within these datasets to show that endocrine cells, and PPgamma cells in particular, make up some sizable fraction of the cells consistent with the 20% estimates. It may be that all of this will make more sense when you add the stromal cell types to your model.

I am still left wondering as I did originally whether certain quality controls might help identify a potential problem with the pancreatic model. Your addition of supplemental table 6 is a good start, but it does not include much on the quality of the methylation imputation, which is the heart of EPISCORE. Is there a way to quantify the goodness of fit to the gamma distribution in the imputation model? Could you quantify how well the SCM2 vs RMAP datasets each fit the model? In the earlier Genome Biology paper, you stated, "we always observed excellent correlation between the imputed DNAm values from the two separate imputation models." These would be useful calculations to add to supplemental table 6, and might lead to insights about the pancreatic model.

Author Rebuttal, second revision:

<u>Responses to Reviewer #1:</u>

Comment: I appreciate the author's attention and time to the comments, suggestions, and critiques from the previous review. While I am mostly satisfied with the authors' responses and the additions they made to their revised manuscript, I have a two lingering comments.

Response: We thank the reviewer for taking time to re-evaluate our MS.

Comment 1. In regard to this reviewer's previous comment to "cross-compare cell-specific methylation events/effects identified using this atlas in tandem with cellDMC/TCA, when applied to bulk-tissue methylation data in a solid tissue type, to the results of single cell methylation data in the same tissue type" the authors aptly point out that such an analysis would be challenging given the type of data that would be needed for such a comparison and the corresponding dearth of such data that are available in the public domain. The authors present two additional analyses in their response to "alleviate the reviewer's concern", however these analyses do not directly address the original question. The first analysis (now panels e and f in Fig. 4) shows agreement in the cell type fractions for neuronal (Neu +) and non-neuronal (Neu -) populations while the second analysis (now, Supplementary Figure 18) depicts a direct comparison of the neuron DNAm reference profiles with DNAm profile of FACS-sorted neuron samples. Both analyses are interesting in their own right and relevant to the scope of this paper, they just don't address this reviewer's initial comment, which was to use the cellular landscape estimates from EpiSCORE in tandem with cellDMC/TCA to see if the estimated cell-specific methylation effects are consistent with what is observed purified cell populations or single cell DNA methylation data. I understand that such analysis, at least one

that is conducted with a sufficient sample size to ensure statistical rigor, is challenging based on what data are (rather are not) available in the public domain. Perhaps it is best left at that and described as a limitation of the paper or as an opportunity for future work. One final point on this front regarding the results shown in Supplementary Figure 18. The PCC is the only reported metric in this analysis and I feel that the authors should additionally report the RMSE as I suspect that the PCC is being driven by the clusters of points with high and low methylation values.

Response: Yes, the reviewer is right in pointing out that the data to test the EpiSCORE predictions in the context of a CellDMC/TCA type of analysis is simply not yet available. Although we would like to mention this in Discussion, we are limited by word counts. In any case, our MS does provide an example where we use CellDMC in conjunction with EpiSCORE (new Fig.4).

Concerning SuppFig.18 (now new SuppFig.19), we are comparing our DNAm-atlas neuron reference to the one derived from snmC-Seq data, and we agree that while the PCC is high and significant, that there are marker genes where the difference in DNAm is not small. Given the high sparsity of the snmC-Seq data and the better result obtained on FACS-sorted Neu+ samples using our DNAm-atlas, this would suggest that our reference matrix is potentially more accurate than the snmC-Seq derived one. This is the reason why we had not included the RMSE. In our sincere opinion, adding the RMSE to this SuppFig. is also not necessary, as the differences are clearly visible.

Comment 2. In the introduction, the authors state, "To address these challenges, a number of reference-based and reference-free cell-type deconvolution methods have recently been proposed". As a matter of record, I feel that the author's should include the Houseman 2012 paper as a reference here as this was the first paper to describe cellular deconvolution in the context of DNA methylation data.

Response: We sincerely apologize for this omission, as we were thinking mainly of algorithms that can detect cell-type specific signals. Houseman's original algorithm only estimates cell-type fractions in blood but does not detect cell-type specific DNAm changes. Nevertheless, we recognize the foundational work that Houseman has provided and so we now cite Houseman's 2012 paper in the introduction.

Response to Reviewer-2 comments:

Comment: I reviewed the responses to the issues I raised about the pancreas results. While it is clear to me that the EpiSCORE method can detect important cell type signals, the quantitative

accuracy of the decomposition is still questionable to me (as detailed below for pancreas examples). In their "Additional Notes" the authors point out that these estimates don't need to be absolute cell type fractions as long as they are invariant under translation and scaling. This is an important point and should be added to the Discussion so that readers/users know that these should not necessarily be interpreted as actual cell type fractions. I think without a discussion of this, many would since they are always referred to as "cell type fractions".

Response: We now alert the readers to this point in Discussion, although in the cell-type deconvolution field this is actually fairly well-known. Indeed, if for instance we take all the current-state-of-the-art cell-type deconvolution algorithms for bulk RNA-Seq data, the obtained cell-type fractions are generally speaking more of a relative measure as opposed to an absolute one despite values being constrained on the (0,1) interval. The reviewer would be wrong to think otherwise. For DNAm cell-type deconvolution algorithms in a well studied tissue like blood, cell-type fractions are definitely more interpretable as absolute values, but in general solid tissues, the first challenge is to obtain fractions that are reasonably accurate on a relative scale. The subsequent challenge would be to obtain fractions that are reasonably accurate on an absolute scale. EpiSCORE and the DNAm-atlas represent a clear advance in providing accurate cell-type fractions across a large number of different tissue-types, with the evidence in some tissues pointing towards these fractions being reasonably accurate on an absolute scale (e.g. skin, lung, brain). We agree that in the case of pancreas it is unclear if the fractions can be interpreted on an absolute scale, but it would be equally premature to claim that they are not.

In general, it has become acceptable in the field to use the term "cell-type fraction" since coining the expression "relative cell-type fraction" every single time would be quite cumbersome. We don't see any good reason for changing terminology here. Indeed, that most estimated fractions should be interpreted in a relative sense is particularly true for IHC-based "cell-type fractions" which this reviewer seems to be invoking as a gold-standard.

Comment: The authors did not perform the one in silico validation that I suggested in the prior rounds of review, which was to validate the 20% endocrine and/or 20% gamma cell fraction in typical pancreatic ductal adenocarcinoma (PDAC/PAAD) tumors. This relatively high fraction of endocrine content is provocative and surprising, and I think deserve to be validated since the data exist. The gamma cell claim in particular could be validated by looking at the PPY gene in the scRNA-seq PDAC/PAAD datasets (I do not find the TCGA expression results very convincing).

Response: We wholeheartedly disagree with the reviewer that these analyses on PDAC scRNA-Seq datasets would be conclusive. First of all, we would not be testing the pancreas DNAm reference at all, only the mRNA-one. Second, scRNA-Seq assays are not a gold-standard for estimating cell-type fractions! In fact, they can be extremely biased at estimating cell-type fractions! Third, the TCGA samples are an entirely different cohort for which there is NO matched scRNA-Seq data. The TCGA samples contain at least 5% PNETs and hence it is extremely likely that a considerable fraction of these tumors may also lie on a continuum in between PDACs and PNETs. This reviewer seems to think (falsely so) that pancreatic tumors are binary entities, when this "binarization" is irrational and imposed by histopathologists to ascertain a diagnosis.

In response to the reviewer's point, we have now analyzed the data from Moncada et al (the only study which has provided the human scRNA-Seq in a public repository!), and this has

confirmed that in one of the two patients, the few endocrine cells all express high levels of PPY, whilst in the other patient, PPY expression is absent (see panel-c) below:

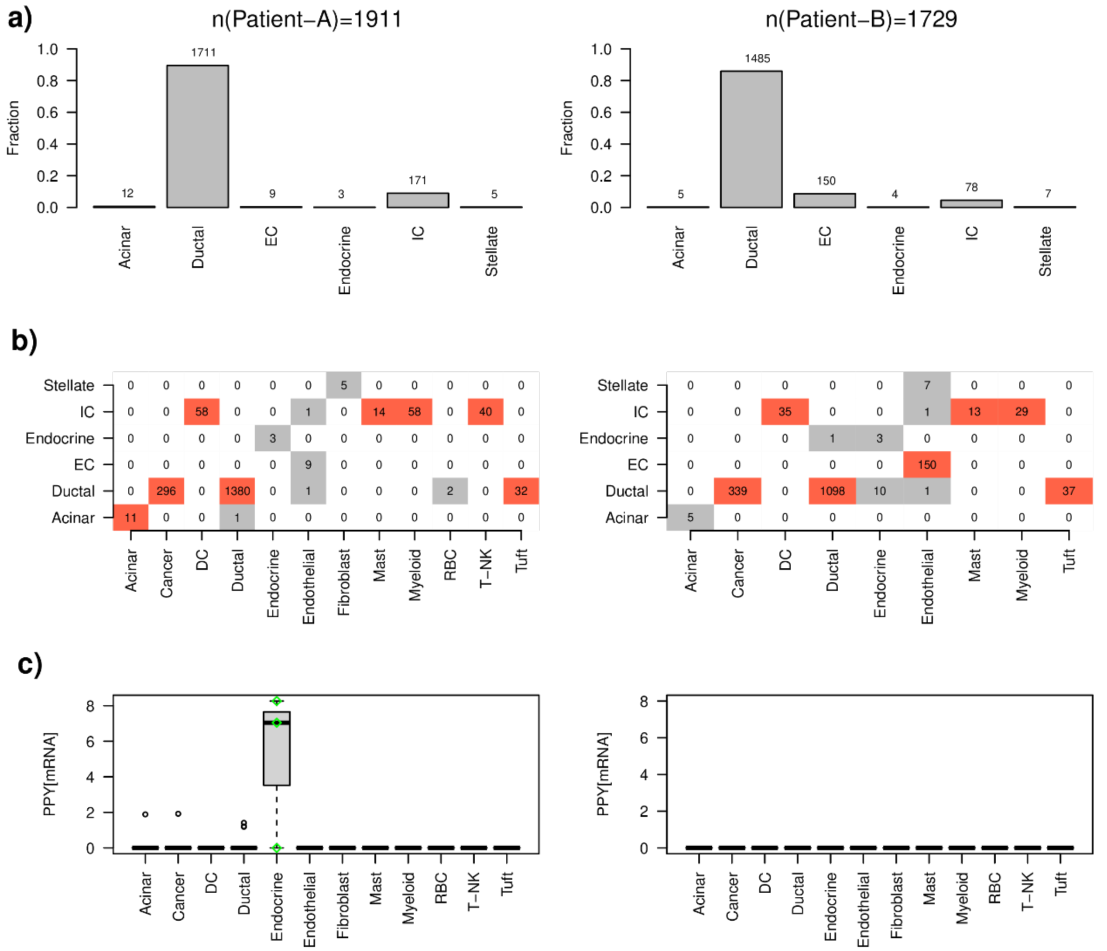

<u>Figure legend:</u> ***a)*** *Estimated cell-type fractions in PDAC patients A and B from Moncada R et al Nat Biotechnology 2020, as obtained by applying our pancreas mRNA expression reference matrix to their scRNA-Seq data, annotating cells to cell-types using a maximum-weight criterion.The numbers of cells are given above bars.* ***b)*** *Confusion matrix between our predicted cell-type annotation (y-axis) and the annotation provided by Moncada (x-axis). IC=Immune Cell, EC=Endothelial cell. ICs include myeloid, Dendritic cells (DCs), T-NK cells and Mast cells.* ***c)*** *Boxplots of PPY expression (a gamma-cell marker) against cell-type in the same Moncada scRNA-Seq data.*

This suggests that there could be significant variation in the endocrine composition between patients and that the gamma-cell fraction could indeed be the dominant endocrine fraction in some PDACs, consistent with our findings in Fig.6b.

The analysis on Moncada et al also confirms a very low endocrine fraction (see panel-a) but at the same time also very low fractions for fibroblasts and endothelial cells, which is very unexpected since CAFs are very abundant in PDACs, vindicating our point that scRNA-Seq

assays can not be used to estimate cell-type fractions. An explanation for these findings is that the non-malignant ductal fraction (72%) in the Moncada samples is huge and therefore these samples would never be comparable to the TCGA ones where the non-malignant ductal proportion is under 10%.
In our opinion, the key point here is that the TCGA cohort is a different sample collection to those used in these scRNA-Seq studies, so the reviewer can't transfer findings from studies profiling a small number of PDAC samples to the findings obtained in the larger TCGA cohort. The above figure has been included as new SuppFig.S22.

Comment: Without these validations, my feeling is that the term "microdissection" in the title and the abstract connote a degree of resolution and accuracy that has not been demonstrated. I think something more neutral would be appropriate, such as "deconvolution" or "decomposition".
Response: As requested, we have changed "microdissection" into "decomposition".

Detailed responses follow:
Comment: The new arguments presented about the impurity of the published FACS-sorted pancreas validation samples do not help convince me of the accuracy of the decomposition. The INS promoter analysis takes the most favorable assumption that beta cells have 0% methylation and still predicts a contamination level of 20% in the beta cell samples, while the EpiSCORE model predicts 40-60% non-beta cells for all four of these samples.
Response: We disagree. If say a FACS-sorted beta-cell is only 60% pure with 40% contamination by another cell-type, say ductal cells, then if an algorithm predicts that this sample is 60% beta-cells and 40% ductal cells, then this is correct. In other words, the purity of the FACS-sorted samples matters when assessing our pancreas DNAm reference matrix. In this particular instance, we have estimated 80% purity for the beta-samples of Moss et al, and so we agree that there is still a residual discrepancy of 20%. However, these are only 4 samples. You can't accurately estimate a mean proportion from only 4 samples.

Comment: The authors argue that four samples is too small to conclude anything, and that probably the accuracy would go up if they had more samples. I disagree and think the variance is small enough among the four samples to make a judgement. The levels of beta, ductal, and stellate cells predicted are all very tight, and this would be unlikely to change with additional samples.
Response: We disagree. We see a lot of variation (i.e. 20% variation if not more) in sample purity between samples from **different** cohorts. That the distribution is tight for replicates within the same study makes a lot of sense because these samples were all processed equally and are therefore potentially subject to the same biases! However, if we were to compare to another study using a different better protocol to purify samples then this may well highlight 20% variations. This reviewer does not fully understand that FACS-sorting does not guarantee high purity, and indeed a plethora of scRNA-Seq studies are now clearly demonstrating that FACS-sorting based on traditional markers does not generate pure cell populations. For instance, work from Ranzoni & Cvejic Cell Stem Cell 2021, have used scRNA-Seq to improve marker panels for generating purer cell populations in the hematopoietic system, and we expect similar improvements to be made in more complex tissues like pancreas. The reviewer is prejudiced on a false inherent belief that FACS-sorted samples from solid tissues have over 95% purity.

Comment: *The authors also try to argue about the degree of contamination in the acinar and duct sorted cells based on the INS promoter, but I think this is even more dubious. They assume that these cell types would have close to 100% methylation, which is never a good assumption since very few regions in the genome are that high. In support of this, the Neiman figures C and D show pyrosequencing of the INS promoter for total pancreas, which shows ~80% methylation even though total pancreas is only about 1% total endocrine cells. It is also clear from your plot of all 450k probes in the genome, which show that the mode of the upper peak is 80-90% methylation.*

Response: We disagree. In a given cell at a given allele, DNAm is BINARY (0,1). When in an assay we measure at at given locus a 20% DNAm level, this means that 20% of the cells in the sample have that locus methylated. If we assume that the FACS-sorted beta sample has 100% purity, then the DNAm level for the INS promoter should be close to 0 (in practice around 1-2% because of technical bias). If the Neiman figures C and D indicate 80% DNAm at INS promoter for total pancreas this could mean that the beta-cell fraction in total pancreas has been underestimated, consistent with our findings.

Once again, this reviewer has also ignored our important comment regarding skews in the experimental assays. If we perform pyrosequencing on total pancreas, there is no guarantee that such an assay would not have experimental biases/skews towards specific cell-types which could also explain 10-20% shifts in cell-type proportions. In our last letter we clearly demonstrated how these experimental skews are present in other tissues (e.g. cervical smears), so it is very plausible that such experimental skews are also present in DNAm assays performed on pancreatic tissue.

*Comment: Regarding the PAAD analysis, I completely accept that the EpiSCORE model is able to classify the ~5% of TCGA PAAD samples that are PNET or PNET-like. Solving this classification problem is not the same as providing accurate cell type decomposition. My issue was not with these outlier samples but with the prediction that the **median** TCGA PAAD sample had ~20% endocrine fraction in the 6-state model (Supp Fig 20B) and ~30% endocrine fraction in the 9-state model (20% gamma cell and 10% alpha cell, Fig 6C). These predictions are provocative and very surprising. You cited a potential reason that endocrine islets might co-localize to pancreatic cancer epithelial cells, but this would not explain why the adjacent normals have similar predicted endocrine fraction in both the 6-state (Supp Fig 20B) and 9-state (Fig 6C) models. More importantly, the data exist to actually test this. As I showed last time in the bar chart I produced from the Supplemental Table 4 of Wang et al. 2021 (10.1038/s41421-021-00271-4), 14 of 16 human PDAC samples from two independent studies had around or less than 0.5% endocrine cells. This is consistent with the roughly 1% endocrine cells present in normal pancreas. Very low levels of endocrine cells were found in two additional high profile human PDAC scRNA-seq studies that I cited in my previous comments.*

Response: We have already responded to this point earlier, when discussing the results we have obtained in Moncada et al, one of the scRNA-SEQ PDAC datasets the reviewer was referring to.

Comment: The authors provide an additional argument that scRNA-seq can bias or miss

particular cell types. I think it's very dangerous to pick and choose validation datasets based on whether they agree with your results.

Response: First of all, it is unequivocal that scRNA-Seq assays DO BIAS cell-type proportions. Only when comparing very similar cell-types to each other would it be justified to draw conclusions from scRNA-Seq data. We also mentioned adipocytes in breast tissue as a concrete example, as adipocytes a major component of breast tissue are completely missed by 10X assays. The reviewer offers no counterargument to this. Second, we are not cherry-picking studies. In fact, our paper is about validating a DNAm-atlas, NOT an mRNA-one. We used the TCGA data for the simple reason that this study has many samples with DNAm profiles. One of the co-authors on our MS, Prof Chrissie Thirlwell, is an authority on pancreatic tumors, and we use one of the published PNET datasets she has generated. In summary, we are using the DNAm data that is currently available.

Comment: The EpiSCORE method itself is based on single-cell RNA-seq, and single-cell data are used for validation in other datasets in this paper.

Response: The reviewer is not paying attention to the fact that the single-cell RNA-Seq datasets that we are using in our MS for validation, are being used to validate the mRNA-expression reference matrices. Because the cells in these scRNA-Seq studies have been previously annotated to specific cell-types, we can use them to check that our mRNA expression reference matrices are accurate enough. And indeed, this is how we have validated the pancreas mRNA expression reference matrix: as shown in SuppFig.6, the accuracy is over 95%!

We have now also applied the same mRNA expression reference to the PDAC scRNA-Seq dataset from Moncada, achieving over 99% accuracy. This has also clearly confirmed that the cell-type fractions derived from scRNA-Seq assays can't be used as a gold-standard. A conclusive analysis would require scRNA-Seq data matched to the SAME PDAC tumors of the TCGA. Again, we note that the TCGA cohort is a much bigger cohort and diagnosis of PDACs/PAADs was not done at the same level as these recent scRNA-Seq studies.

Final Decision Letter:

Subject: Decision on Nature Methods submission NMETH- RS46537D
Message:

28th Jan 2022

Dear Dr Teschendorff,

I am pleased to inform you that your Resource, "A pan-tissue DNA methylation atlas enables in-silico microdissection of human tissue methylomes at cell-type resolution", has now been accepted for

publication in Nature Methods. Your paper is tentatively scheduled for publication in our March print issue, and will be published online prior to that. The received and accepted dates will be 11th July 2021 and 28th January 2022. This note is intended to let you know what to expect from us over the next month or so, and to let you know where to address any further questions.

Acceptance is conditional on the data in the manuscript not being published elsewhere, or announced in the print or electronic media, until the embargo/publication date. These restrictions are not intended to deter you from presenting your data at academic meetings and conferences, but any enquiries from the media about papers not yet scheduled for publication should be referred to us.

In approximately 14 business days you will receive an email with a link to choose the appropriate publishing options for your paper and our Author Services team will be in touch regarding any additional information that may be required.

Please note that Nature Methods is a Transformative Journal (TJ). Authors may publish their research with us through the traditional subscription access route or make their paper immediately open access through payment of an article-processing charge (APC). Authors will not be required to make a final decision about access to their article until it has been accepted. Find out more about Transformative Journals

Authors may need to take specific actions to achieve compliance with funder and institutional open access mandates. For submissions from January 2021, if your research is supported by a funder that requires immediate open access (e.g. according to Plan S principles) then you should select the gold OA route, and we will direct you to the compliant route where possible. For authors selecting the subscription publication route our standard licensing terms will need to be accepted, including our self-archiving policies. Those standard licensing terms will supersede any other terms that the author or any third party may assert apply to any version of the manuscript.

You will not receive your proofs until the publishing agreement has been received through our system.

If you have any questions about our publishing options, costs, Open Access requirements, or our legal forms, please contact ASJournals@springernature.com

Your paper will now be copyedited to ensure that it conforms to Nature Methods style. Once proofs are generated, they will be sent to you electronically and you will be asked to send a corrected version within 24 hours. It is extremely important that you let us know now whether you will be difficult to contact over the next month. If this is the case, we ask that you send us the contact information (email, phone and fax) of someone who will be able to check the proofs and deal with any last-minute problems.

If, when you receive your proof, you cannot meet the deadline, please inform us at rjsproduction@springernature.com immediately.

Once your manuscript is typeset and you have completed the appropriate grant of rights, you will receive a link to your electronic proof via email with a request to make any corrections within 48 hours. If, when you receive your proof, you cannot meet this deadline, please inform us at rjsproduction@springernature.com immediately.

Once your paper has been scheduled for online publication, the Nature press office will be in touch to confirm the details.

If you have posted a preprint on any preprint server, please ensure that the preprint details are updated with a publication reference, including the DOI and a URL to the published version of the article on the journal website.

Once your paper has been scheduled for online publication, the Nature press office will be in touch to confirm the details.

Content is published online weekly on Mondays and Thursdays, and the embargo is set at 16:00 London time (GMT)/11:00 am US Eastern time (EST) on the day of publication. If you need to know the exact publication date or when the news embargo will be lifted, please contact our press office after you have submitted your proof corrections. Now is the time to inform your Public Relations or Press Office about your paper, as they might be interested in promoting its publication. This will allow them time to prepare an accurate and satisfactory press release. Include your manuscript tracking number NMETH-RS46537D and the name of the journal, which they will need when they contact our office.

About one week before your paper is published online, we shall be distributing a press release to news organizations worldwide, which may include details of your work. We are happy for your institution or funding agency to prepare its own press release, but it must mention the embargo date and Nature Methods. Our Press Office will contact you closer to the time of publication, but if you or your Press Office have any inquiries in the meantime, please contact press@nature.com.

To assist our authors in disseminating their research to the broader community, our SharedIt initiative provides you with a unique shareable link that will allow anyone (with or without a subscription) to read the published article. Recipients of the link with a subscription will also be able to download and print the PDF.
As soon as your article is published, you will receive an automated email with your shareable link.

You can now use a single sign-on for all your accounts, view the status of all your manuscript submissions and reviews, access usage statistics for your published articles and download a record of your refereeing activity for the Nature journals.

Nature Research journals encourage authors to share their step-by-step experimental protocols on a protocol sharing platform of their choice. Nature Research's Protocol Exchange is a free-to-use and open resource for protocols; protocols deposited in Protocol Exchange are citable and can be linked from the published article. More details can found at www.nature.com/protocolexchange/about.

Please note that you and any of your coauthors will be able to order reprints and single copies of the issue containing your article through Nature Research Group's reprint website, which is located at http://www.nature.com/reprints/author-reprints.html. If there are any questions about reprints please send an email to author-reprints@nature.com and someone will assist you.

Please feel free to contact me if you have questions about any of these points.

Best regards,
Lei

Lei Tang, Ph.D.
Senior Editor
Nature Methods
